# Supplementary figures and images for: The rhizobial effector NopT targets Nod factor receptors to regulate symbiosis in Lotus japonicus (part 1 of 2)
Source: eLife. 2025 Apr 4;13:RP97196. doi: 10.7554/eLife.97196 (PMC11970910; doi:10.7554/eLife.97196)

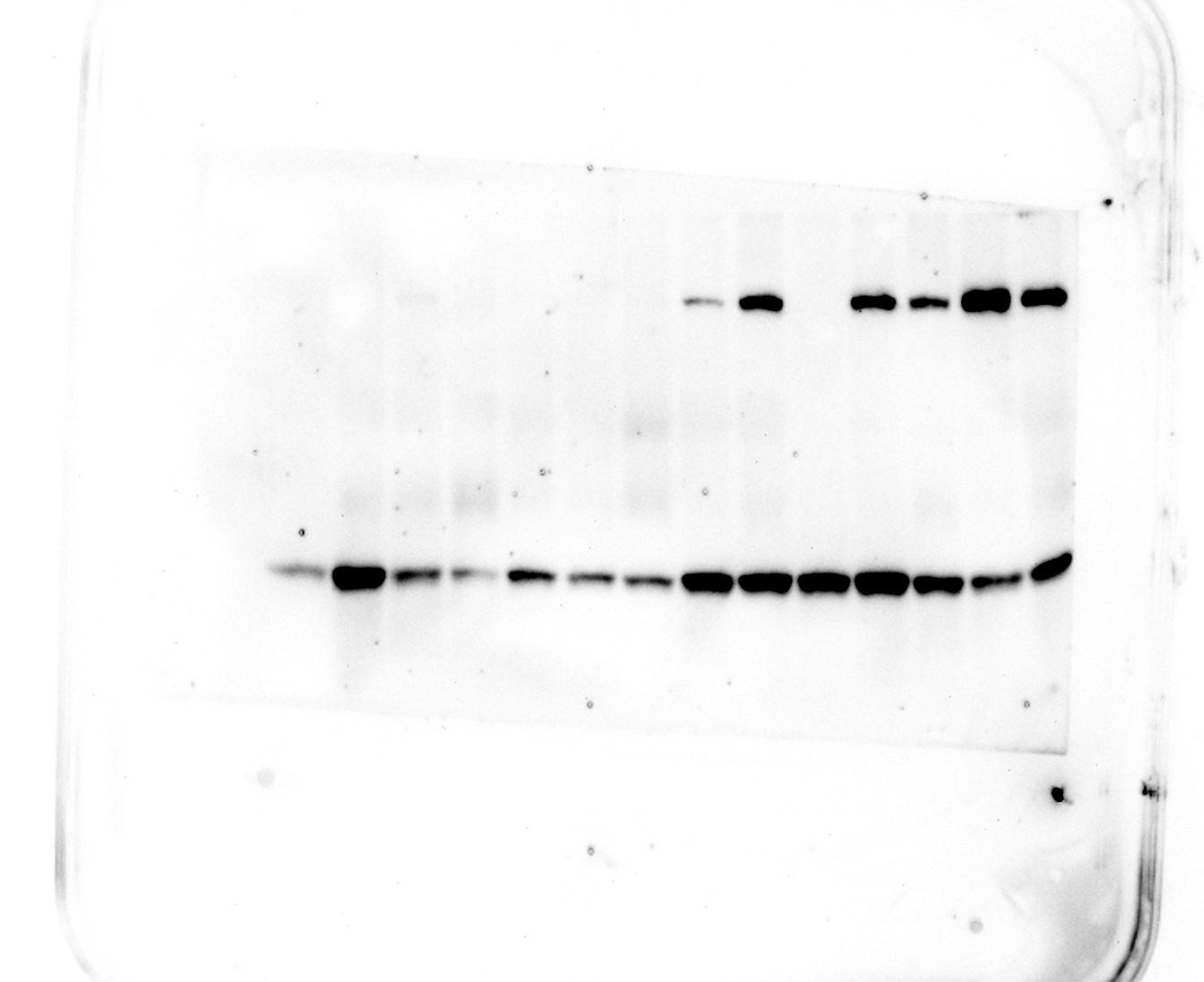

Supplement: Figure 1—figure supplement 1—source data 1. [file elife-97196-fig1-figsupp1-data1.zip › Figure 1-figure supplement 1E a-myc & a-actin.tif]

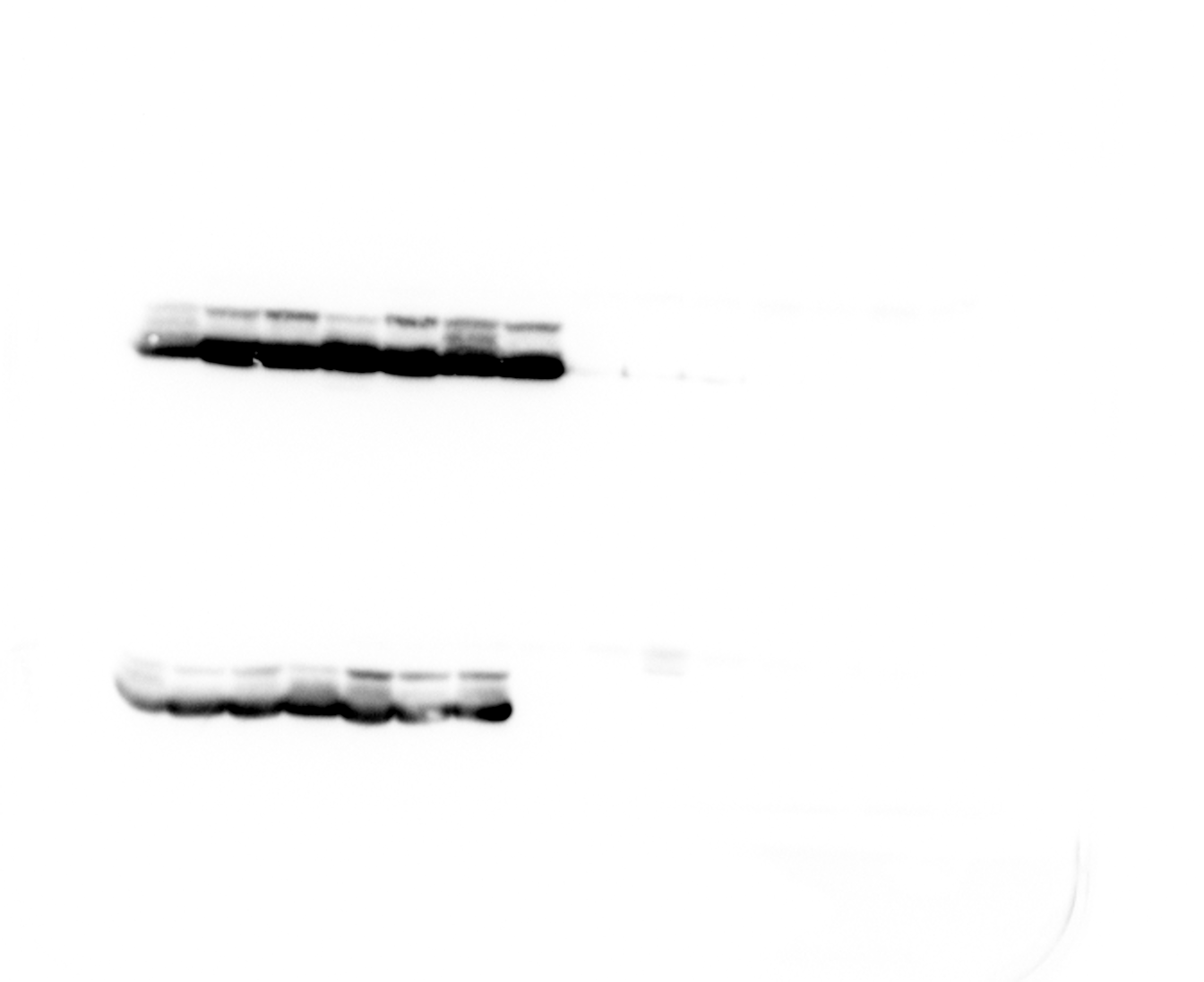

Supplement: Figure 1—figure supplement 1—source data 1. [file elife-97196-fig1-figsupp1-data1.zip › Figure 1-figure supplement 1E a-strep .tif]

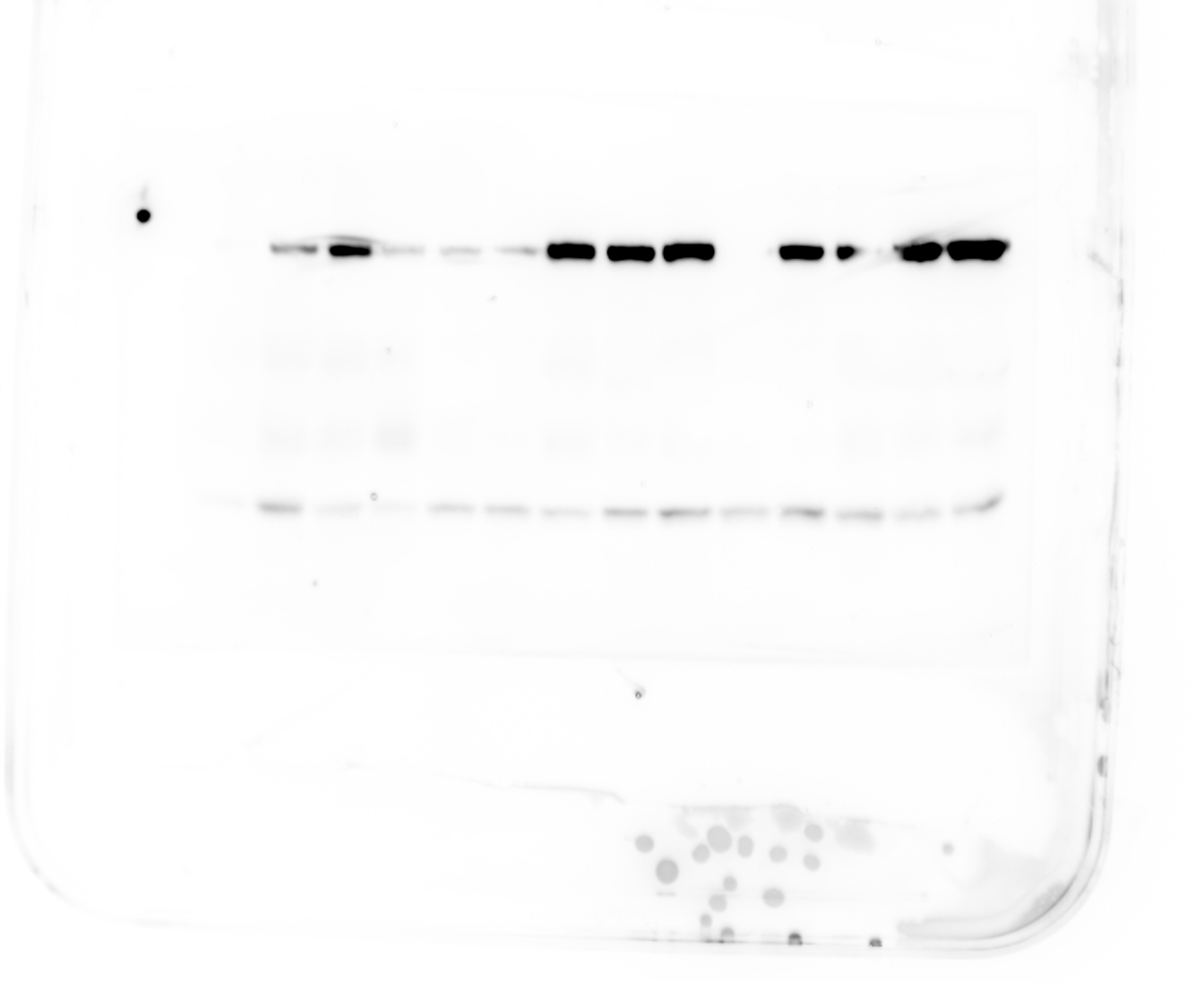

Supplement: Figure 1—figure supplement 1—source data 1. [file elife-97196-fig1-figsupp1-data1.zip › Figure 1-figure supplement 1E a-ha nb 1hao 10min.tif]

Figure 1-figure supplement 1E

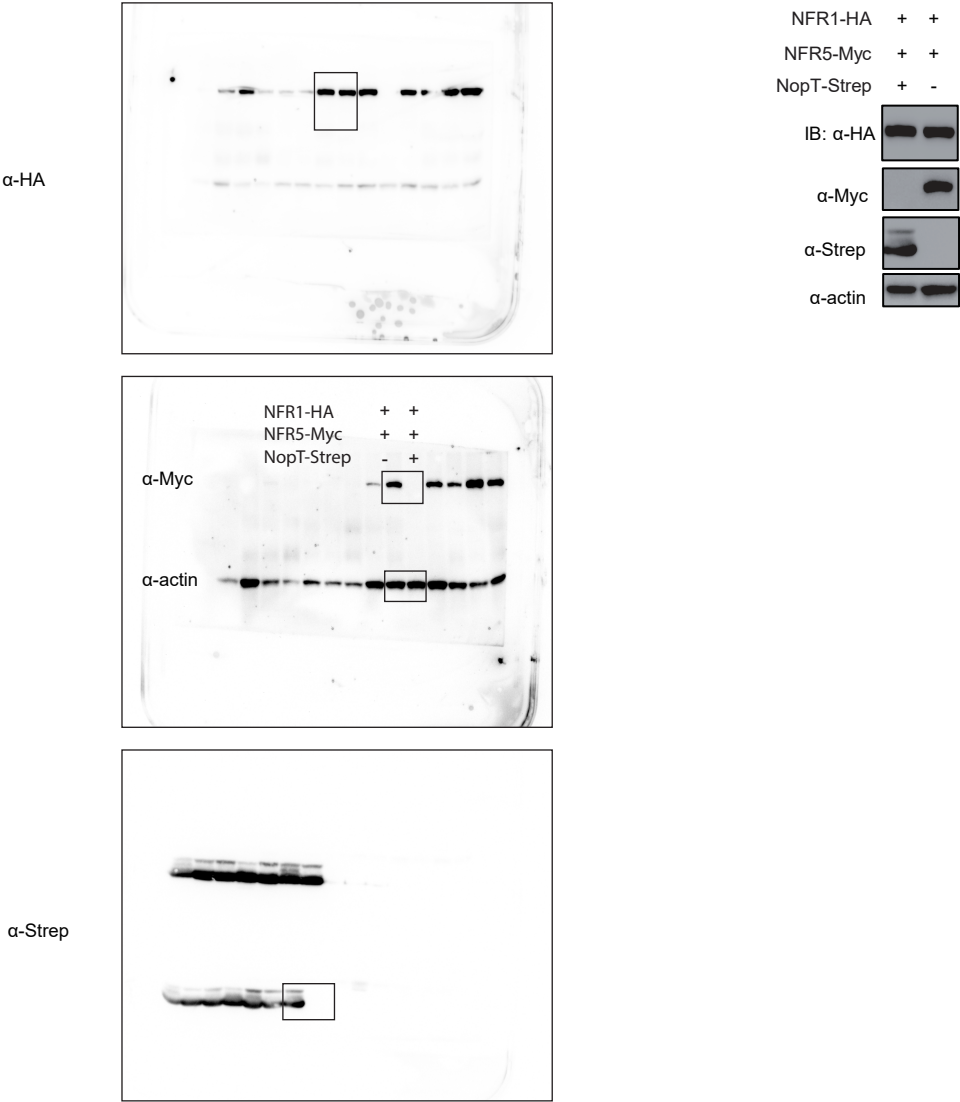

Supplement: Figure 1—figure supplement 1—source data 2. [file elife-97196-fig1-figsupp1-data2.pdf]

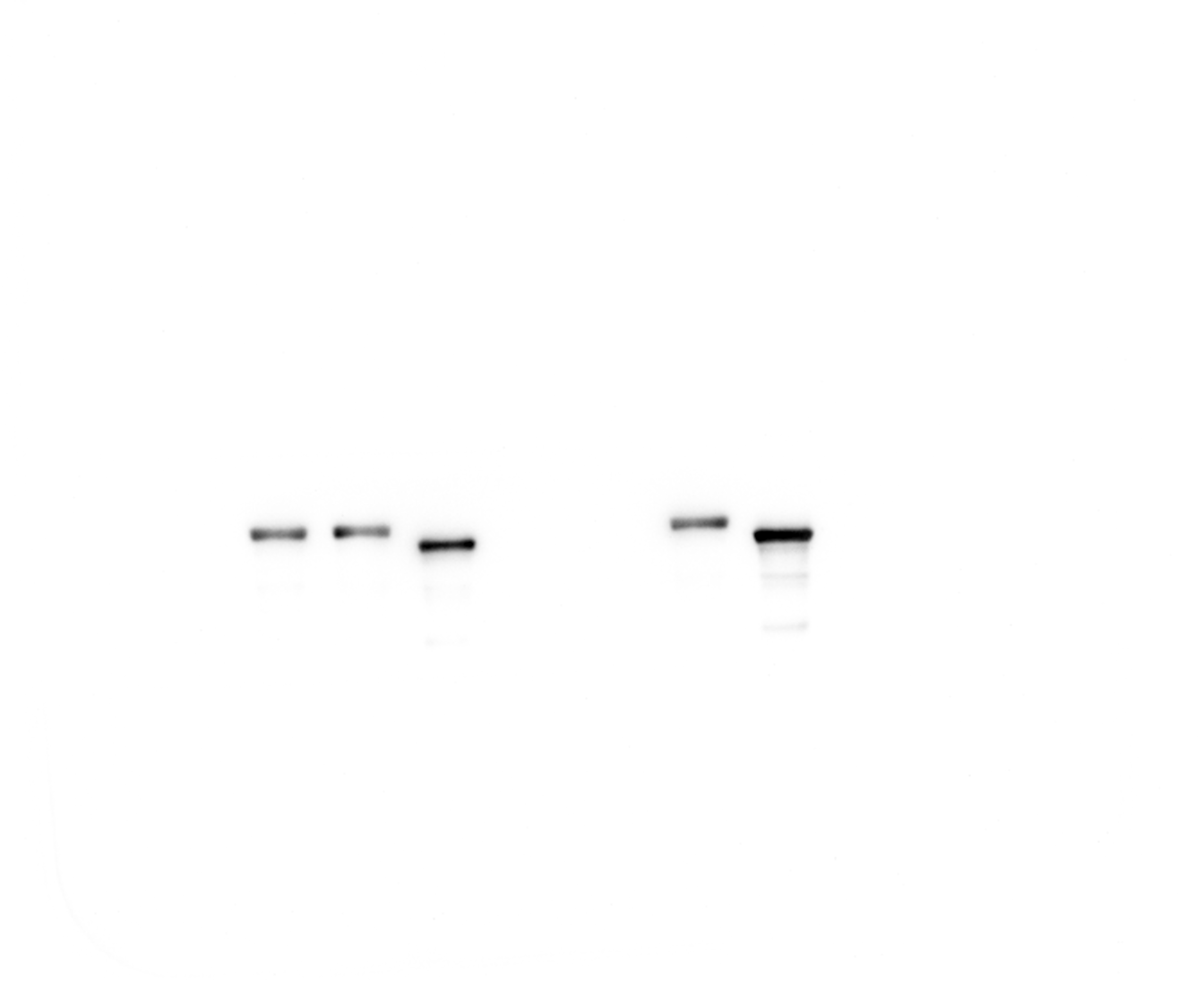

Supplement: Figure 2—source data 1. [file elife-97196-fig2-data1.zip › fig2D data1 a-ha in and out.tif]

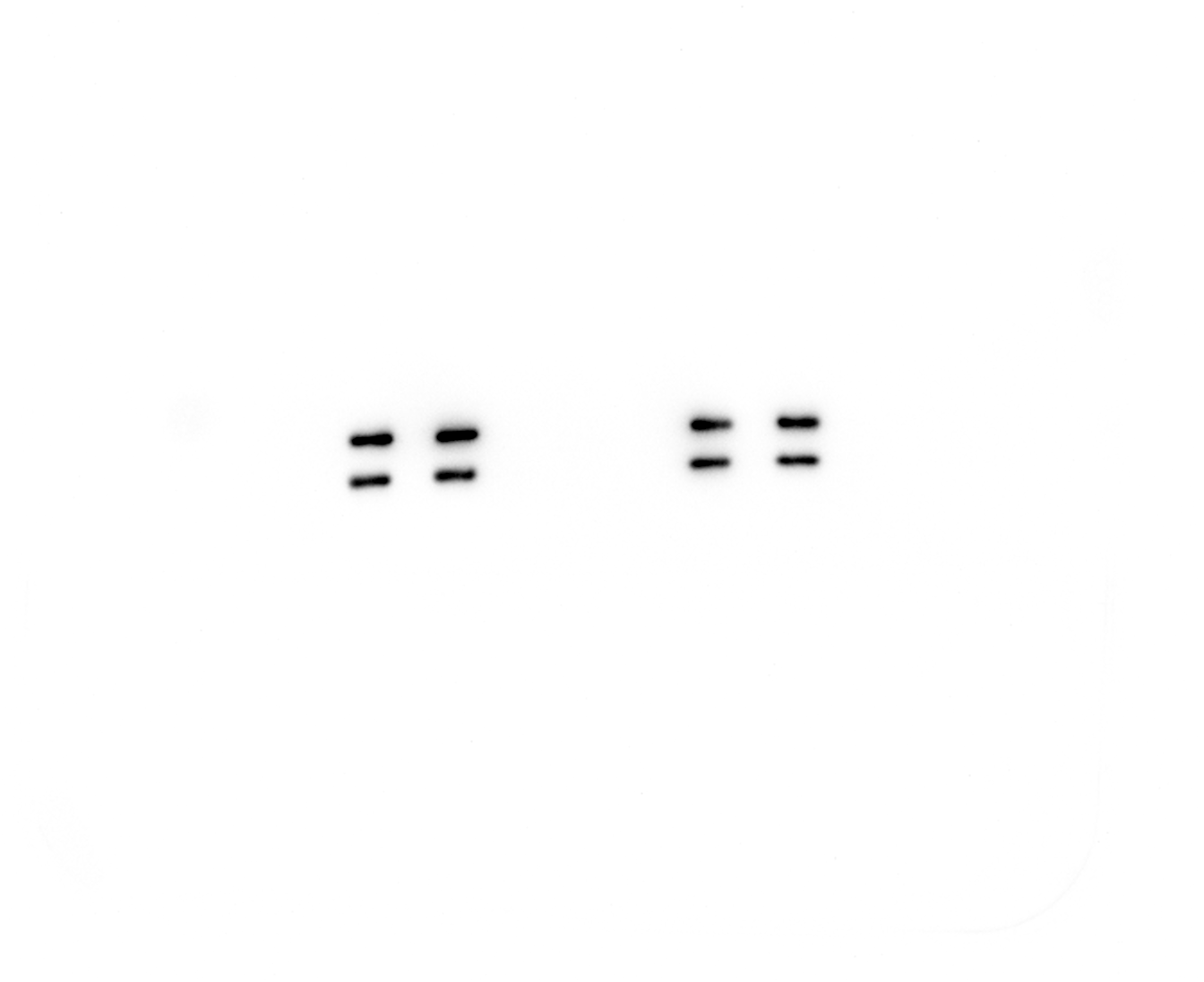

Supplement: Figure 2—source data 1. [file elife-97196-fig2-data1.zip › fig2D data1 a-flag in and out .tif]

Fig. 2D

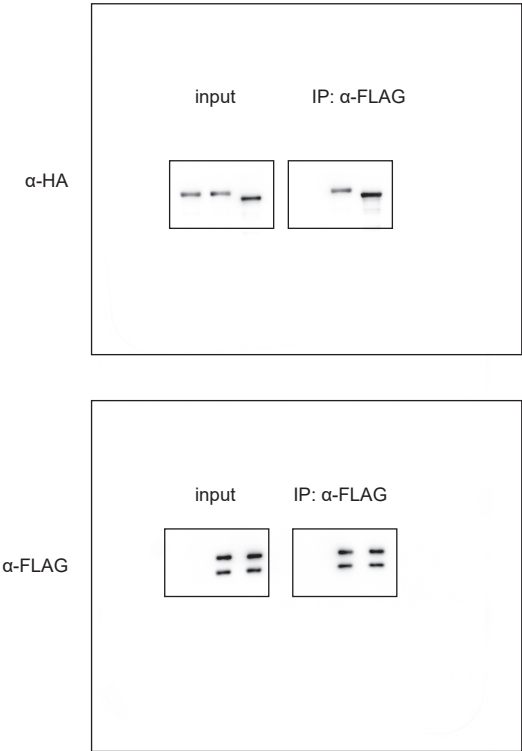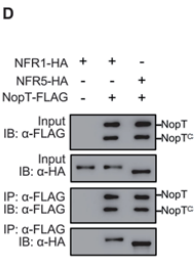

Supplement: Figure 2—source data 2. [file elife-97196-fig2-data2.pdf]

Figure 2-figure supplement 1

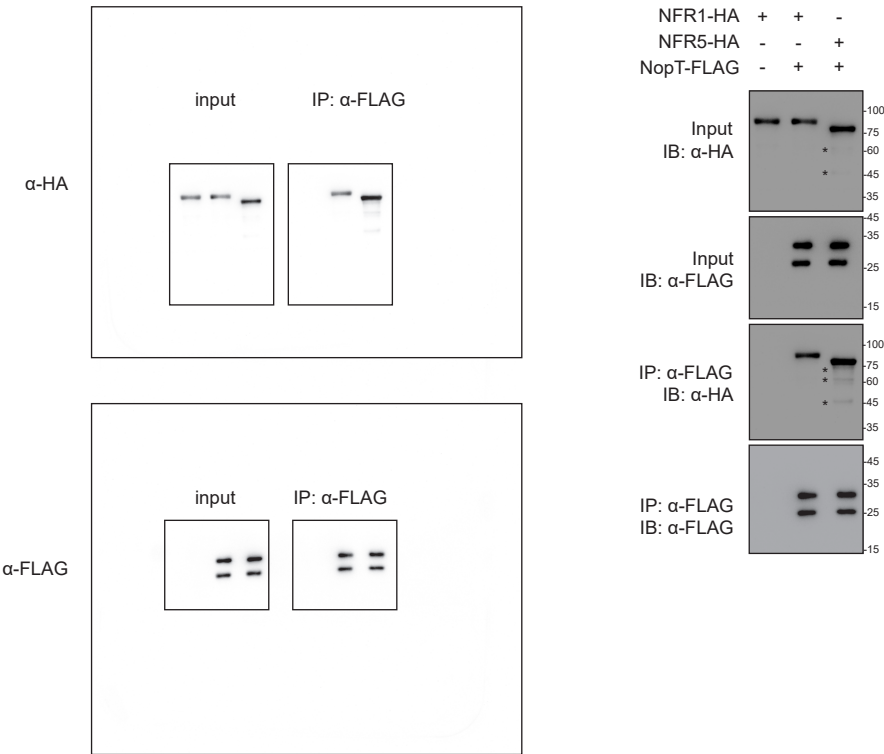

Supplement: Figure 2—figure supplement 1—source data 2. [file elife-97196-fig2-figsupp1-data2.pdf]

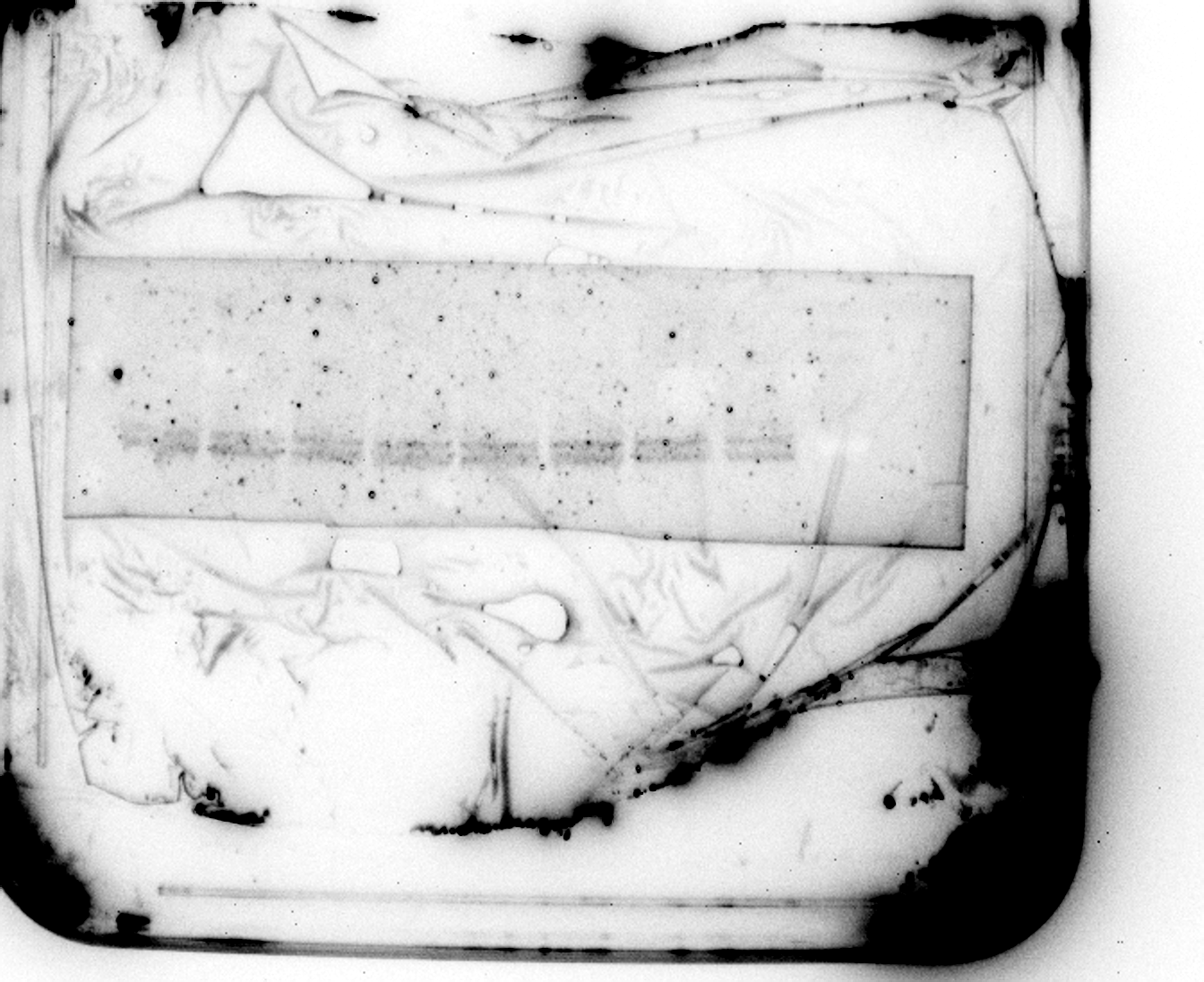

Supplement: Figure 2—figure supplement 2—source data 1. [file elife-97196-fig2-figsupp2-data1.zip › Figure 2-figure supplement 2B a- ha.tif]

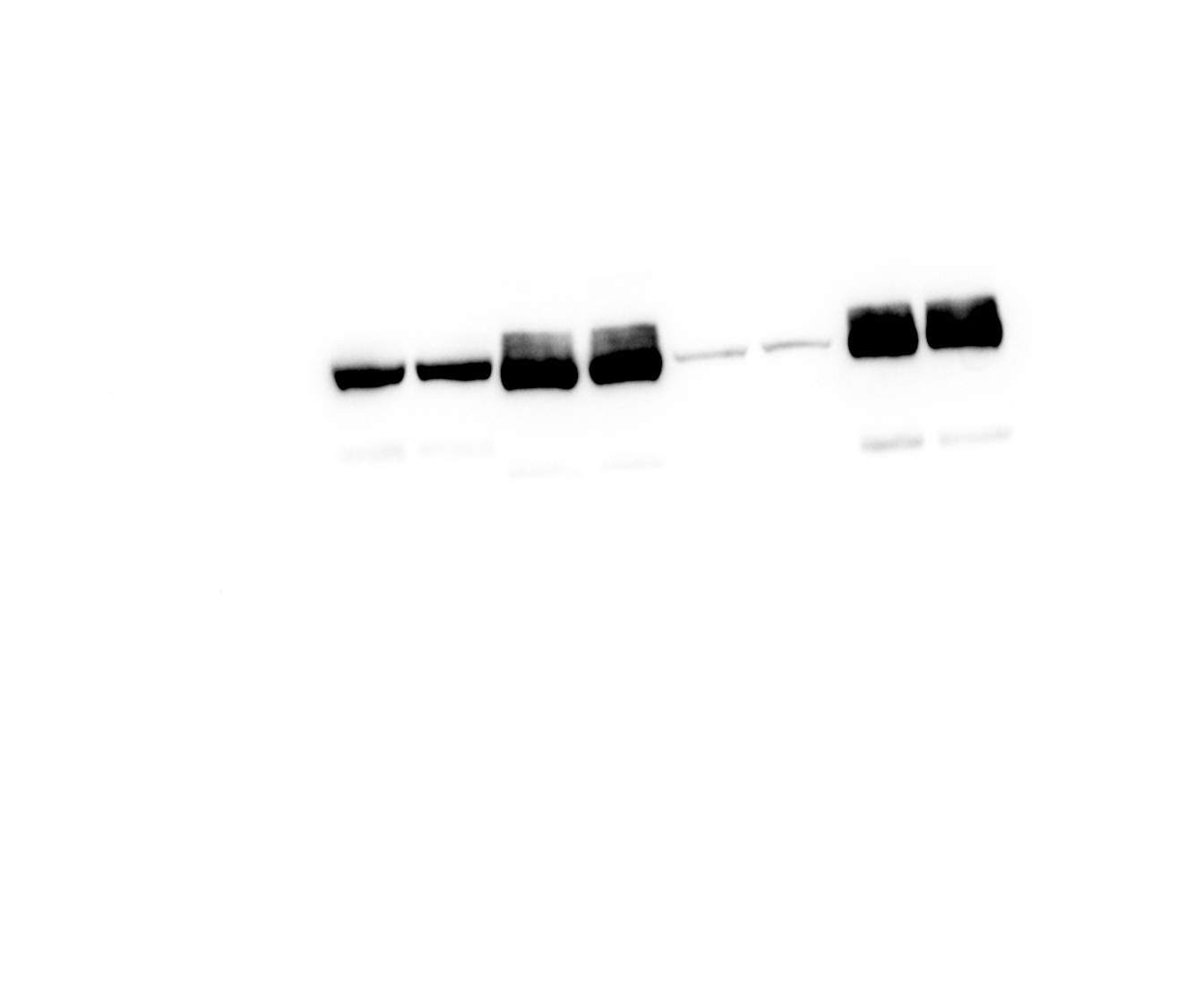

Supplement: Figure 2—figure supplement 2—source data 1. [file elife-97196-fig2-figsupp2-data1.zip › Figure 2-figure supplement 2B a- myc .tif]

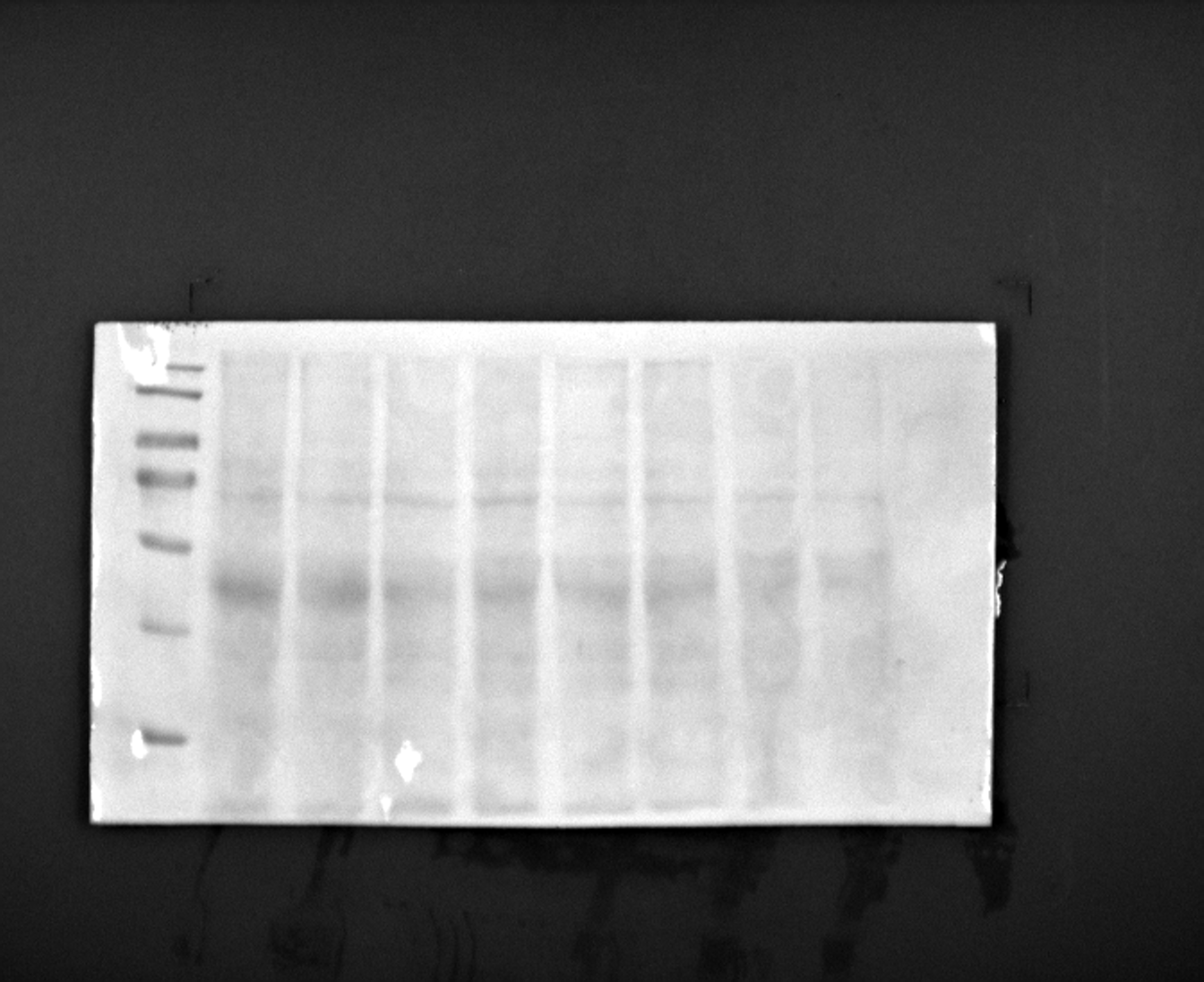

Supplement: Figure 2—figure supplement 2—source data 1. [file elife-97196-fig2-figsupp2-data1.zip › Figure 2-figure supplement 2B Ps.tif]

Figure 2-figure supplement 2B

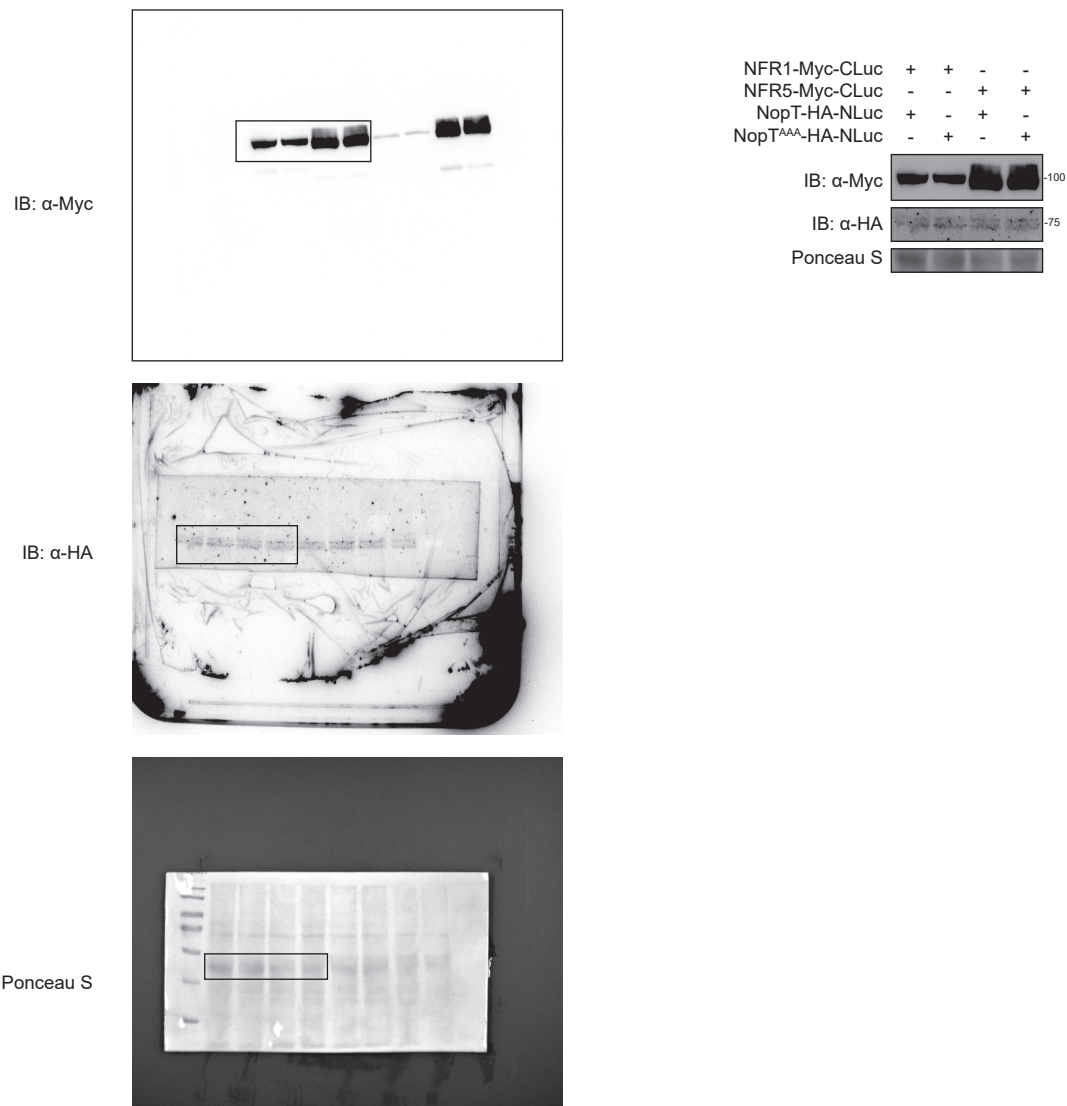

Supplement: Figure 2—figure supplement 2—source data 2. [file elife-97196-fig2-figsupp2-data2.pdf]

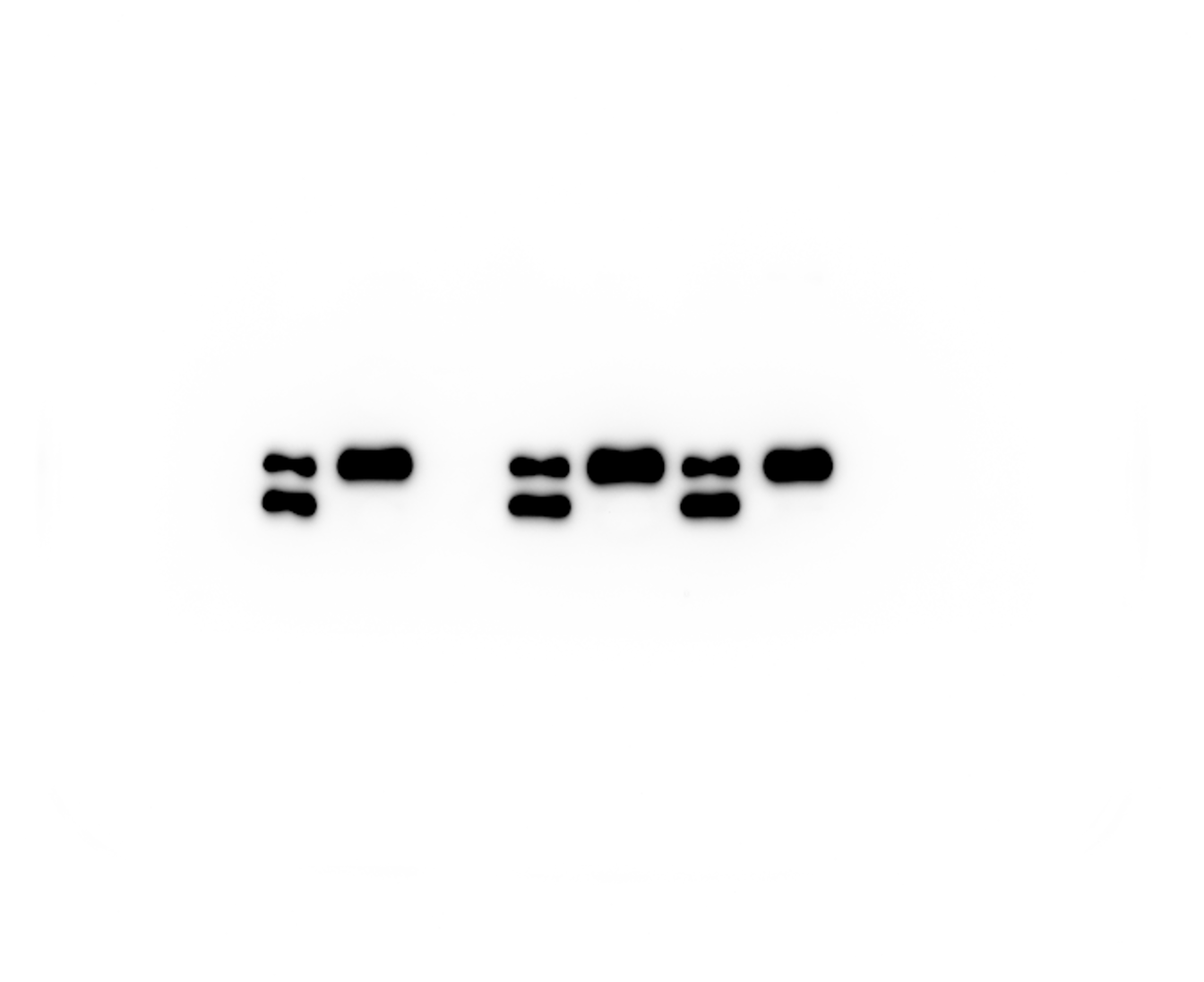

Supplement: Figure 3—source data 1. [file elife-97196-fig3-data1.zip › Figure3-SourceData1/Figure3D/FIG3D a-flag.tif]

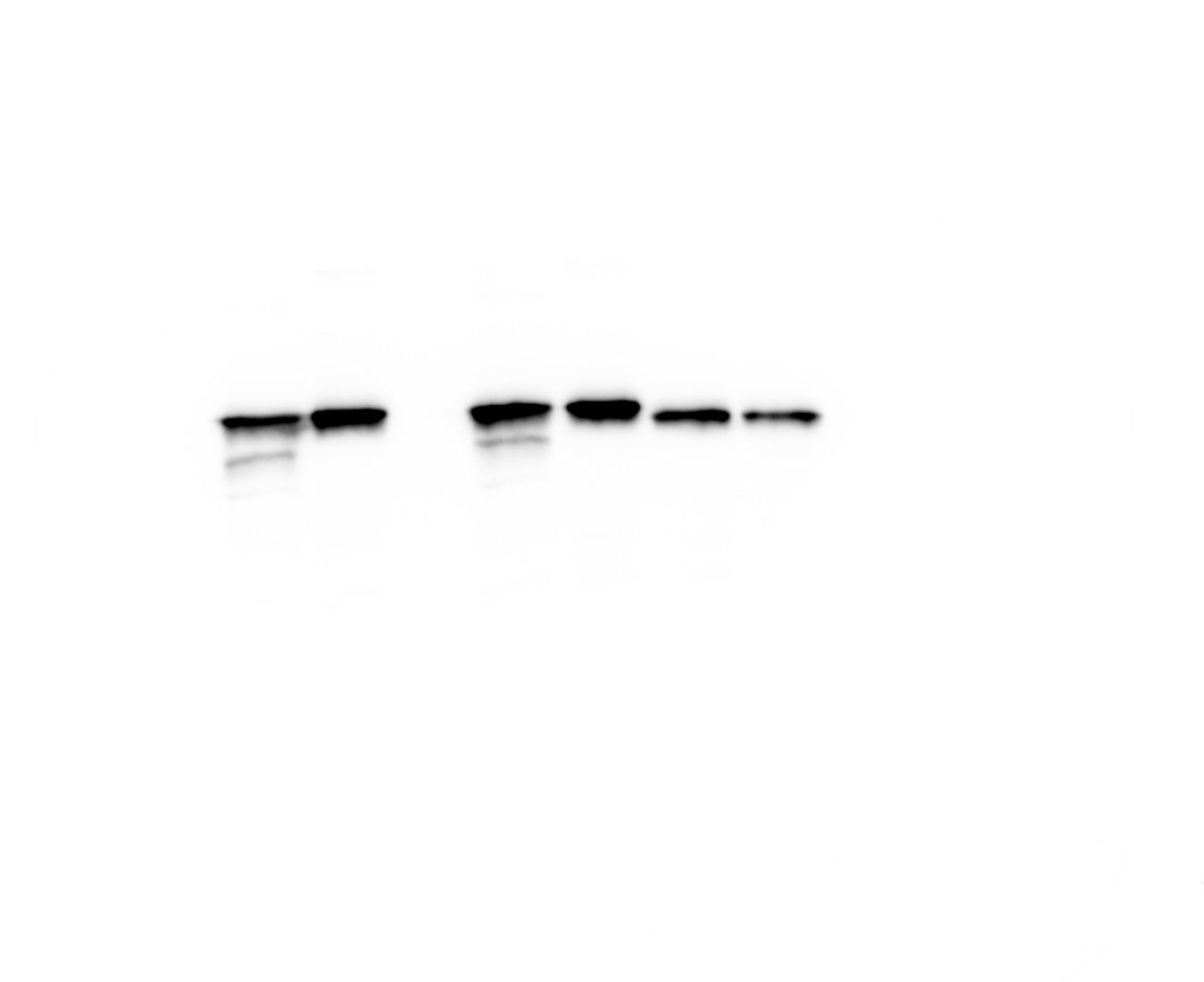

Supplement: Figure 3—source data 1. [file elife-97196-fig3-data1.zip › Figure3-SourceData1/Figure3D/FIG3D a-ha.tif]

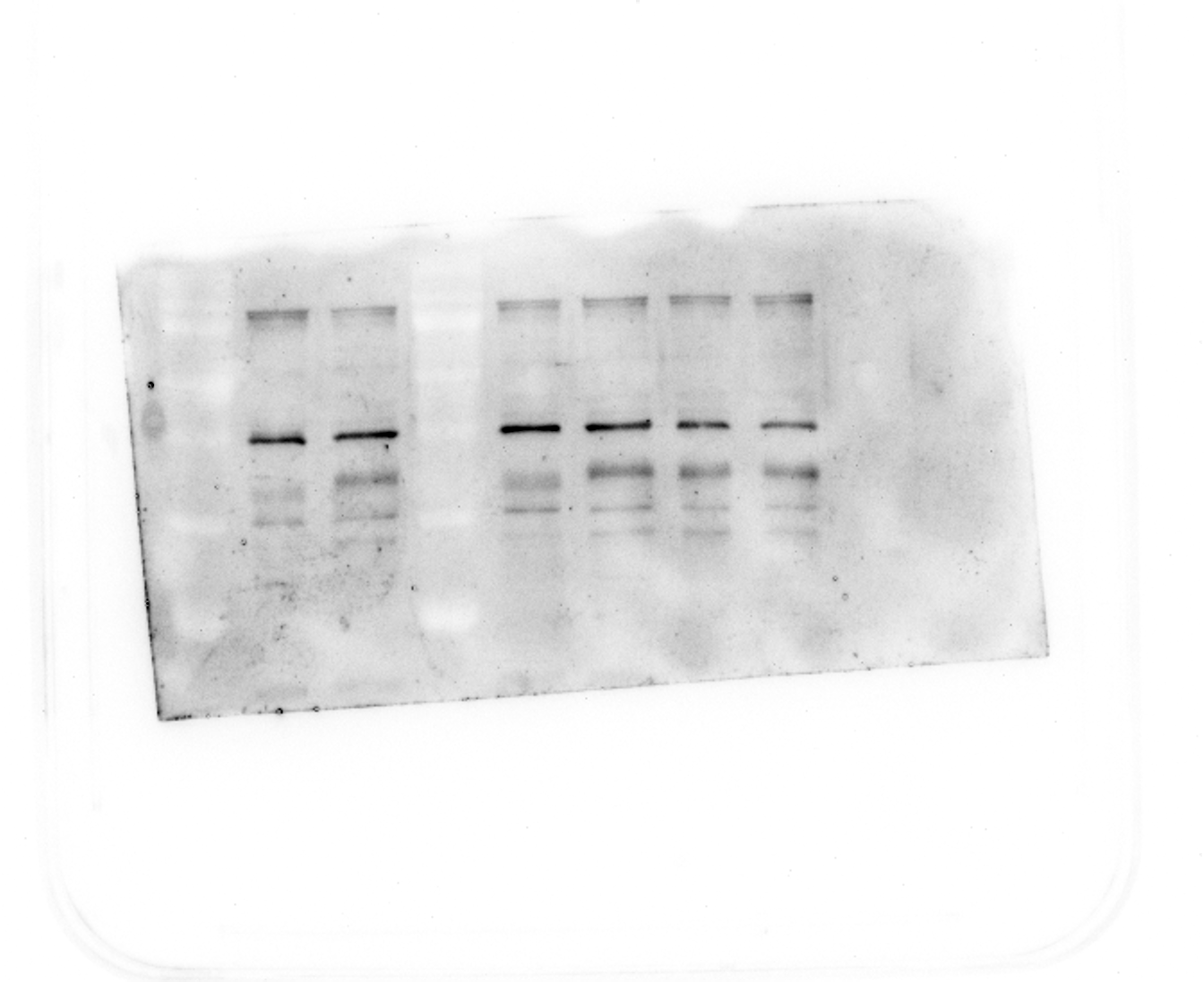

Supplement: Figure 3—source data 1. [file elife-97196-fig3-data1.zip › Figure3-SourceData1/Figure3D/FIG3D a-groel .tif]

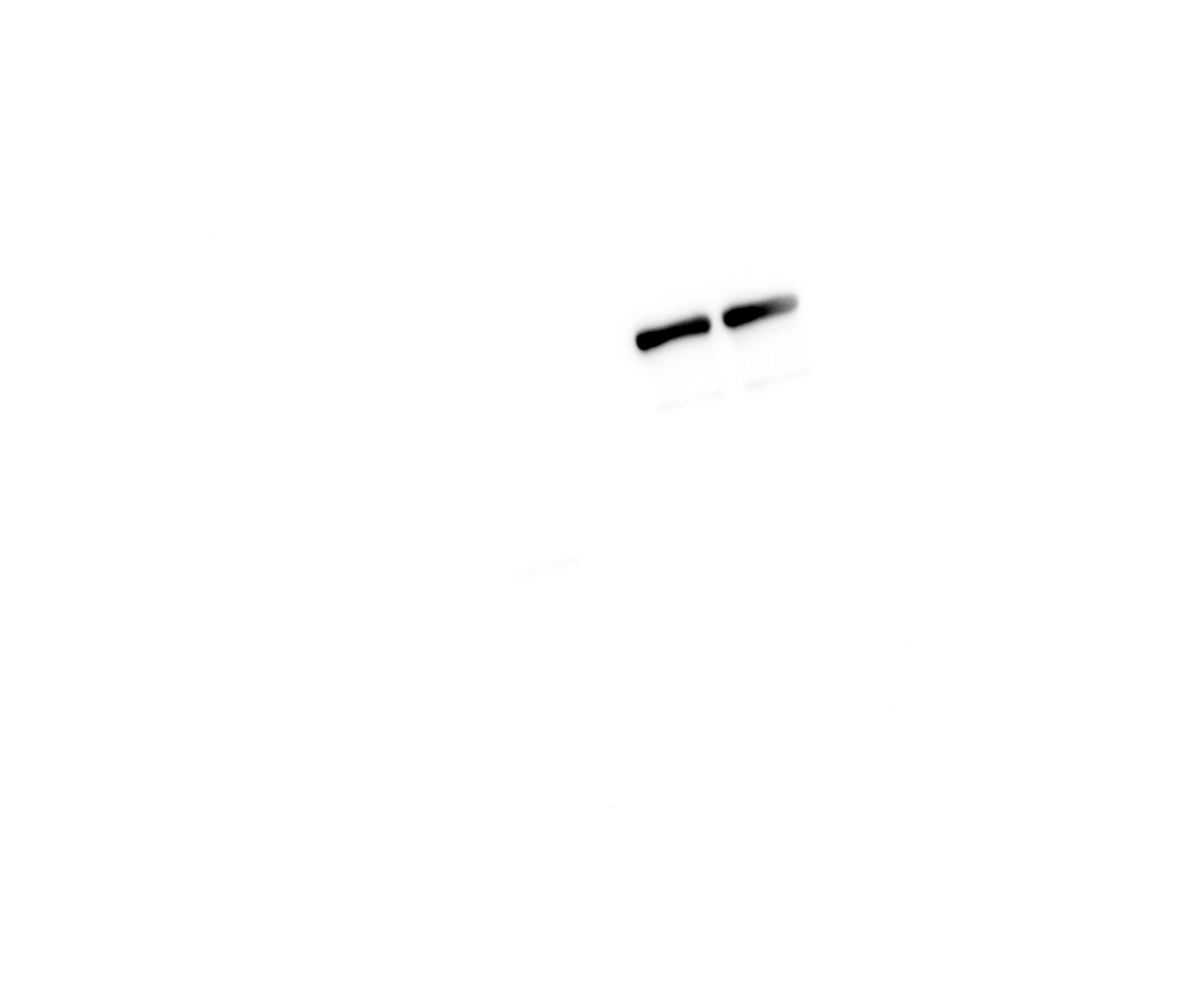

Supplement: Figure 3—source data 1. [file elife-97196-fig3-data1.zip › Figure3-SourceData1/Figure3C/fig3C a-myc.tif]

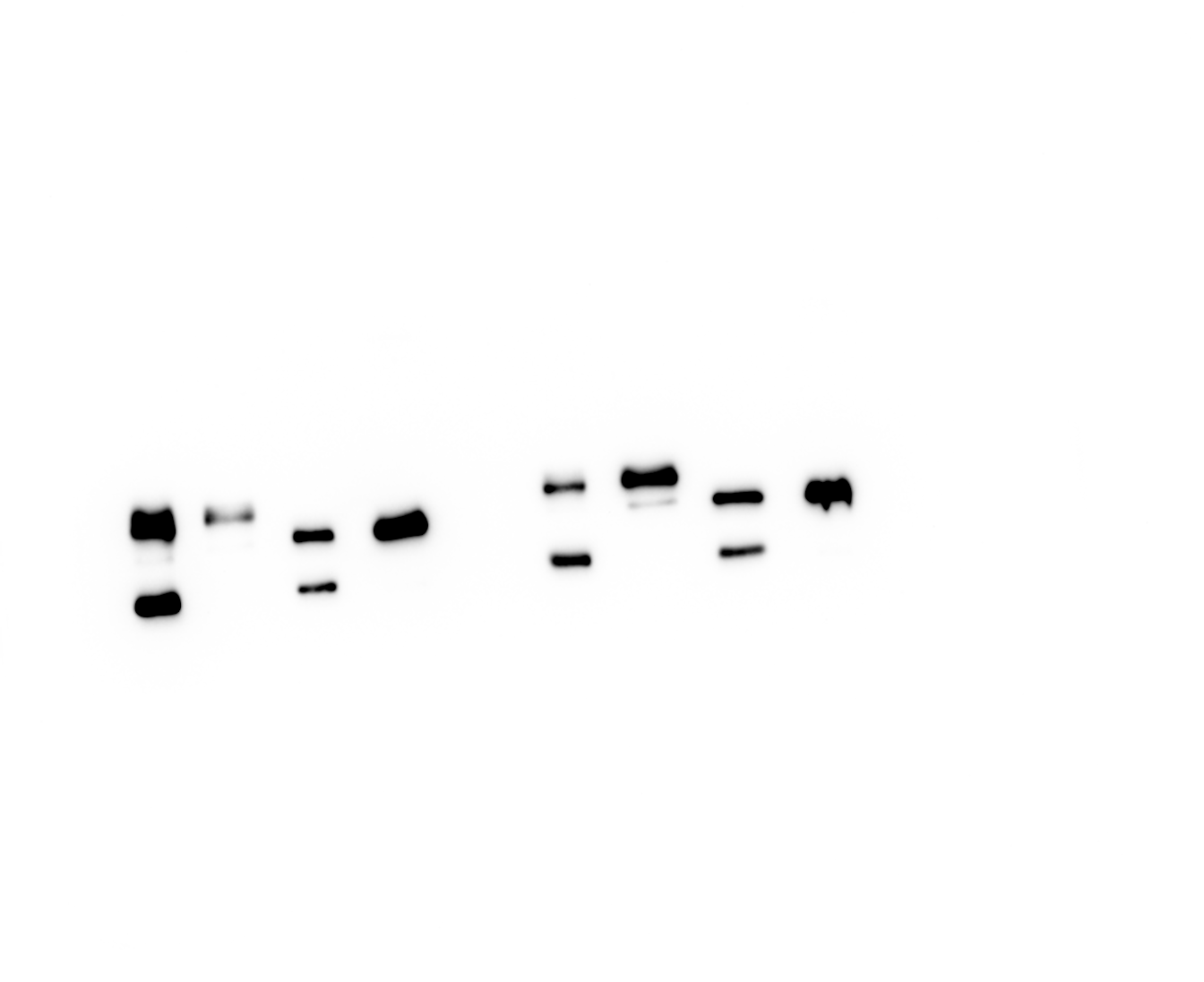

Supplement: Figure 3—source data 1. [file elife-97196-fig3-data1.zip › Figure3-SourceData1/Figure3C/fig3C A-FALG .tif]

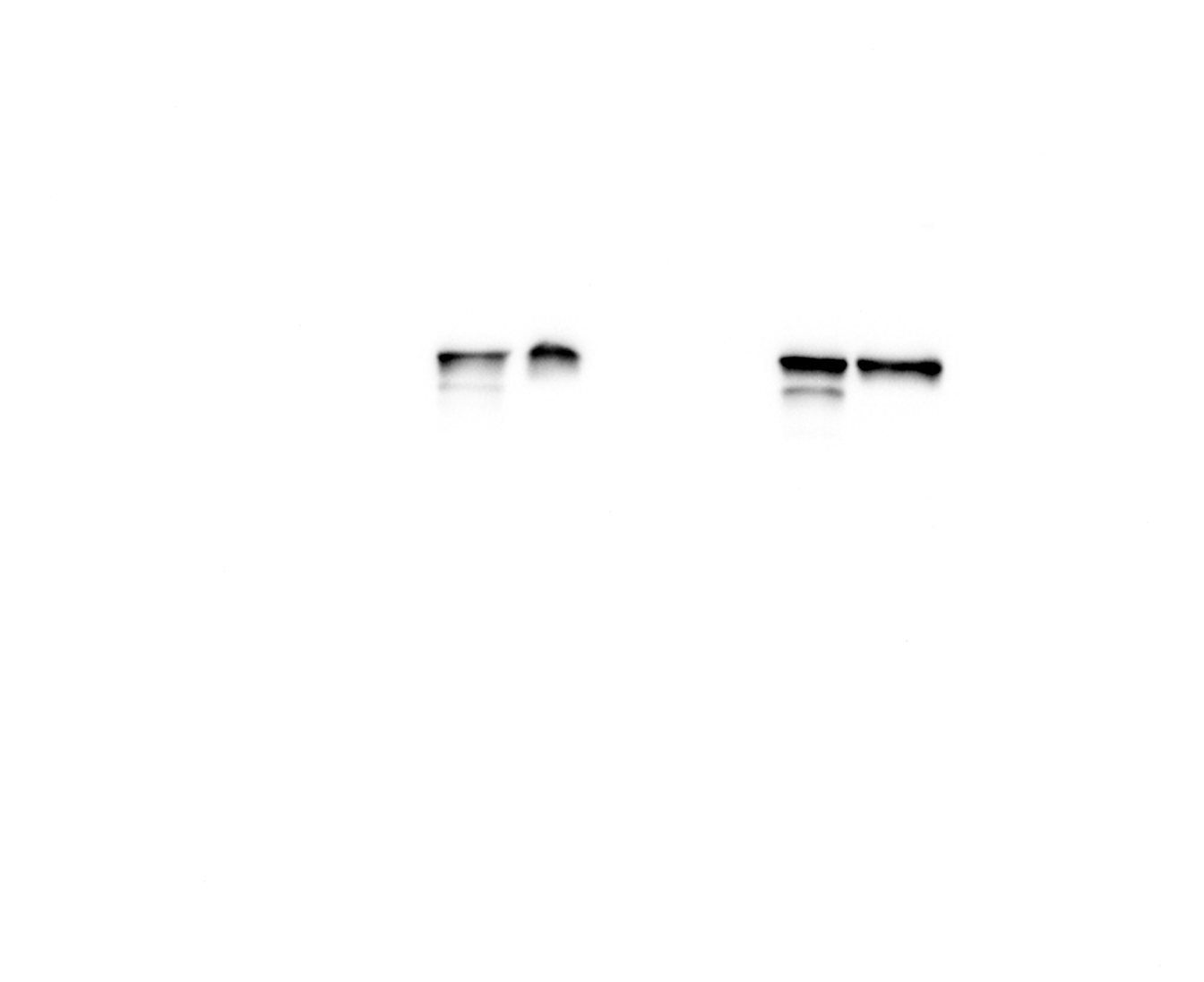

Supplement: Figure 3—source data 1. [file elife-97196-fig3-data1.zip › Figure3-SourceData1/Figure3C/fig3C a-ha .tif]

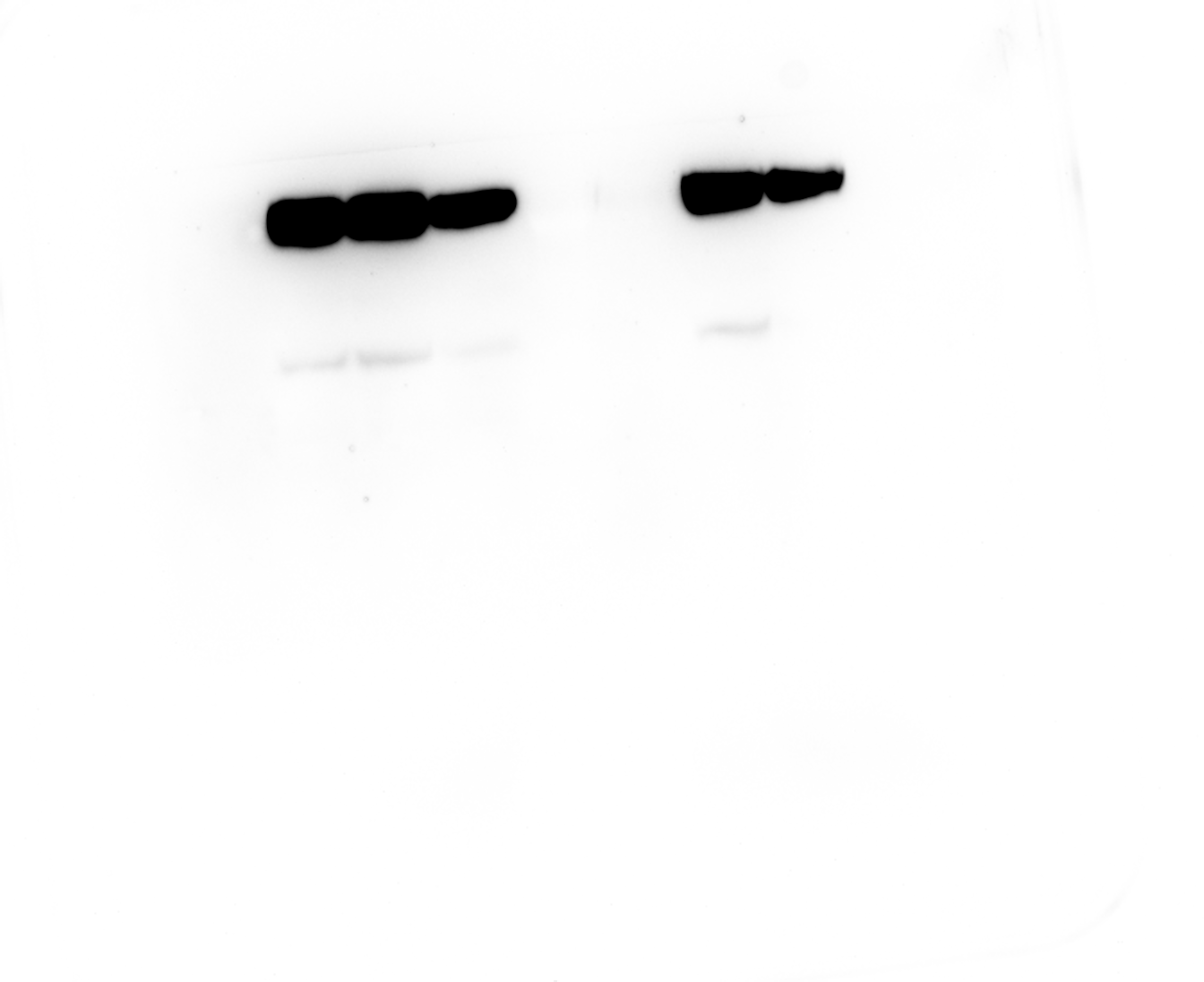

Supplement: Figure 3—source data 1. [file elife-97196-fig3-data1.zip › Figure3-SourceData1/Figure3B/fig3B a-myc.tif]

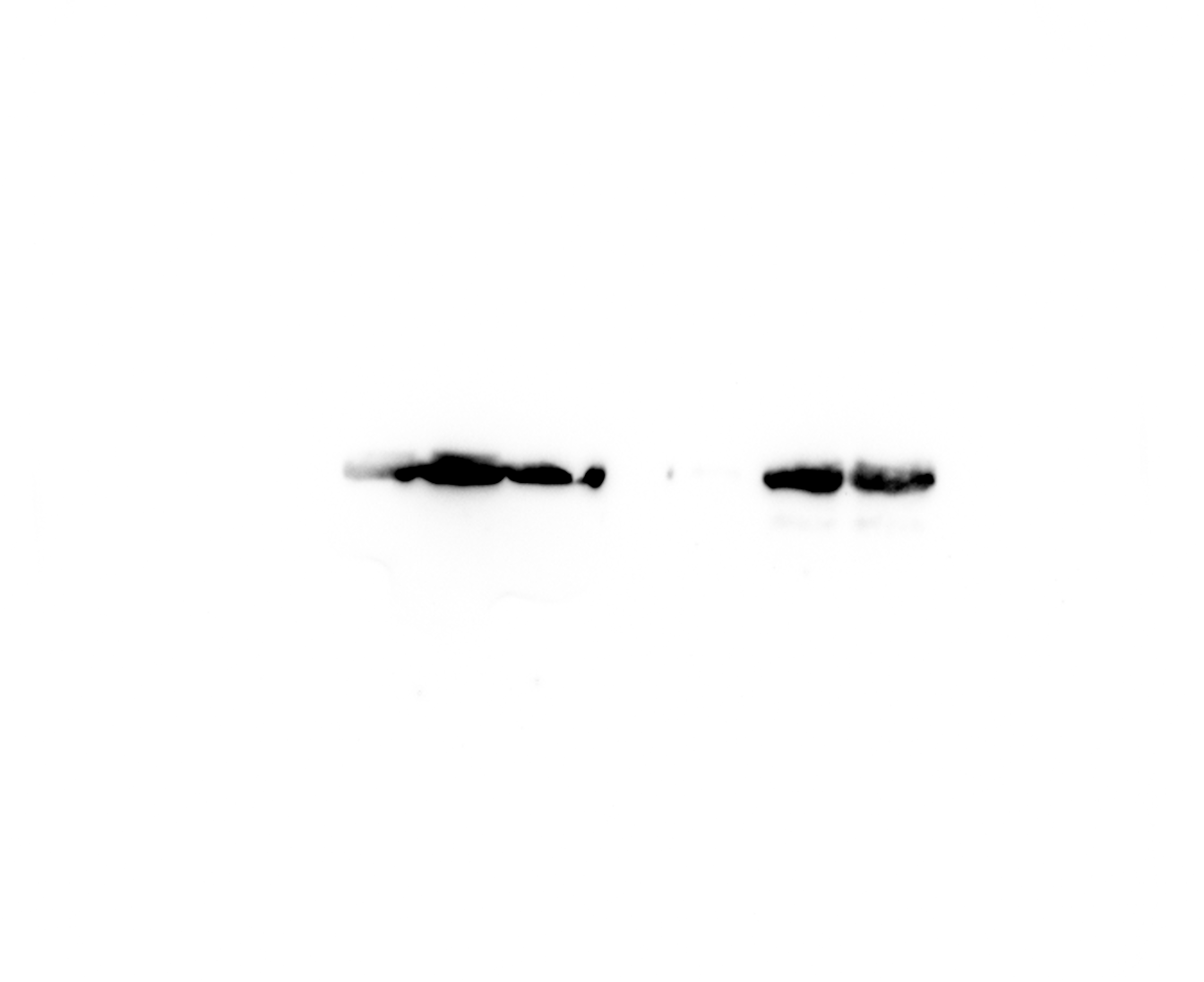

Supplement: Figure 3—source data 1. [file elife-97196-fig3-data1.zip › Figure3-SourceData1/Figure3B/fig3B a-actin.tif]

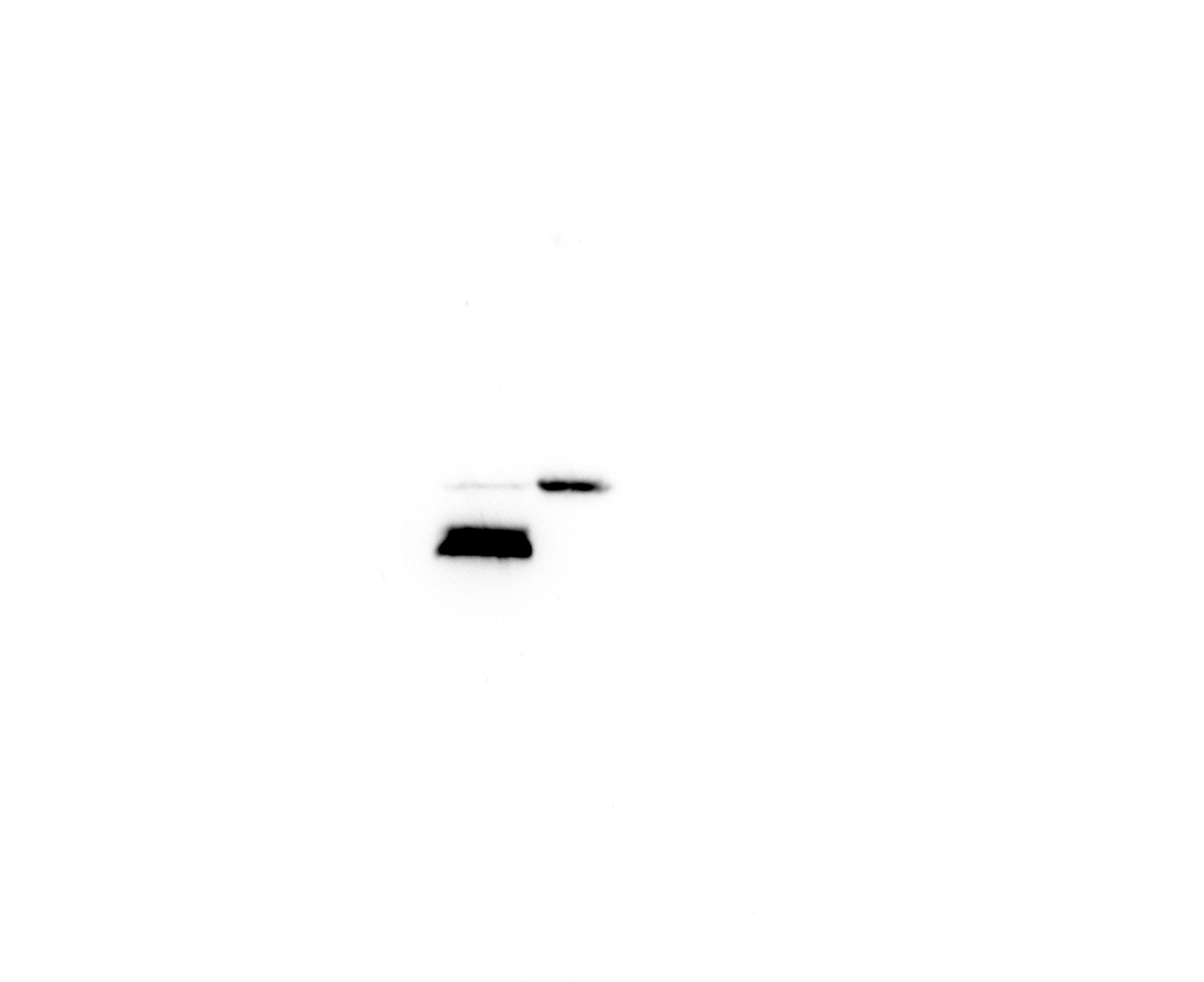

Supplement: Figure 3—source data 1. [file elife-97196-fig3-data1.zip › Figure3-SourceData1/Figure3B/fig3b a-flag.tif]

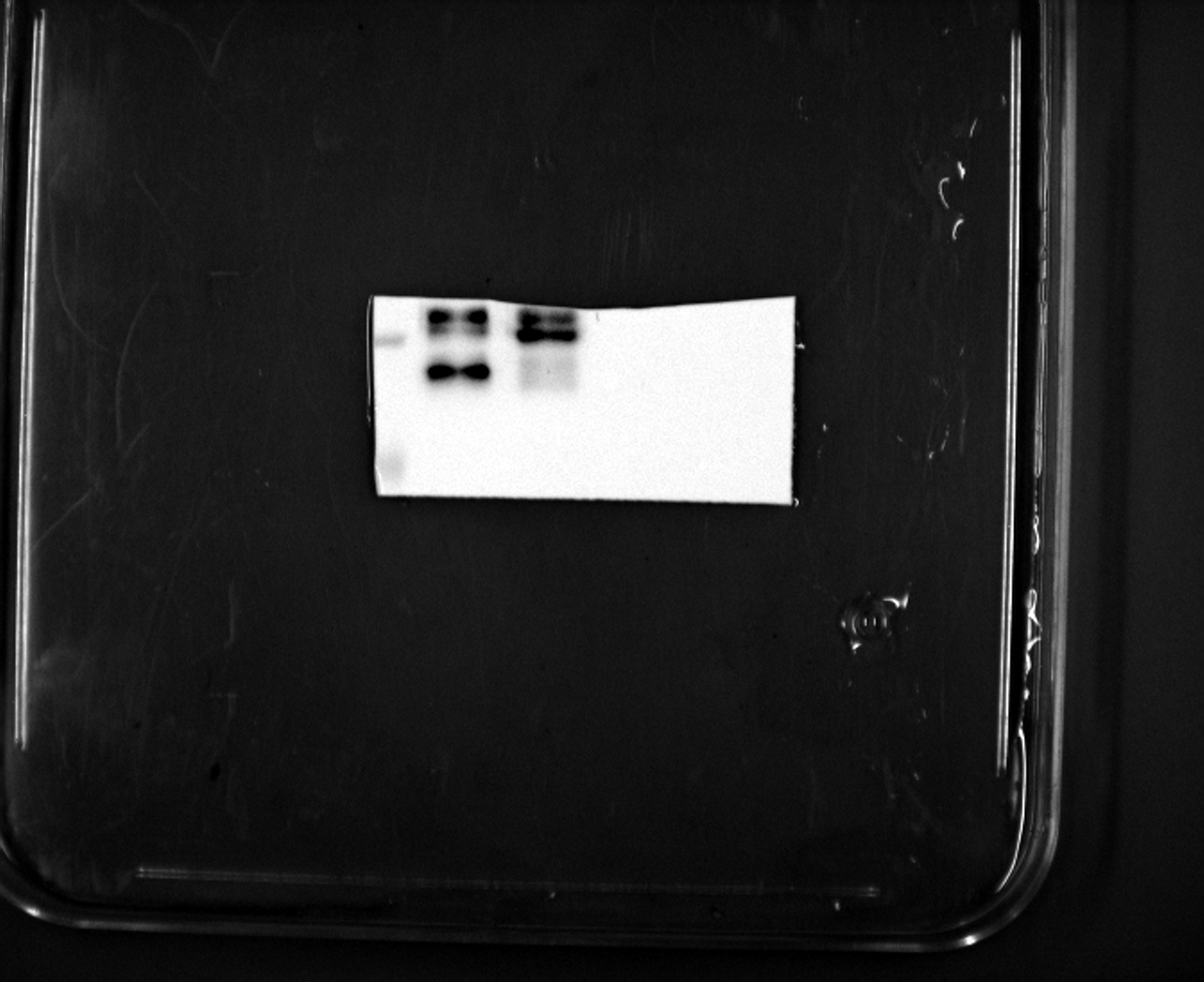

Supplement: Figure 3—source data 1. [file elife-97196-fig3-data1.zip › Figure3-SourceData1/Figure3E/fig3E a-gfp FT.tif]

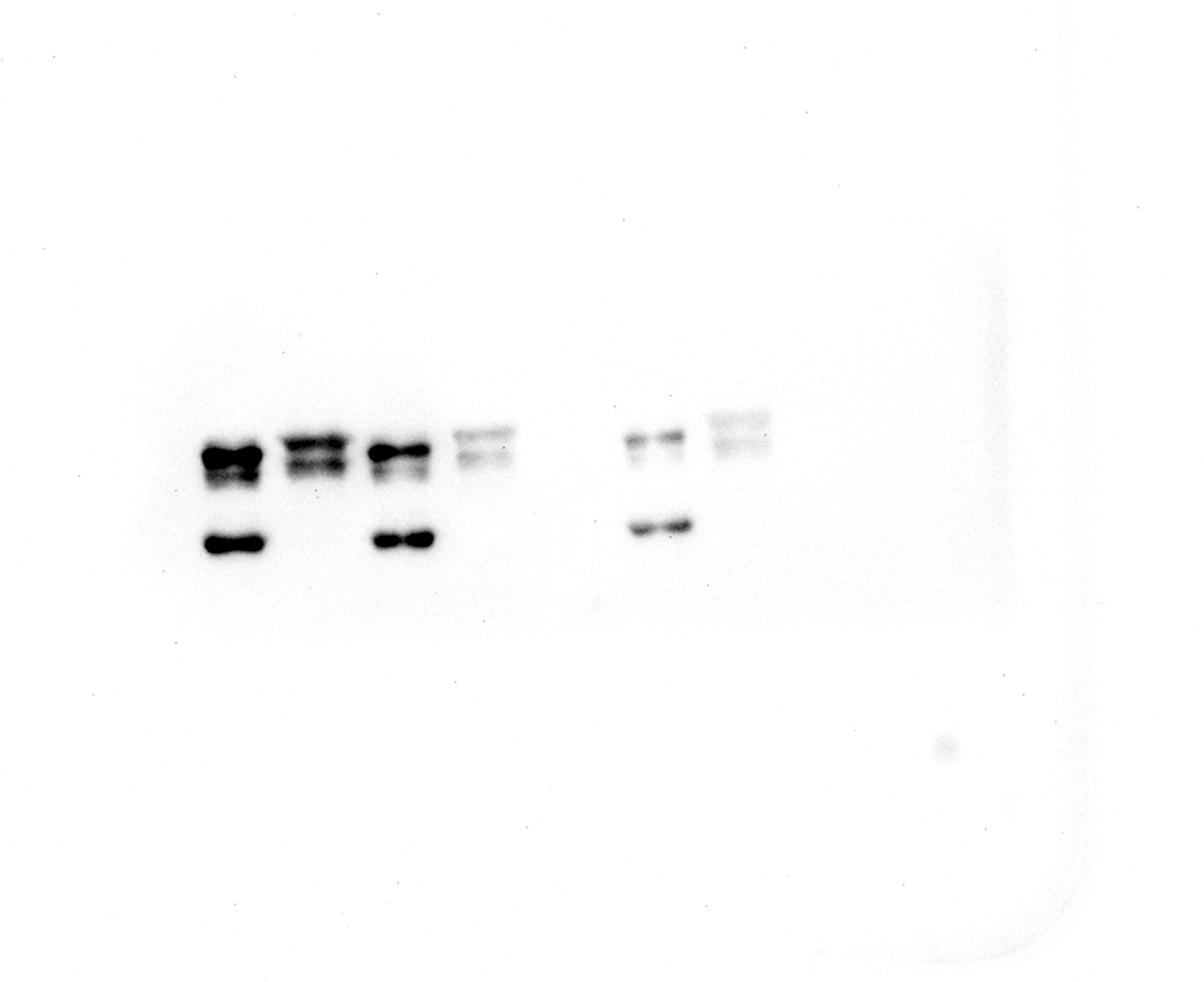

Supplement: Figure 3—source data 1. [file elife-97196-fig3-data1.zip › Figure3-SourceData1/Figure3E/fig3E a- flag .tif]

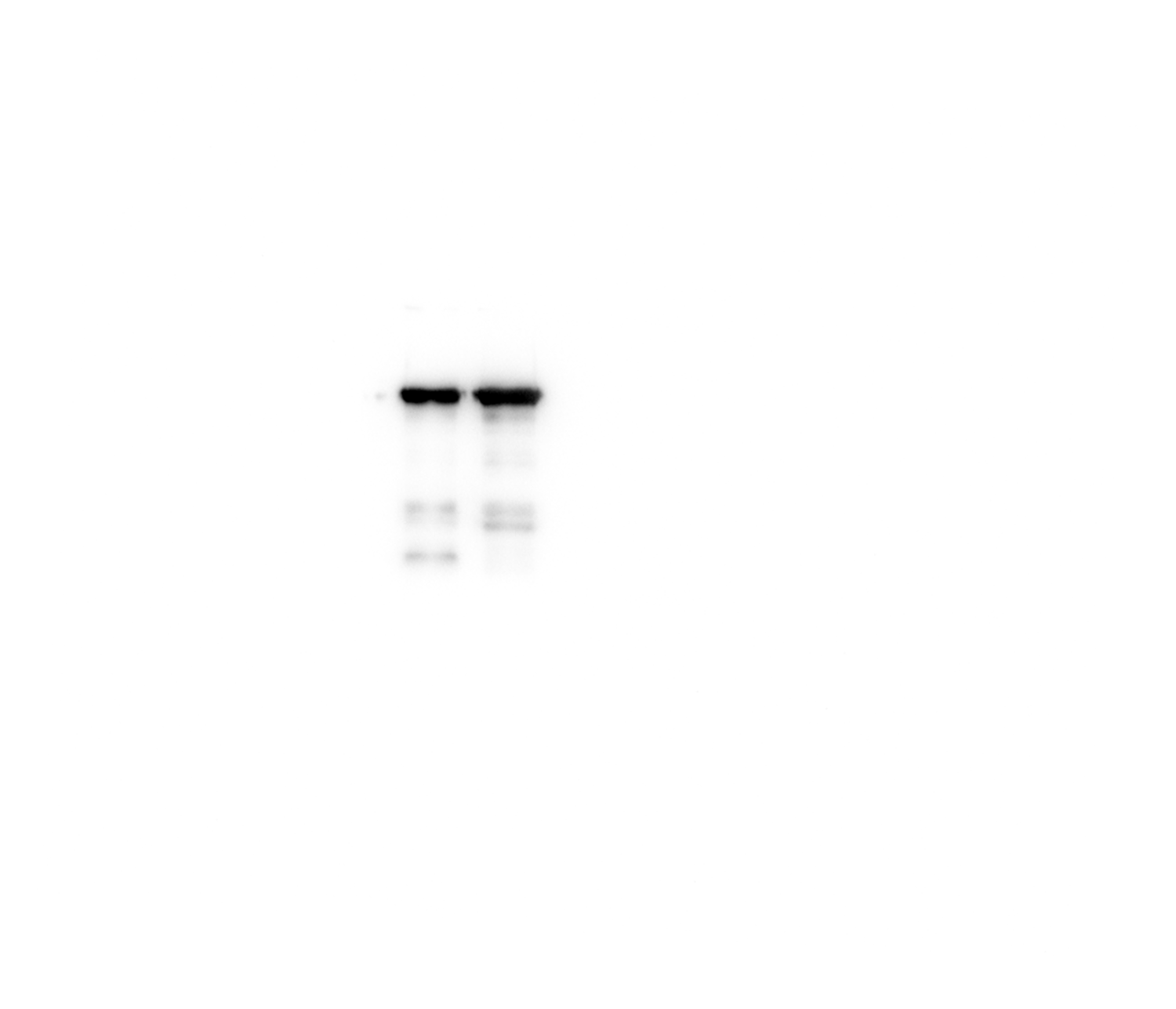

Supplement: Figure 3—source data 1. [file elife-97196-fig3-data1.zip › Figure3-SourceData1/Figure3E/a-gfp FULL.tif]

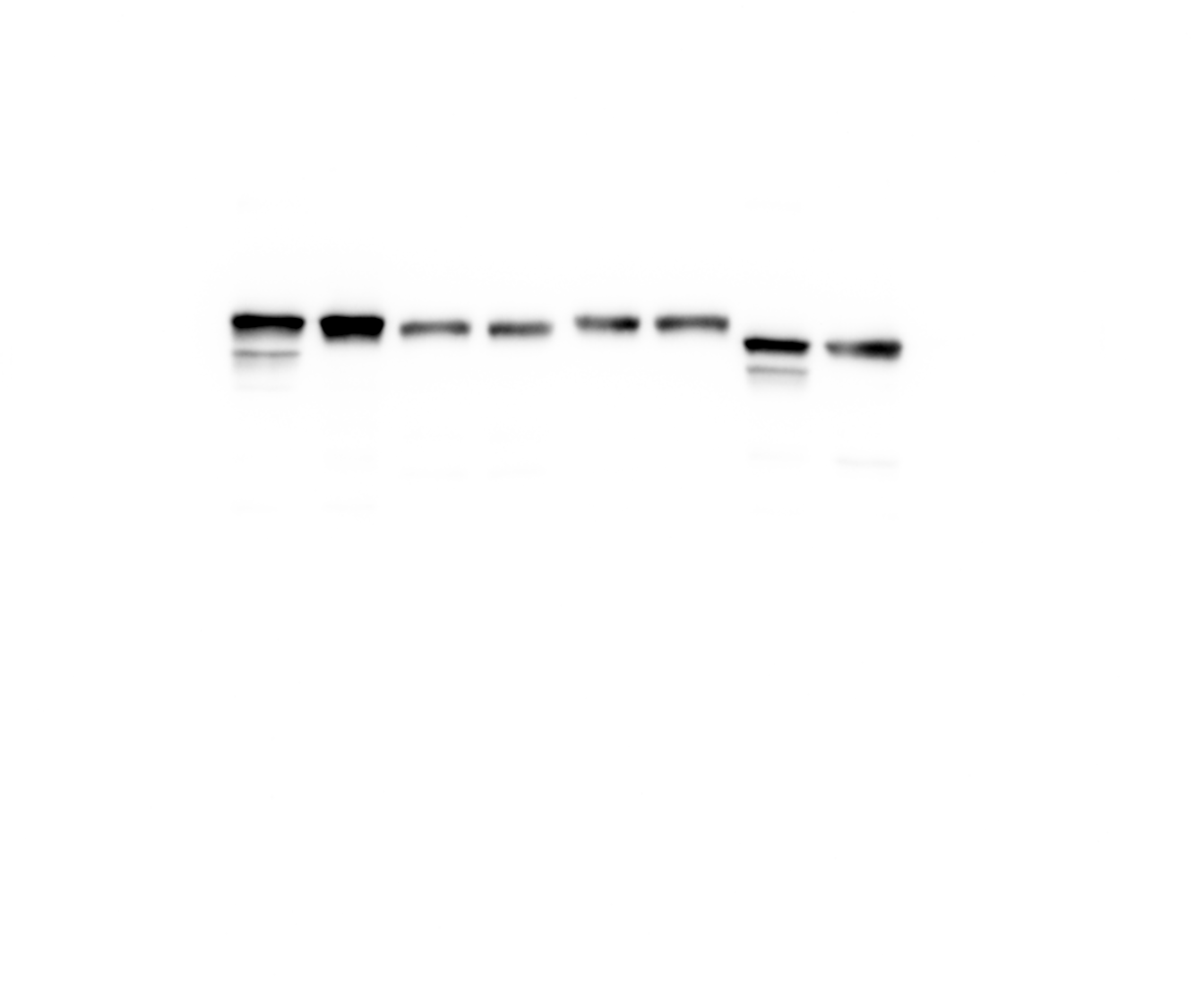

Supplement: Figure 3—source data 1. [file elife-97196-fig3-data1.zip › Figure3-SourceData1/Figure3G/FIG3G a- ha.tif]

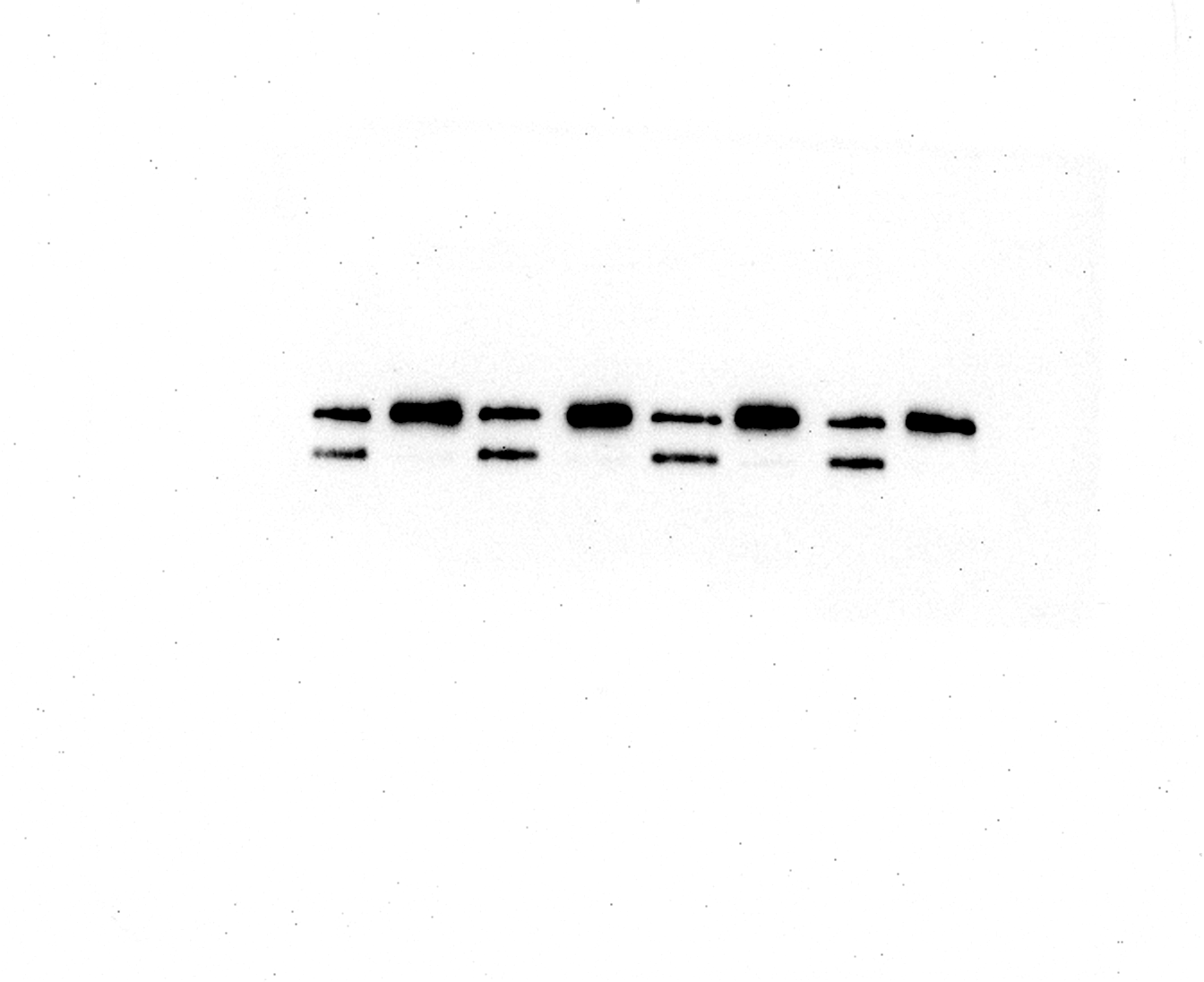

Supplement: Figure 3—source data 1. [file elife-97196-fig3-data1.zip › Figure3-SourceData1/Figure3G/FIG3G a-flag.tif]

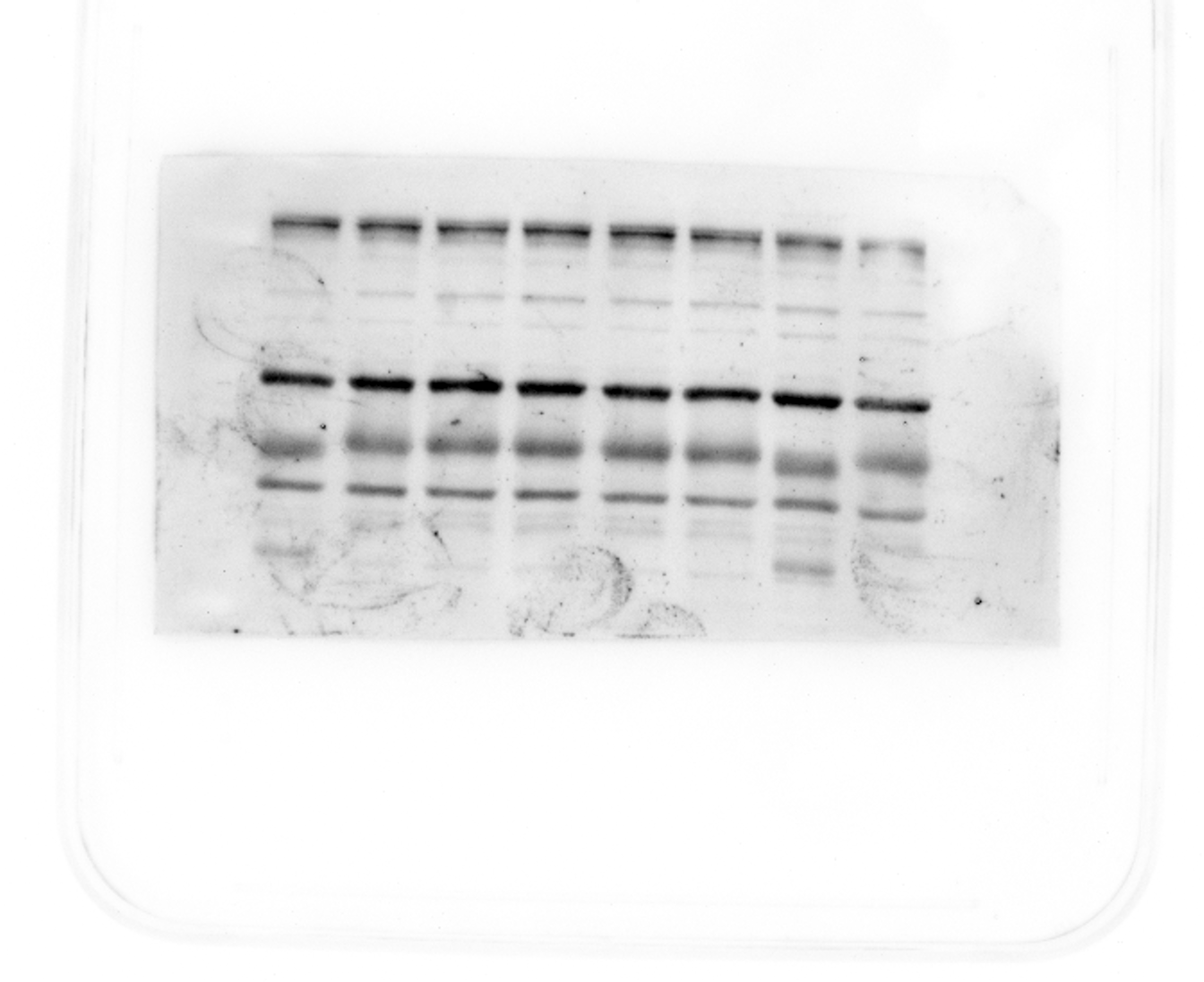

Supplement: Figure 3—source data 1. [file elife-97196-fig3-data1.zip › Figure3-SourceData1/Figure3G/FIG3G a-groel .tif]

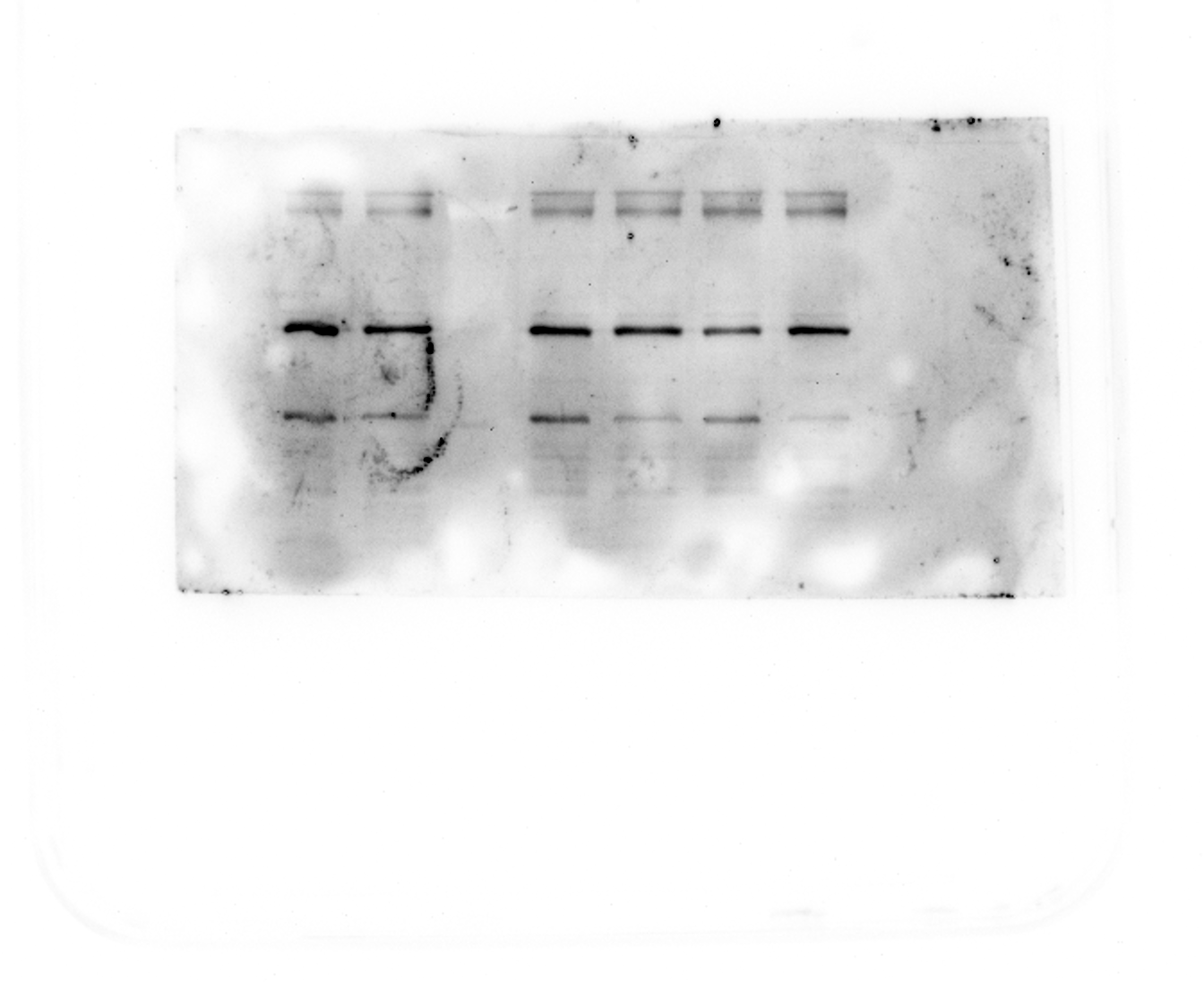

Supplement: Figure 3—source data 1. [file elife-97196-fig3-data1.zip › Figure3-SourceData1/Figure3F/FIG3F a-groel .tif]

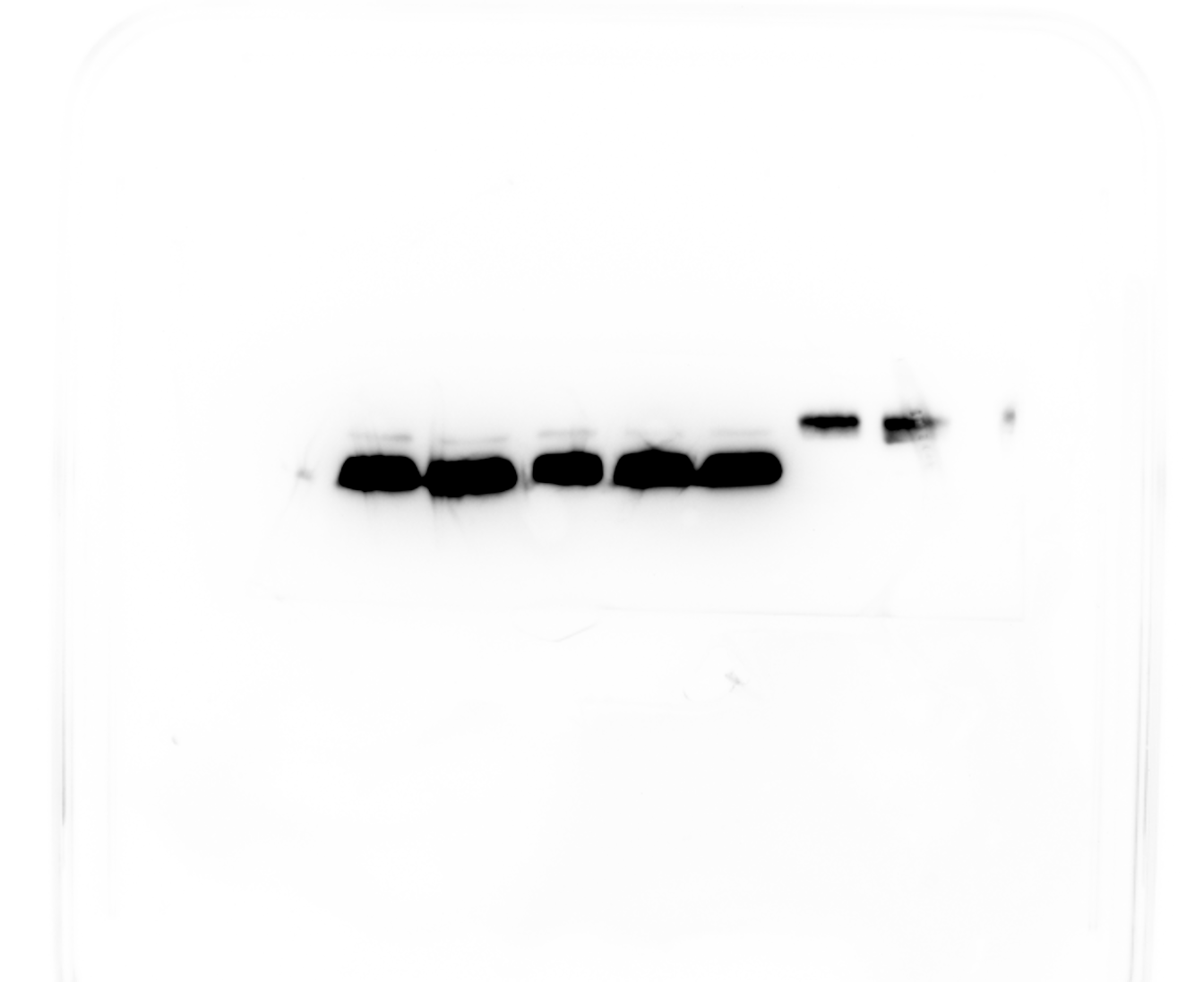

Supplement: Figure 3—source data 1. [file elife-97196-fig3-data1.zip › Figure3-SourceData1/Figure3A/fig3A a-strerp 79 nb.tif]

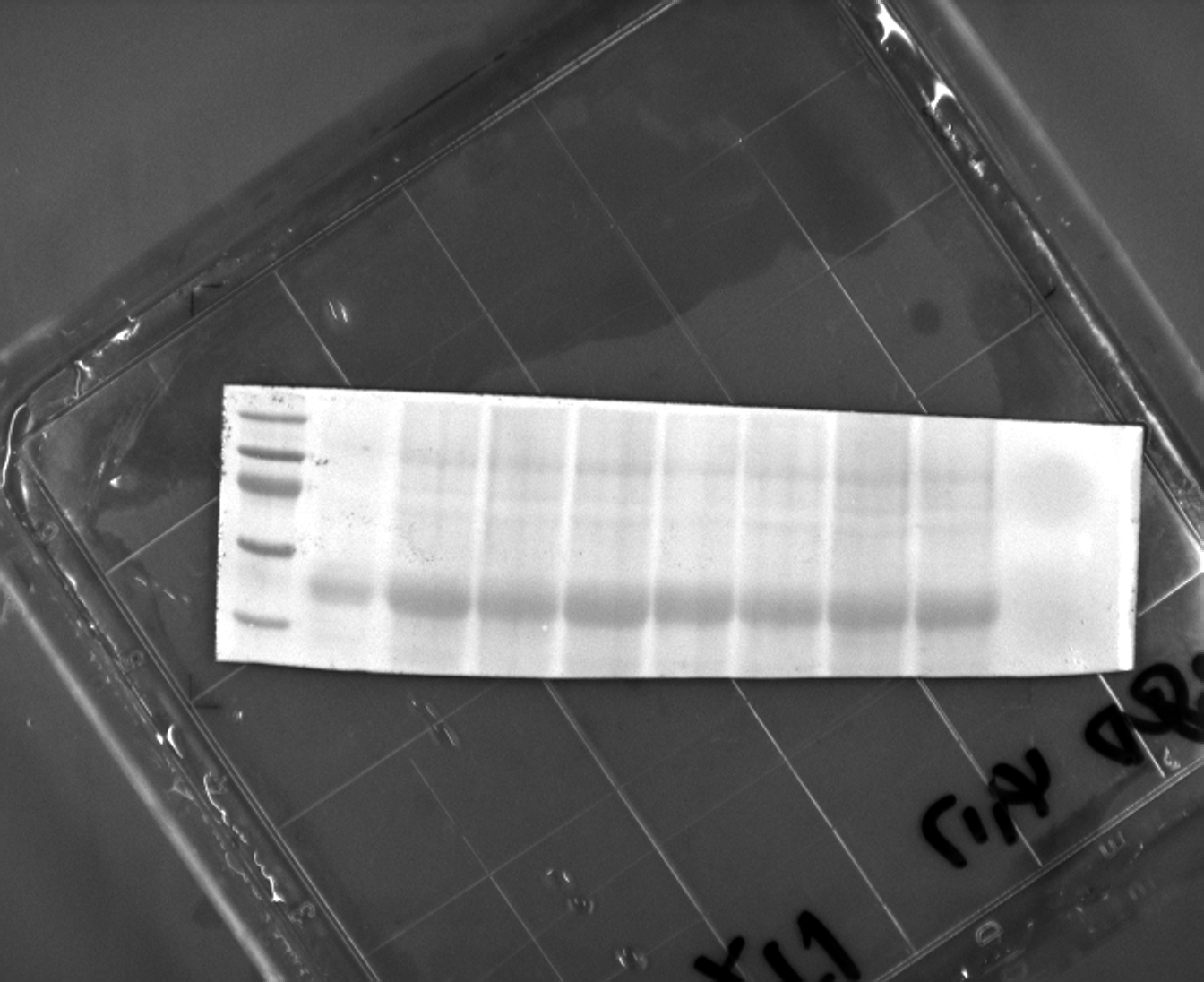

Supplement: Figure 3—source data 1. [file elife-97196-fig3-data1.zip › Figure3-SourceData1/Figure3A/fig3A Ponceau S rb.tif]

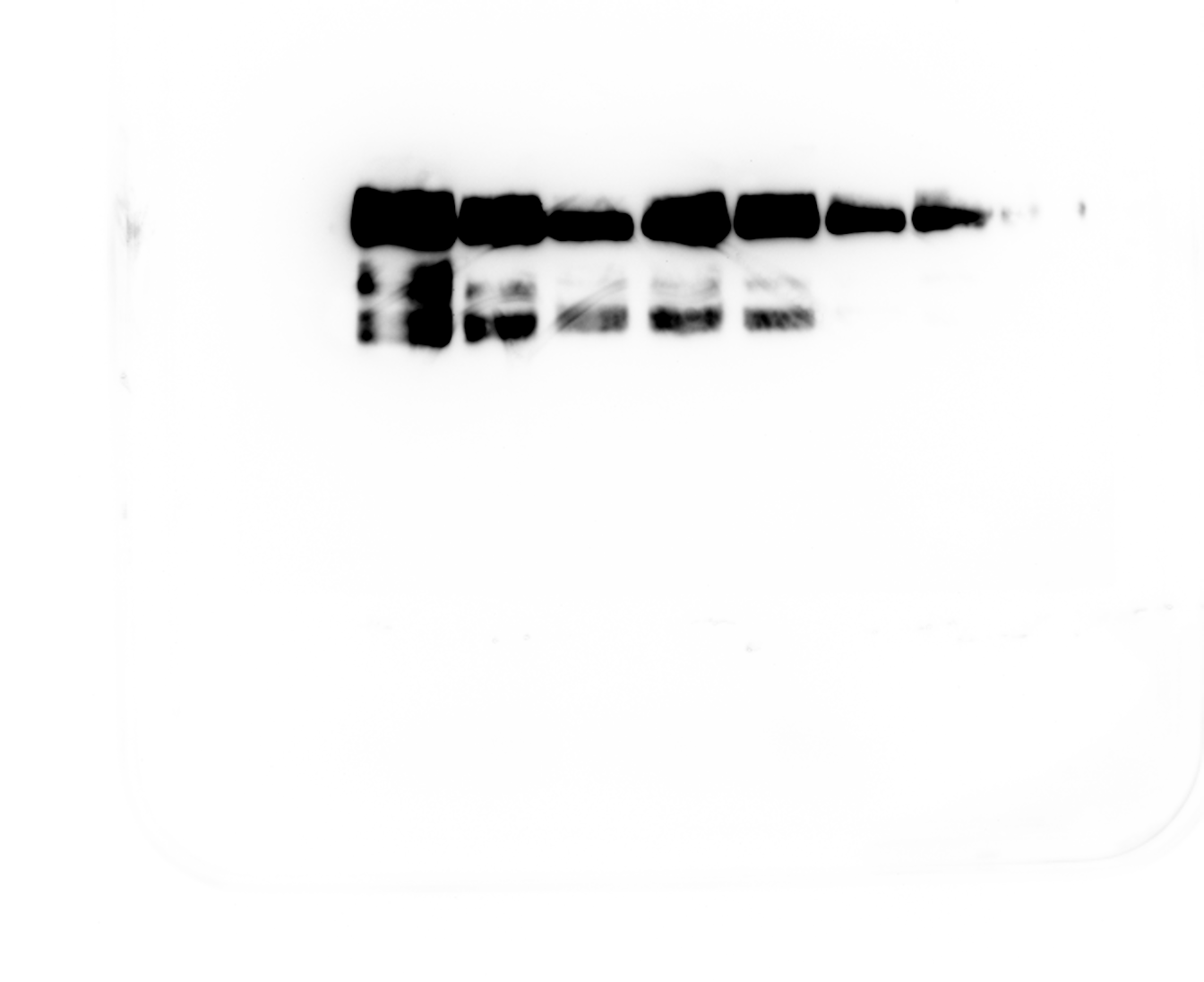

Supplement: Figure 3—source data 1. [file elife-97196-fig3-data1.zip › Figure3-SourceData1/Figure3A/fig3A a-gfp .tif]

Fig. 3G

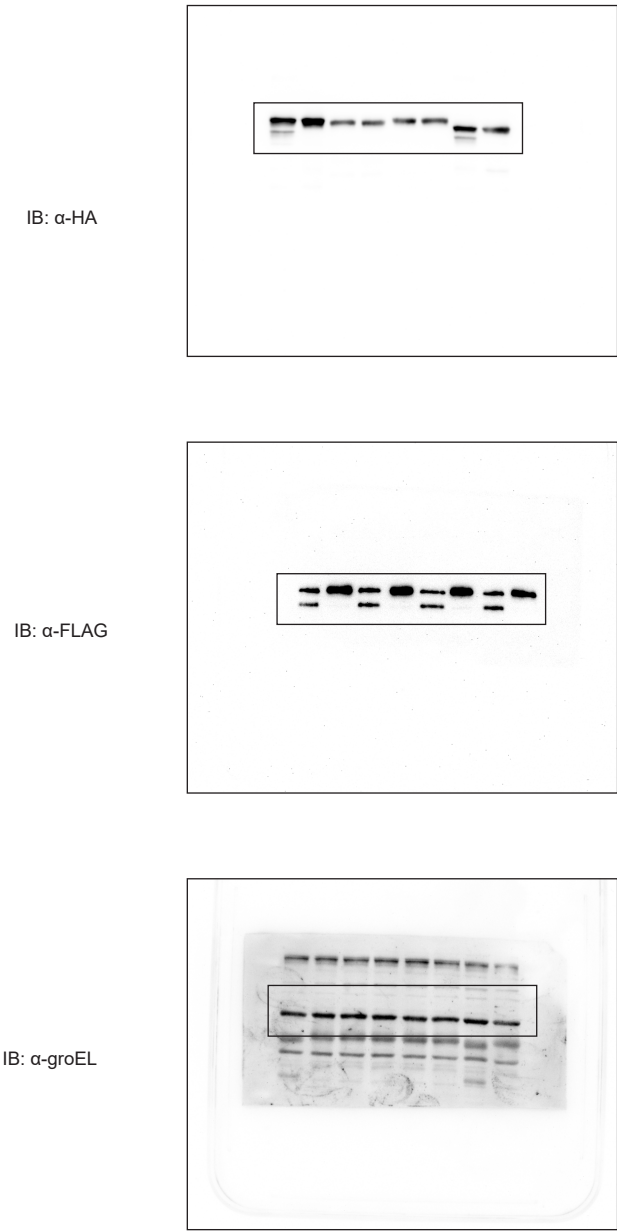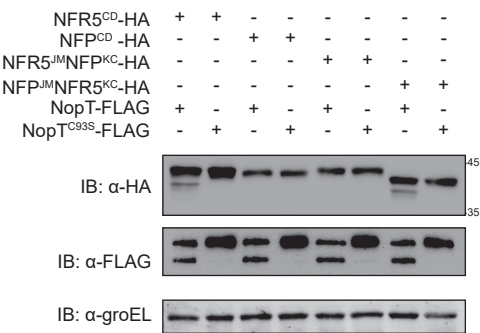

Supplement: Figure 3—source data 2. [file elife-97196-fig3-data2.zip › Figure3-SourceData2/Figure3G.pdf]

Fig. 3F

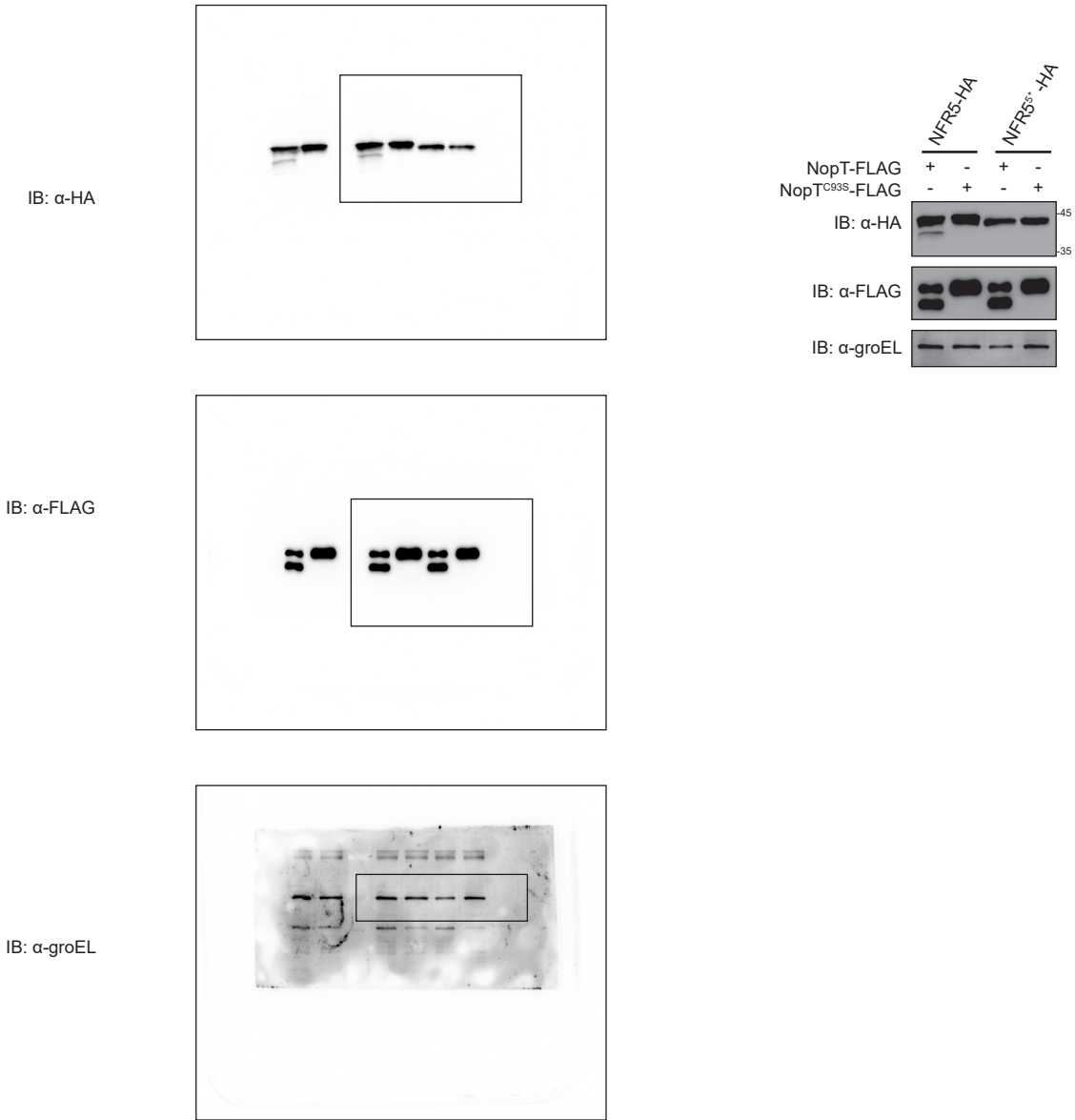

Supplement: Figure 3—source data 2. [file elife-97196-fig3-data2.zip › Figure3-SourceData2/Figure3F.pdf]

Fig. 3D

IB:  $\alpha$ -HA

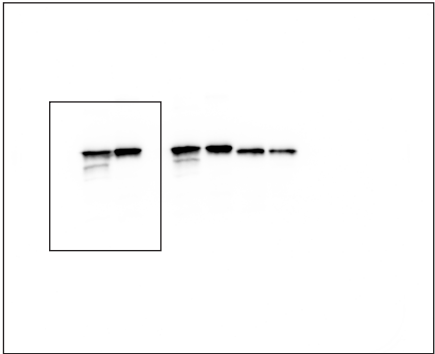

IB:  $\alpha$ -FLAG

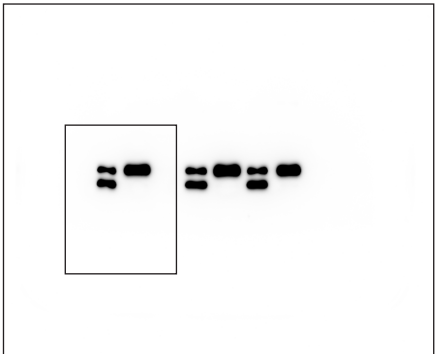

IB:  $\alpha$ -groEL

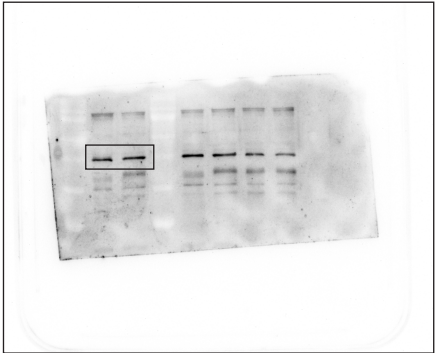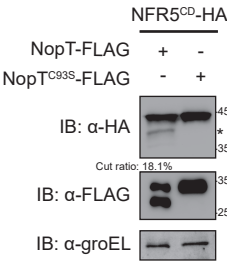

Supplement: Figure 3—source data 2. [file elife-97196-fig3-data2.zip › Figure3-SourceData2/Figure3D.pdf]

Fig. 3E

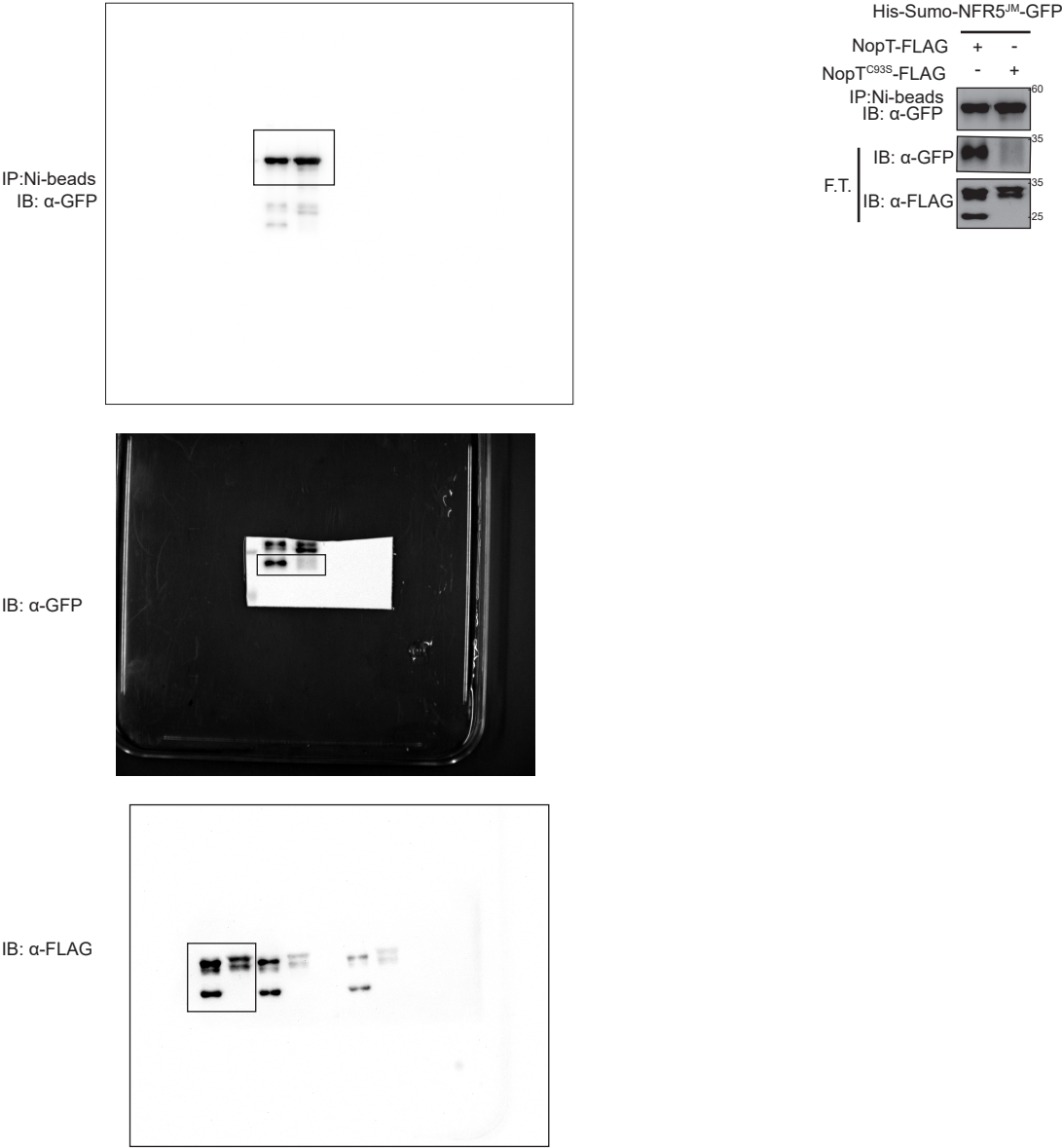

Supplement: Figure 3—source data 2. [file elife-97196-fig3-data2.zip › Figure3-SourceData2/Figure3E.pdf]

Fig. 3A

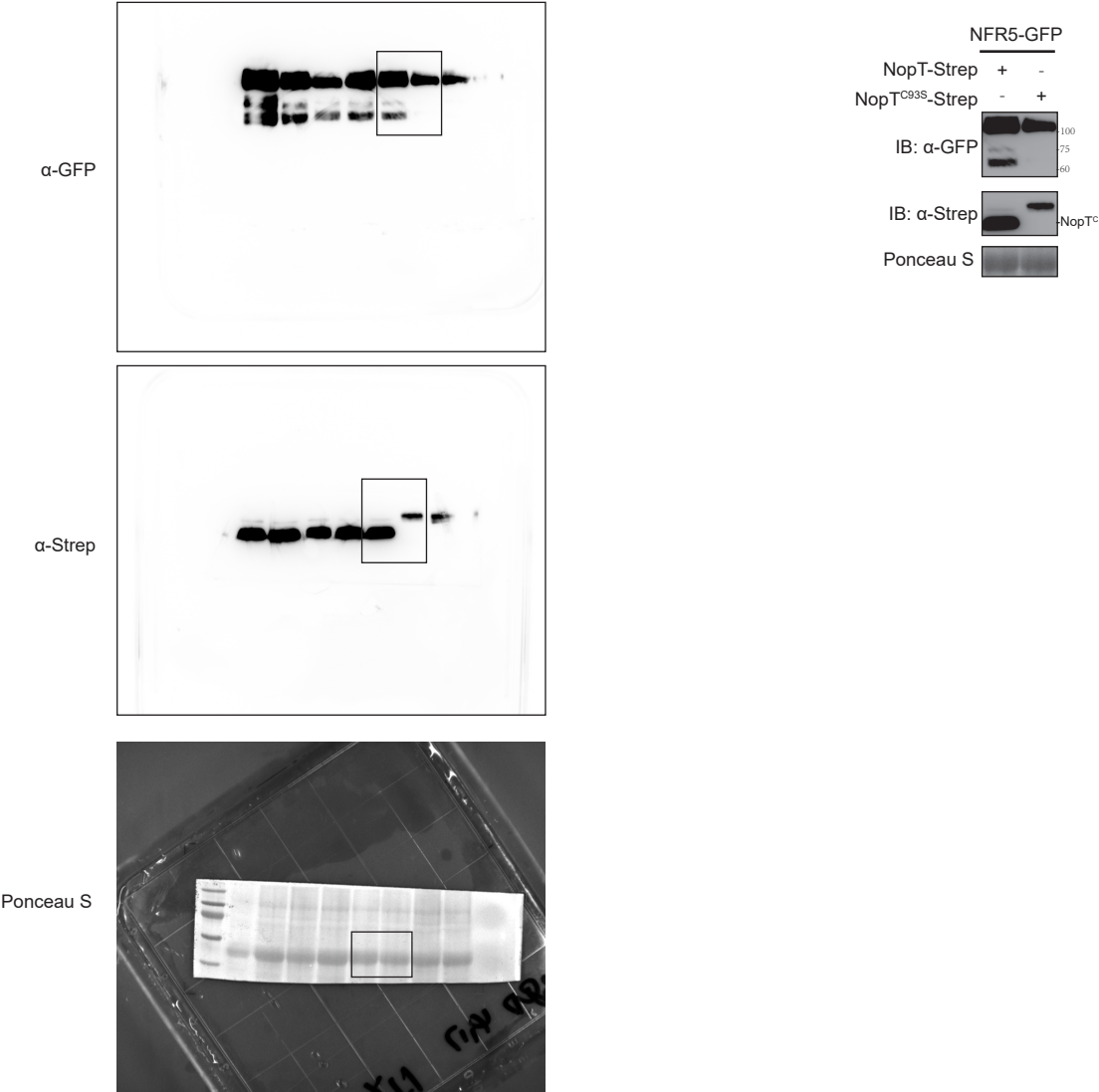

Supplement: Figure 3—source data 2. [file elife-97196-fig3-data2.zip › Figure3-SourceData2/Figure3A.pdf]

Fig. 3B

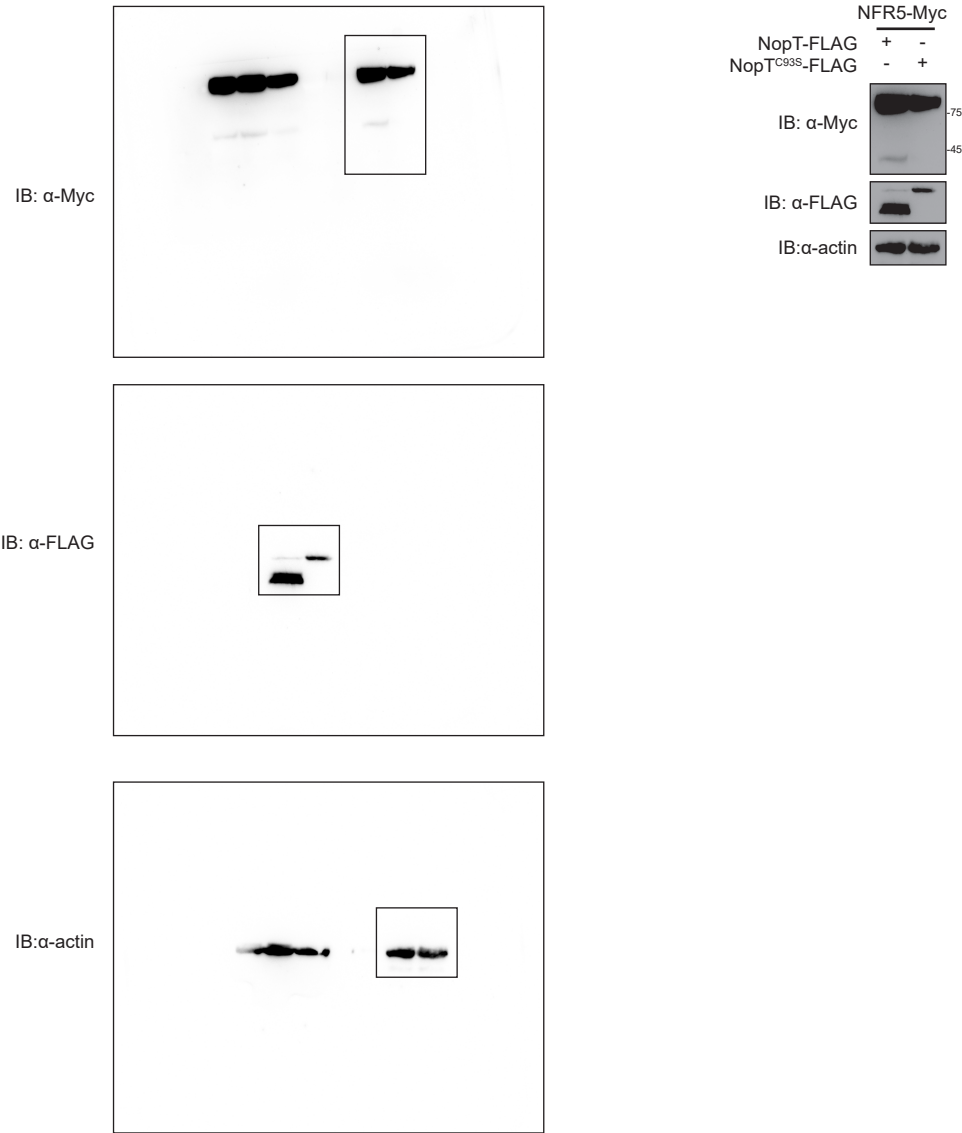

Supplement: Figure 3—source data 2. [file elife-97196-fig3-data2.zip › Figure3-SourceData2/Figure3B.pdf]

Fig. 3C

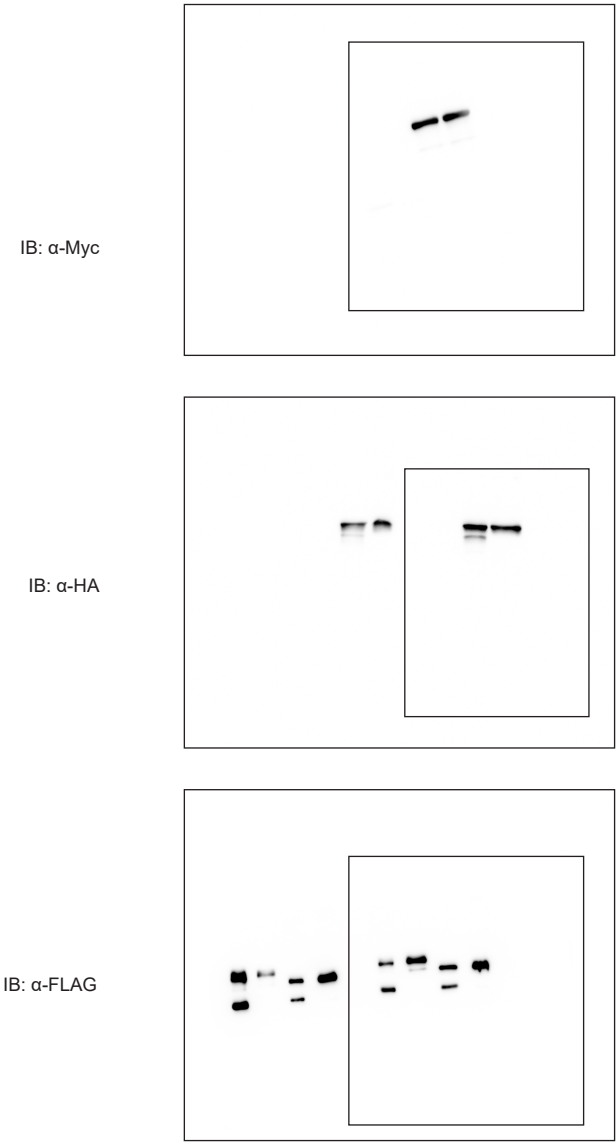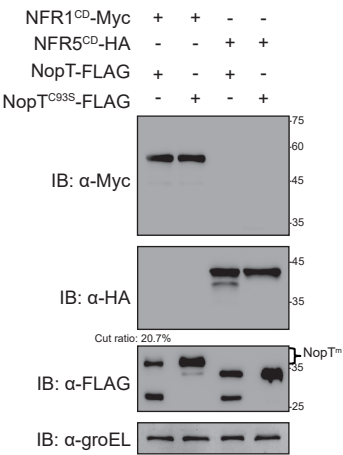

Supplement: Figure 3—source data 2. [file elife-97196-fig3-data2.zip › Figure3-SourceData2/Figure3C.pdf]

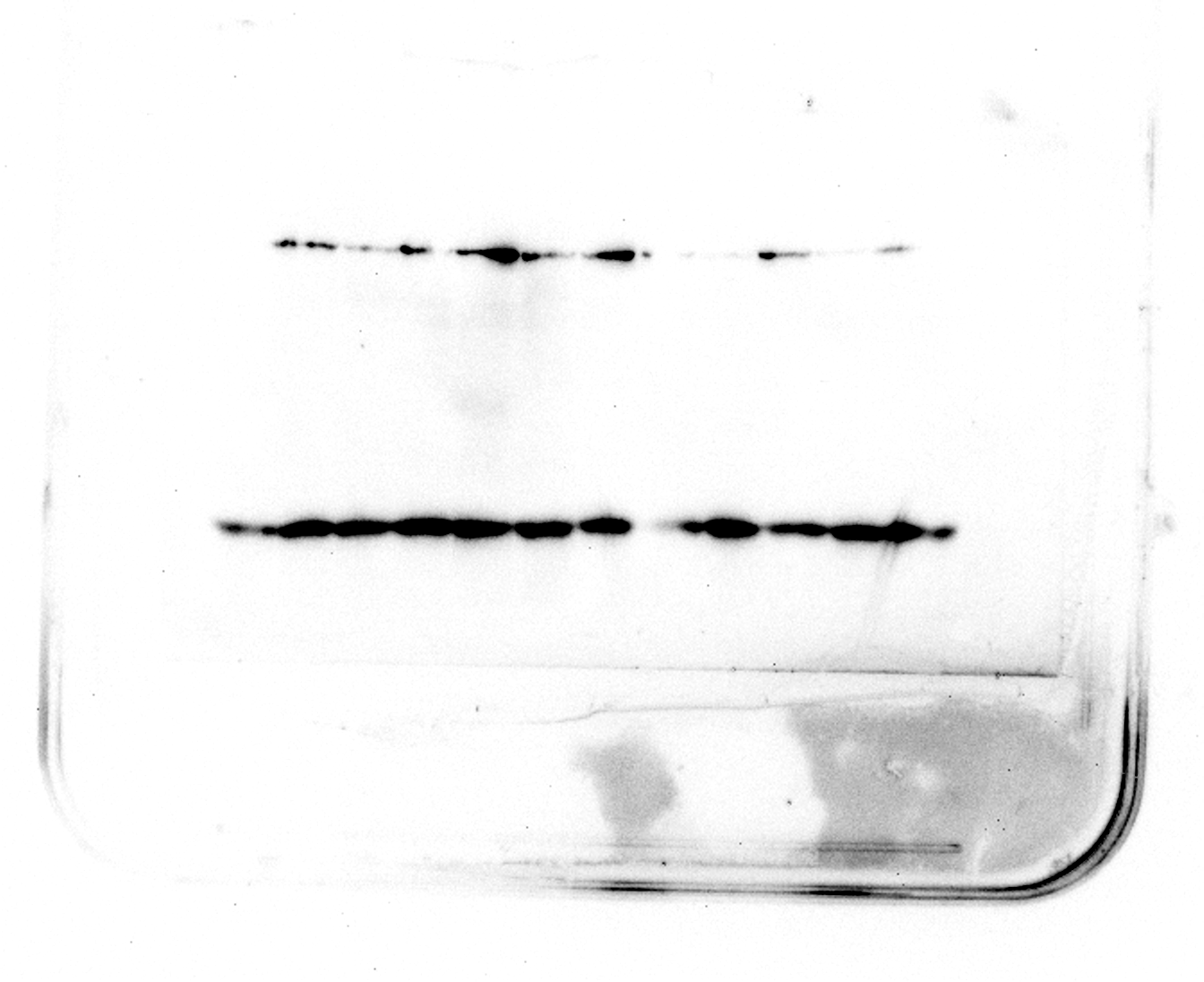

Supplement: Figure 3—figure supplement 1—source data 1. [file elife-97196-fig3-figsupp1-data1.zip › Figure3-S1-SourceData1/Figure3-S1A/Figure 3-figure supplement 1A A-GFP.tif]

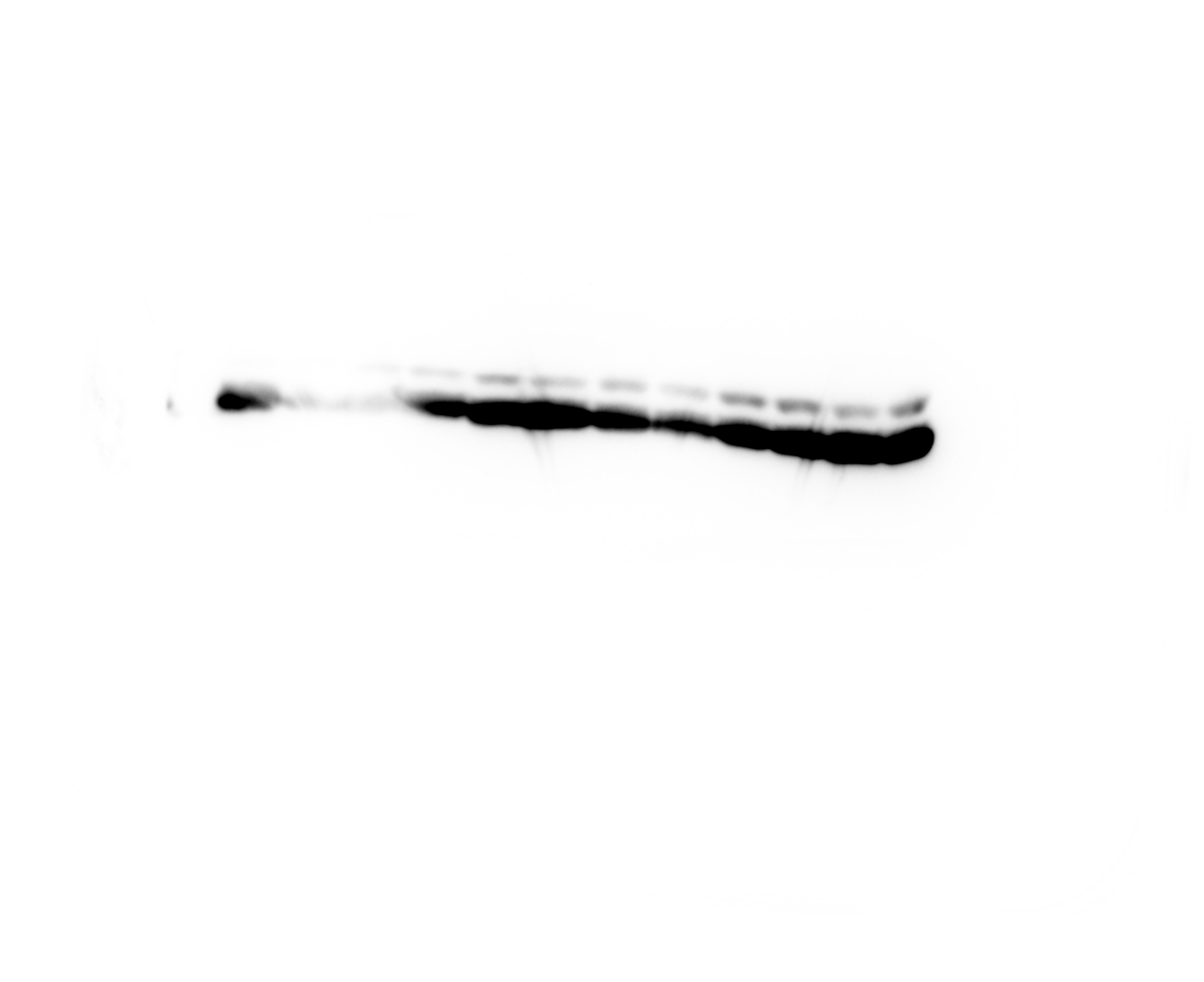

Supplement: Figure 3—figure supplement 1—source data 1. [file elife-97196-fig3-figsupp1-data1.zip › Figure3-S1-SourceData1/Figure3-S1A/Figure 3-figure supplement 1A A-STREP .tif]

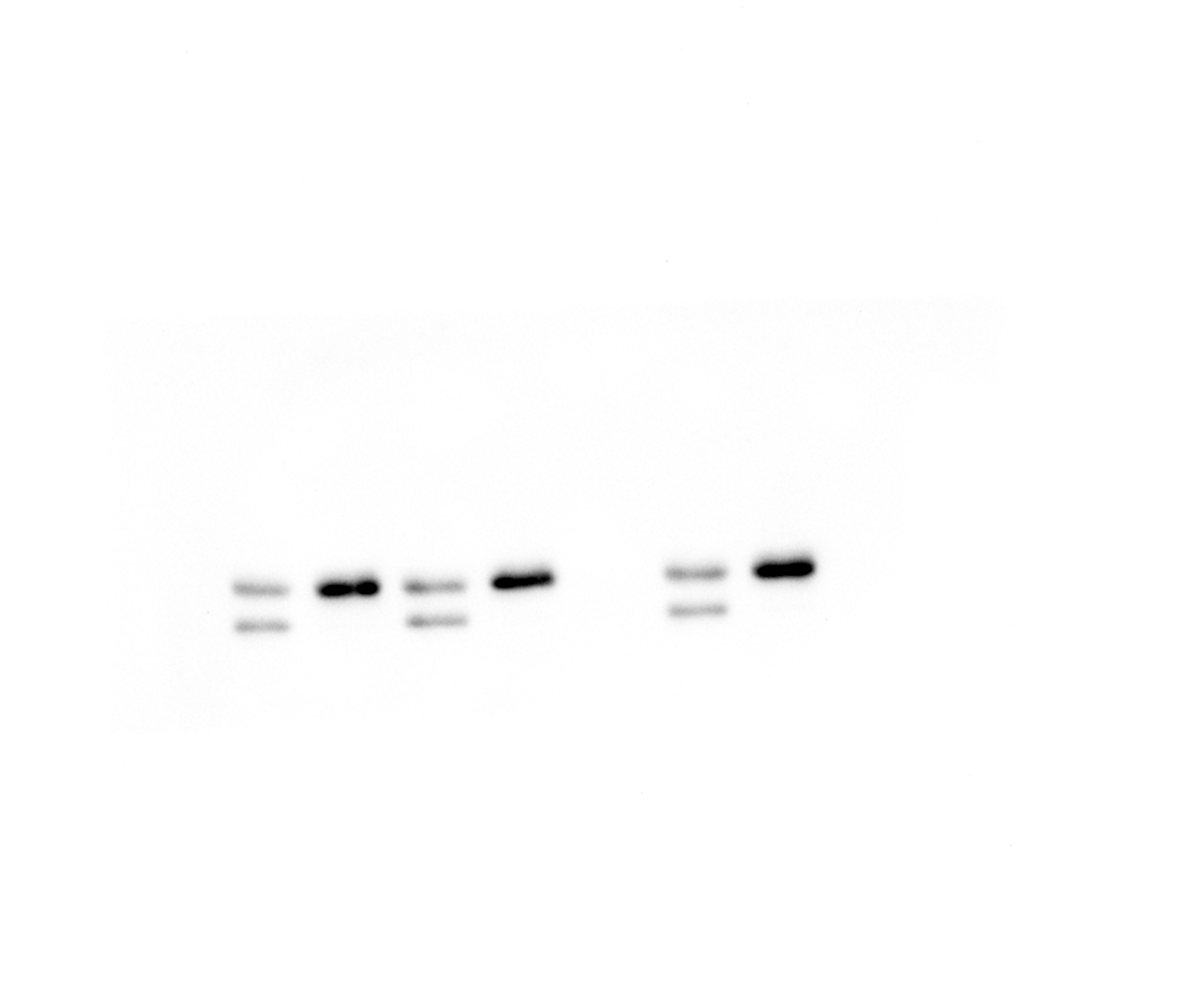

Supplement: Figure 3—figure supplement 1—source data 1. [file elife-97196-fig3-figsupp1-data1.zip › Figure3-S1-SourceData1/Figure3-S1B/Figure 3-figure supplement 1B a- FLAG.tif]

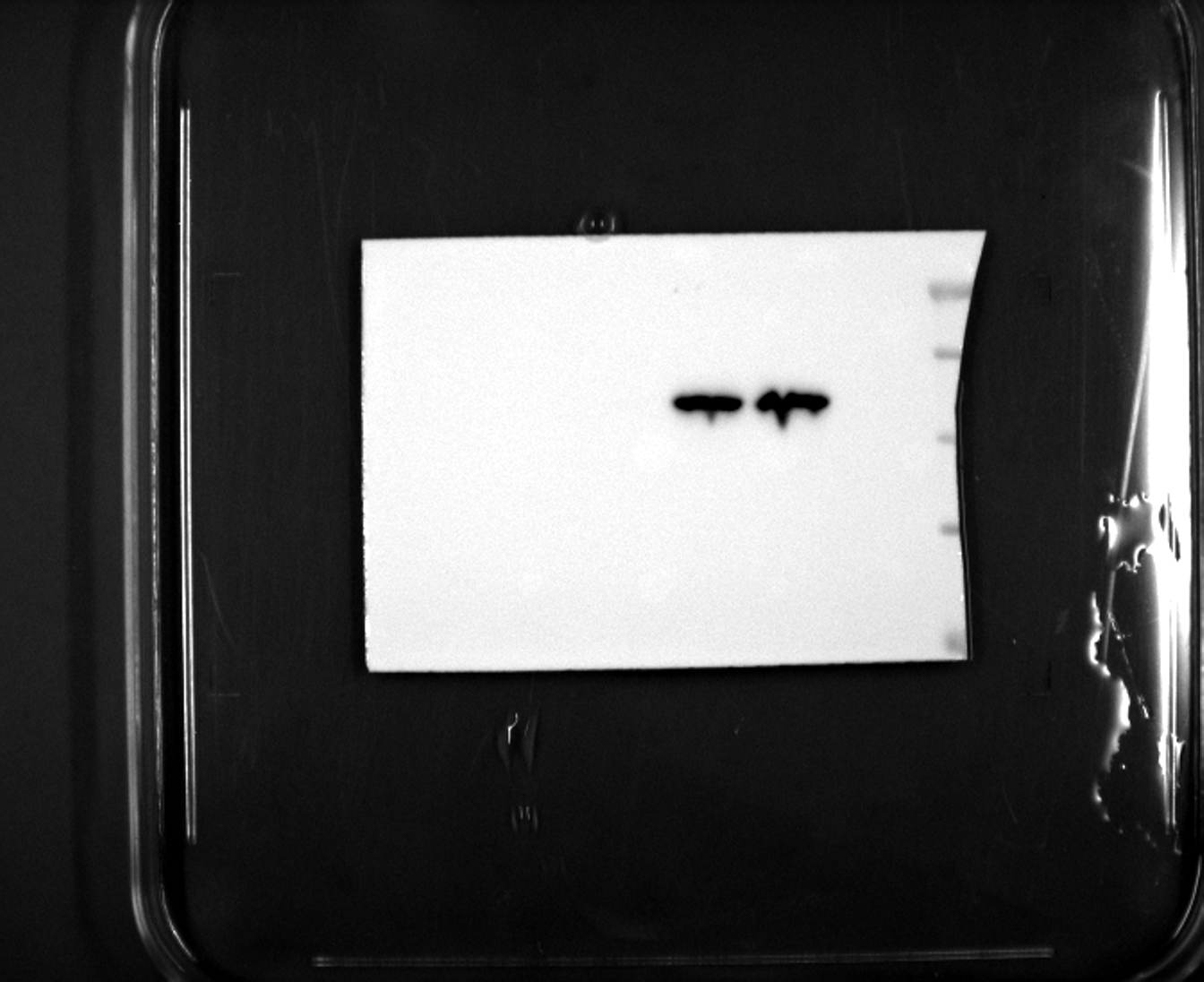

Supplement: Figure 3—figure supplement 1—source data 1. [file elife-97196-fig3-figsupp1-data1.zip › Figure3-S1-SourceData1/Figure3-S1B/Figure 3-figure supplement 1B a- ha sumo cd kd.tif]

Figure 3-figure supplement 1B

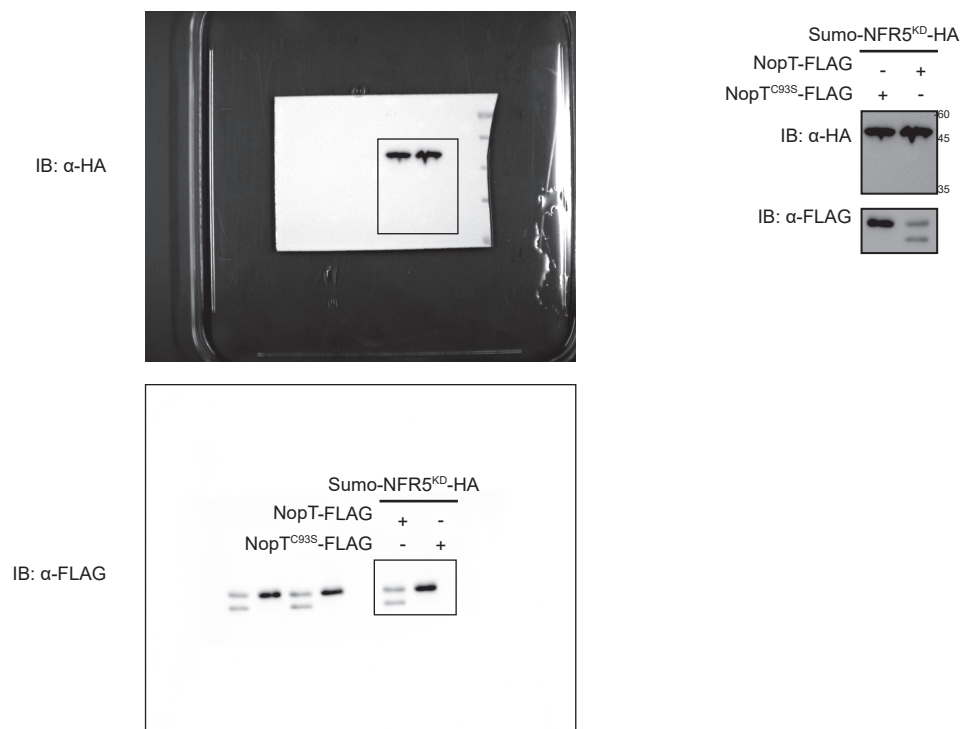

Supplement: Figure 3—figure supplement 1—source data 2. [file elife-97196-fig3-figsupp1-data2.zip › Figure3-S1-SourceData2/Figure3-S1B.pdf]

Figure 3-figure supplement 1A

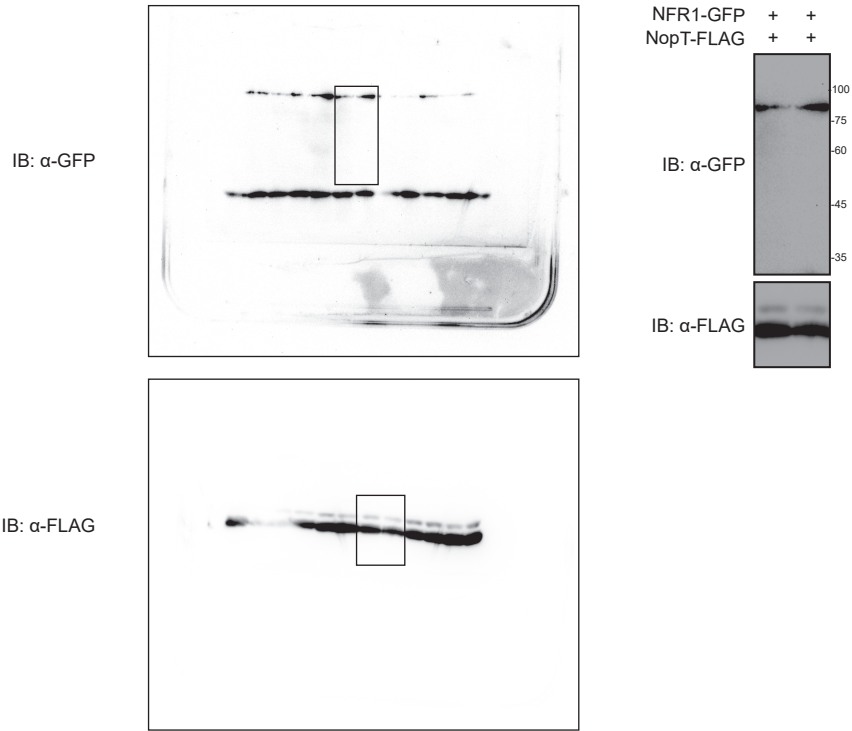

Supplement: Figure 3—figure supplement 1—source data 2. [file elife-97196-fig3-figsupp1-data2.zip › Figure3-S1-SourceData2/Figure3-S1A.pdf]

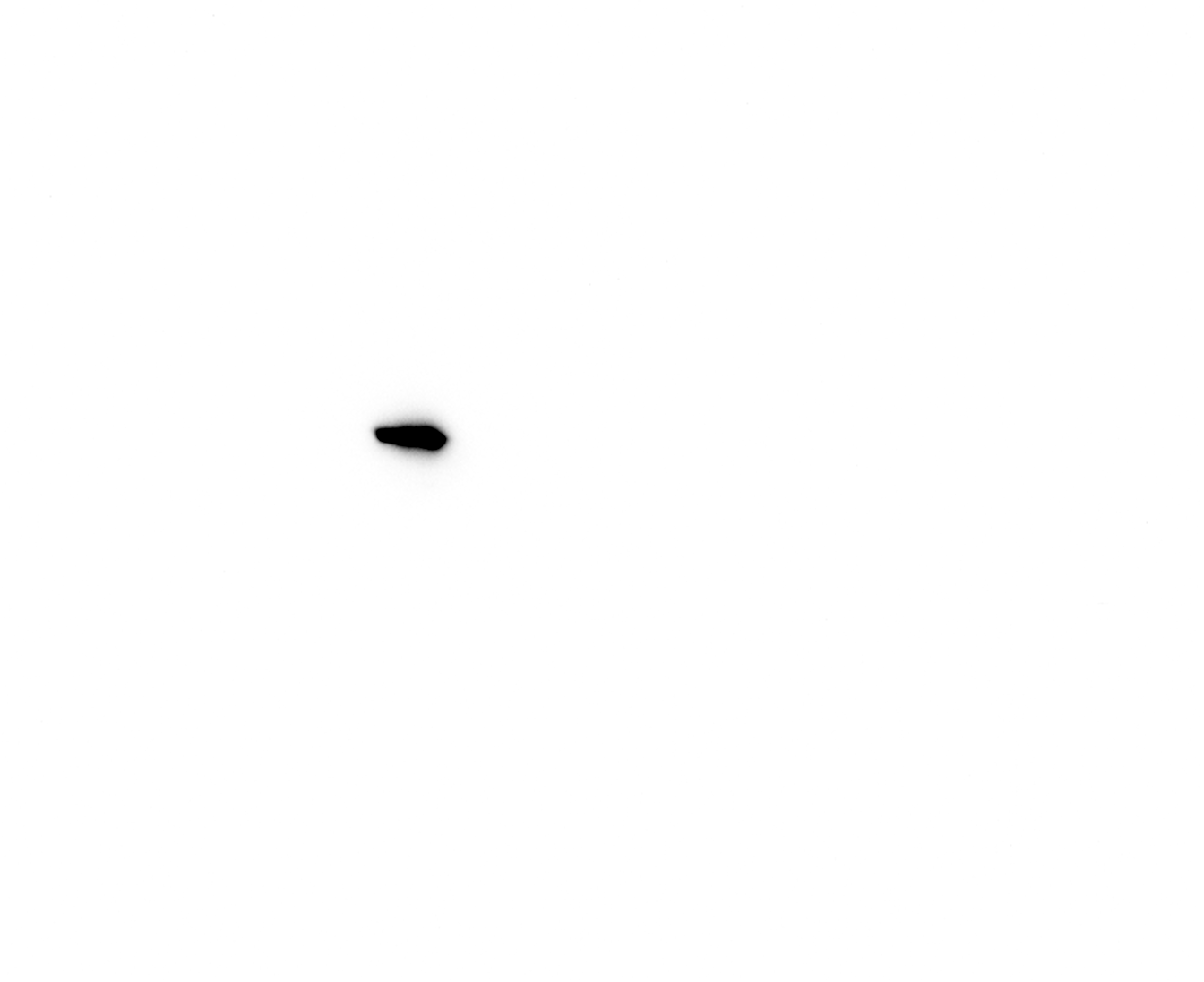

Supplement: Figure 3—figure supplement 2—source data 1. [file elife-97196-fig3-figsupp2-data1.zip › Figure 3-figure supplement 2 IP out a-flag.tif]

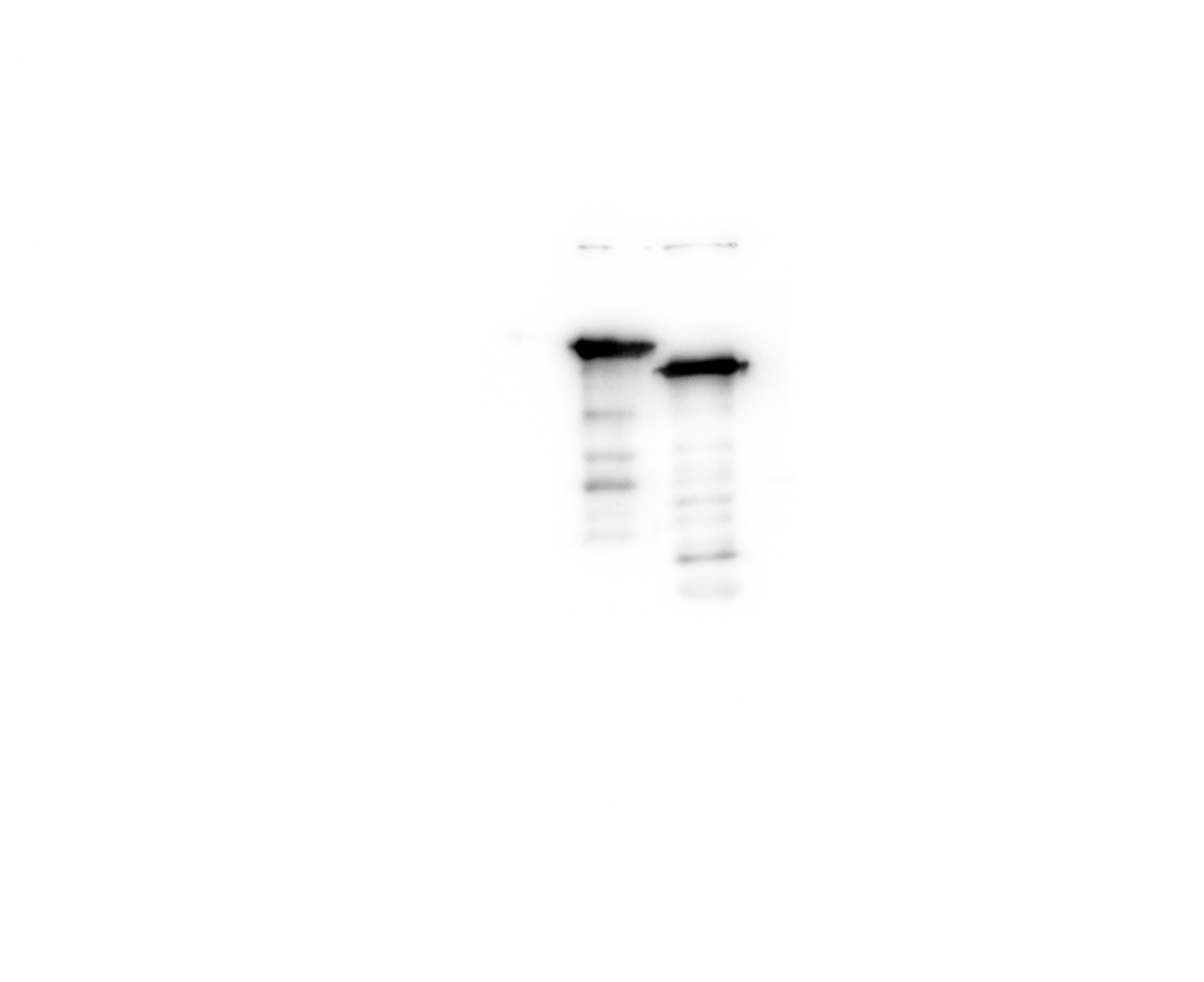

Supplement: Figure 3—figure supplement 2—source data 1. [file elife-97196-fig3-figsupp2-data1.zip › Figure 3-figure supplement 2 ip input a-gfp.tif]

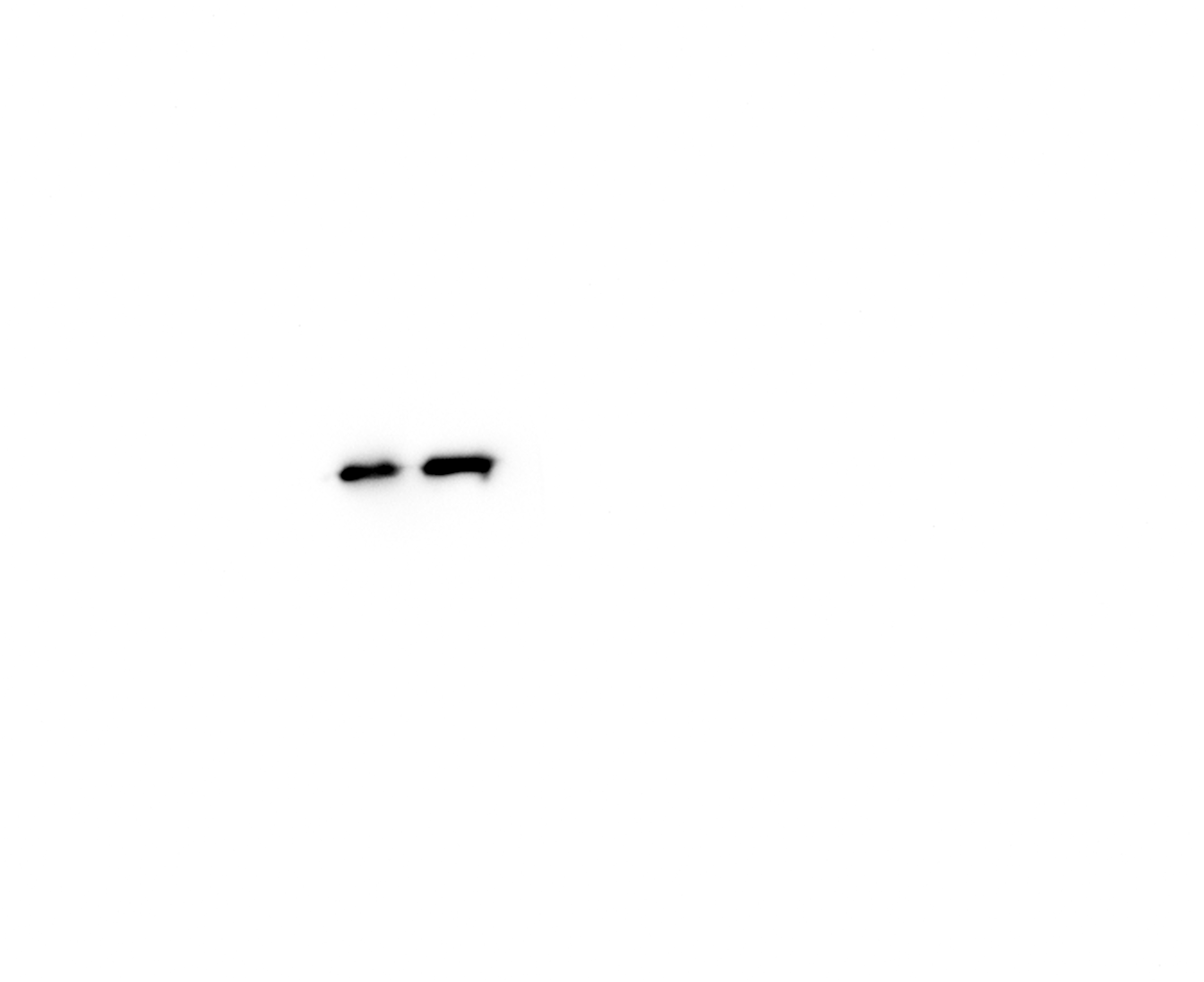

Supplement: Figure 3—figure supplement 2—source data 1. [file elife-97196-fig3-figsupp2-data1.zip › Figure 3-figure supplement 2 ip input a-flag.tif]

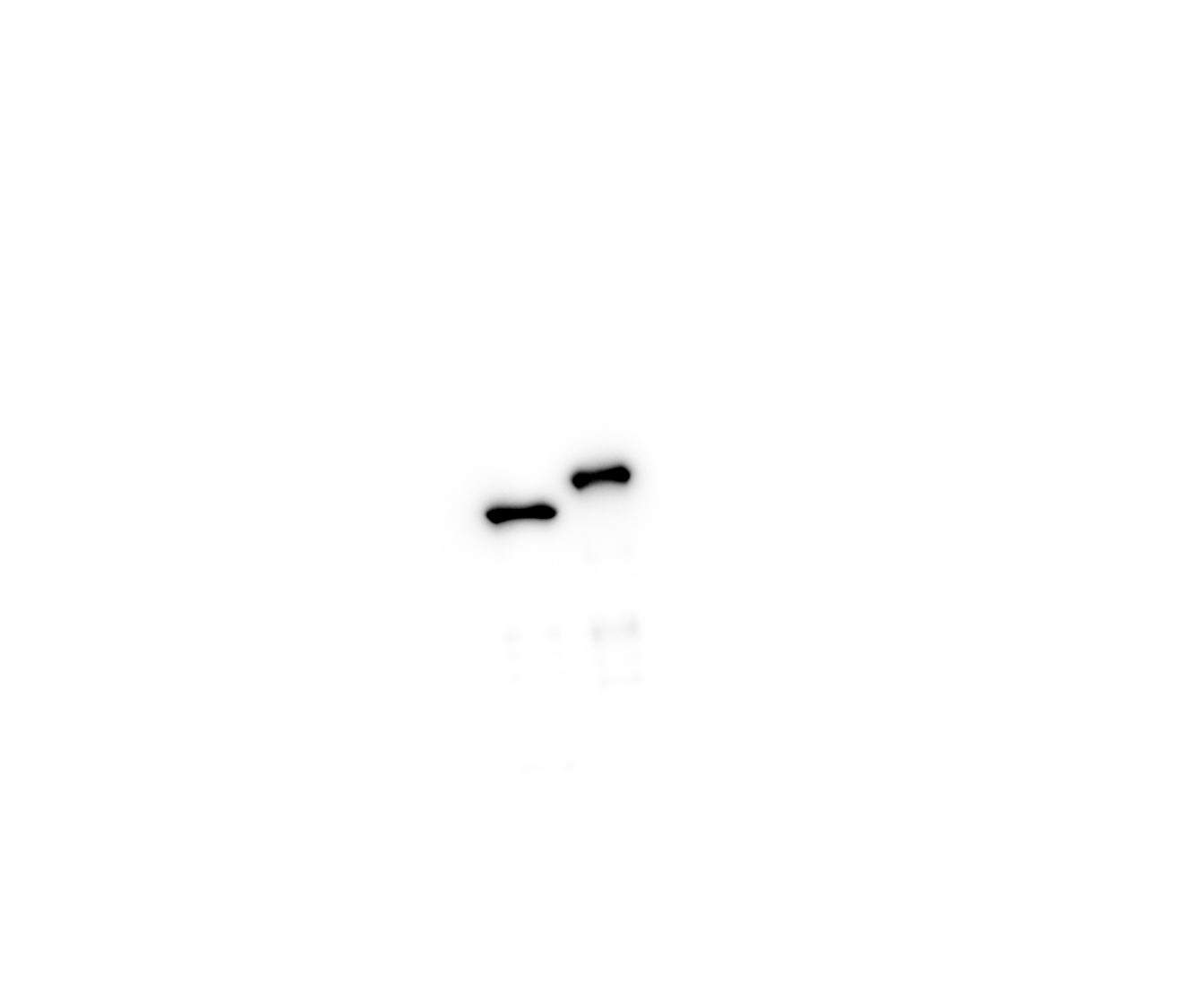

Supplement: Figure 3—figure supplement 2—source data 1. [file elife-97196-fig3-figsupp2-data1.zip › Figure 3-figure supplement 2 ip out a-gfp .tif]

Figure 3-figure supplement 2

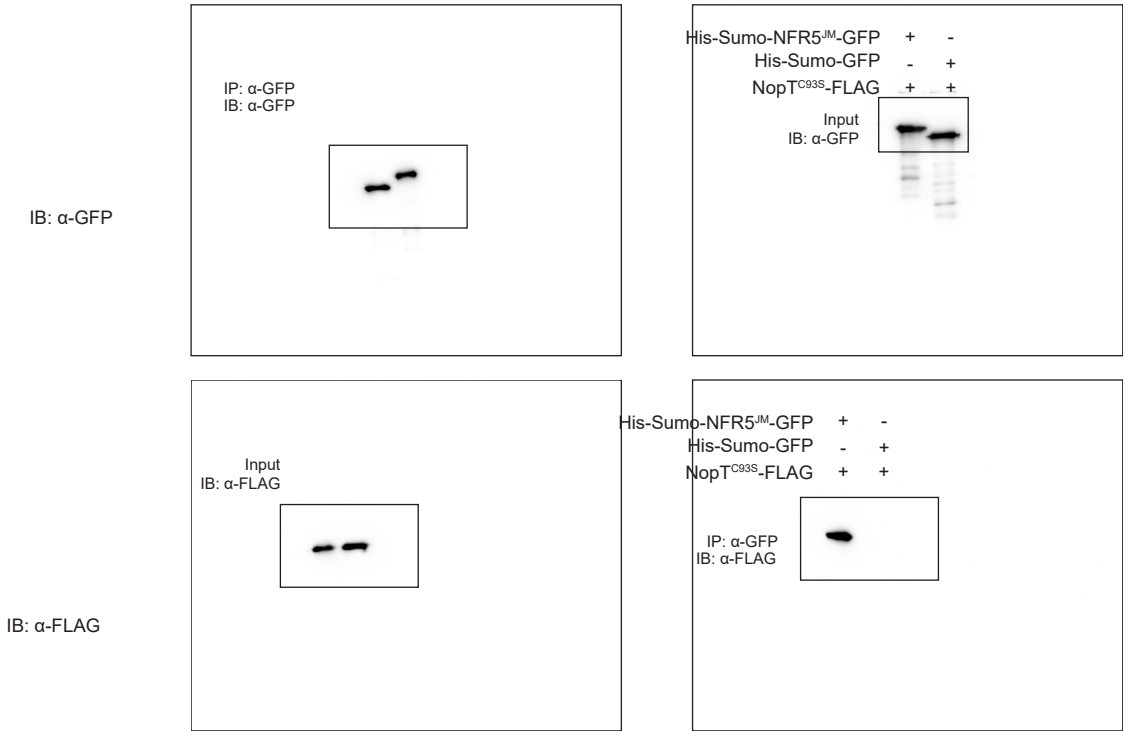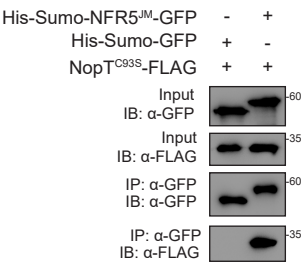

Supplement: Figure 3—figure supplement 2—source data 2. [file elife-97196-fig3-figsupp2-data2.pdf]

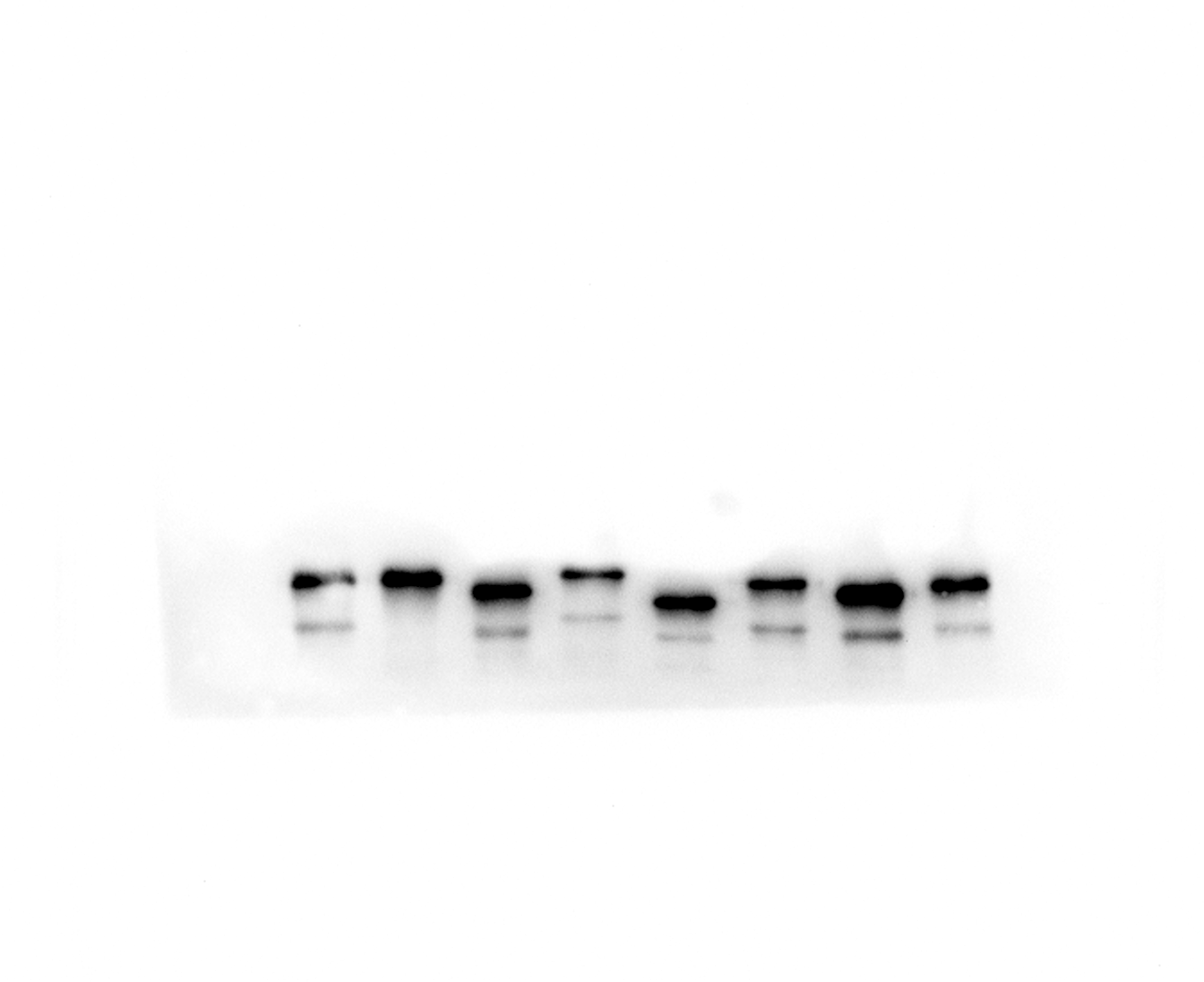

Supplement: Figure 3—figure supplement 4—source data 1. [file elife-97196-fig3-figsupp4-data1.zip › Figure3-S4-SourceData1/Figure3-S4E/Figure 3-figure supplement 4E a-ha .tif]

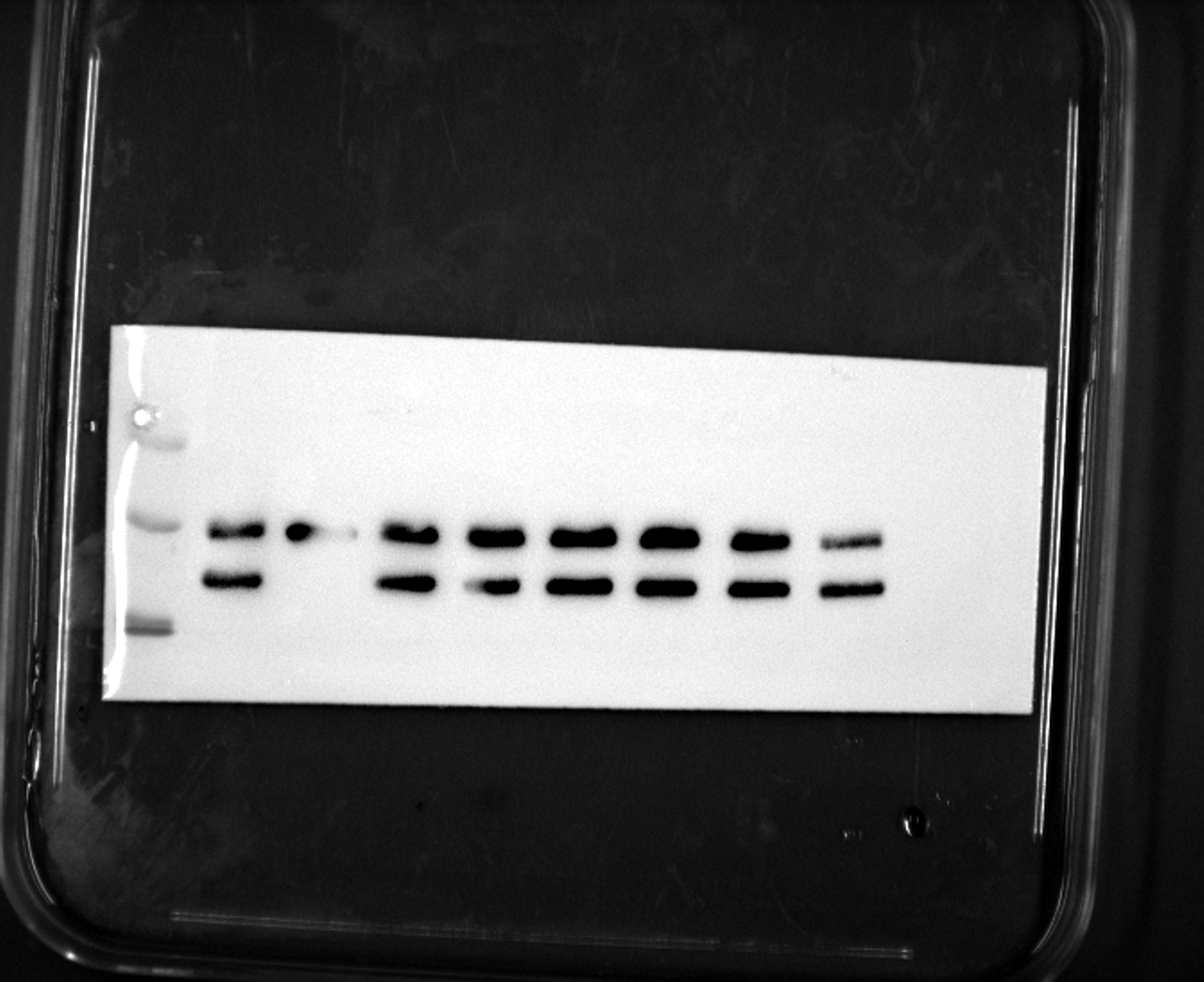

Supplement: Figure 3—figure supplement 4—source data 1. [file elife-97196-fig3-figsupp4-data1.zip › Figure3-S4-SourceData1/Figure3-S4E/Figure 3-figure supplement 4E a- flag .tif]

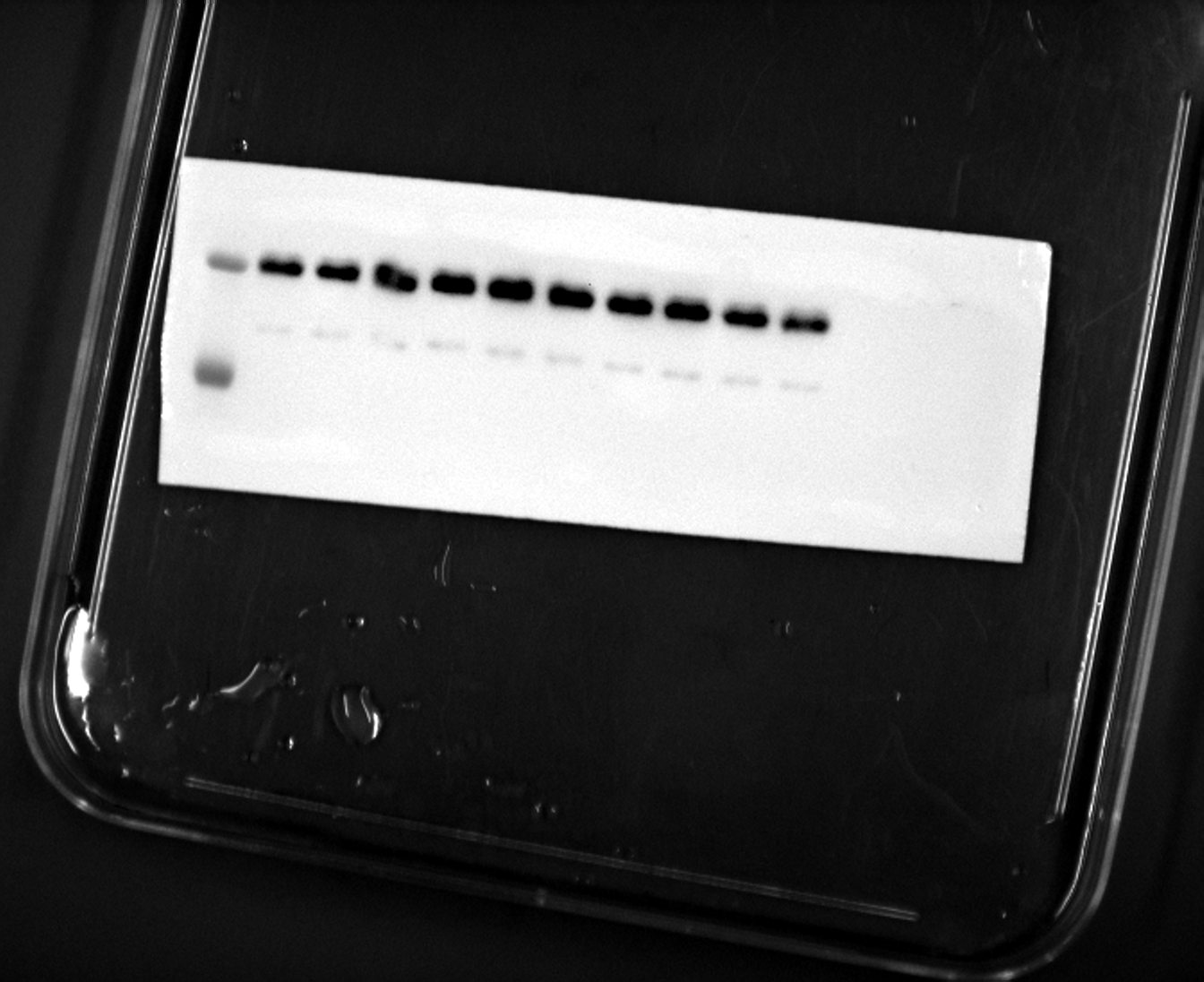

Supplement: Figure 3—figure supplement 4—source data 1. [file elife-97196-fig3-figsupp4-data1.zip › Figure3-S4-SourceData1/Figure3-S4B/Figure 3-figure supplement 4B A-FLAG.tif]

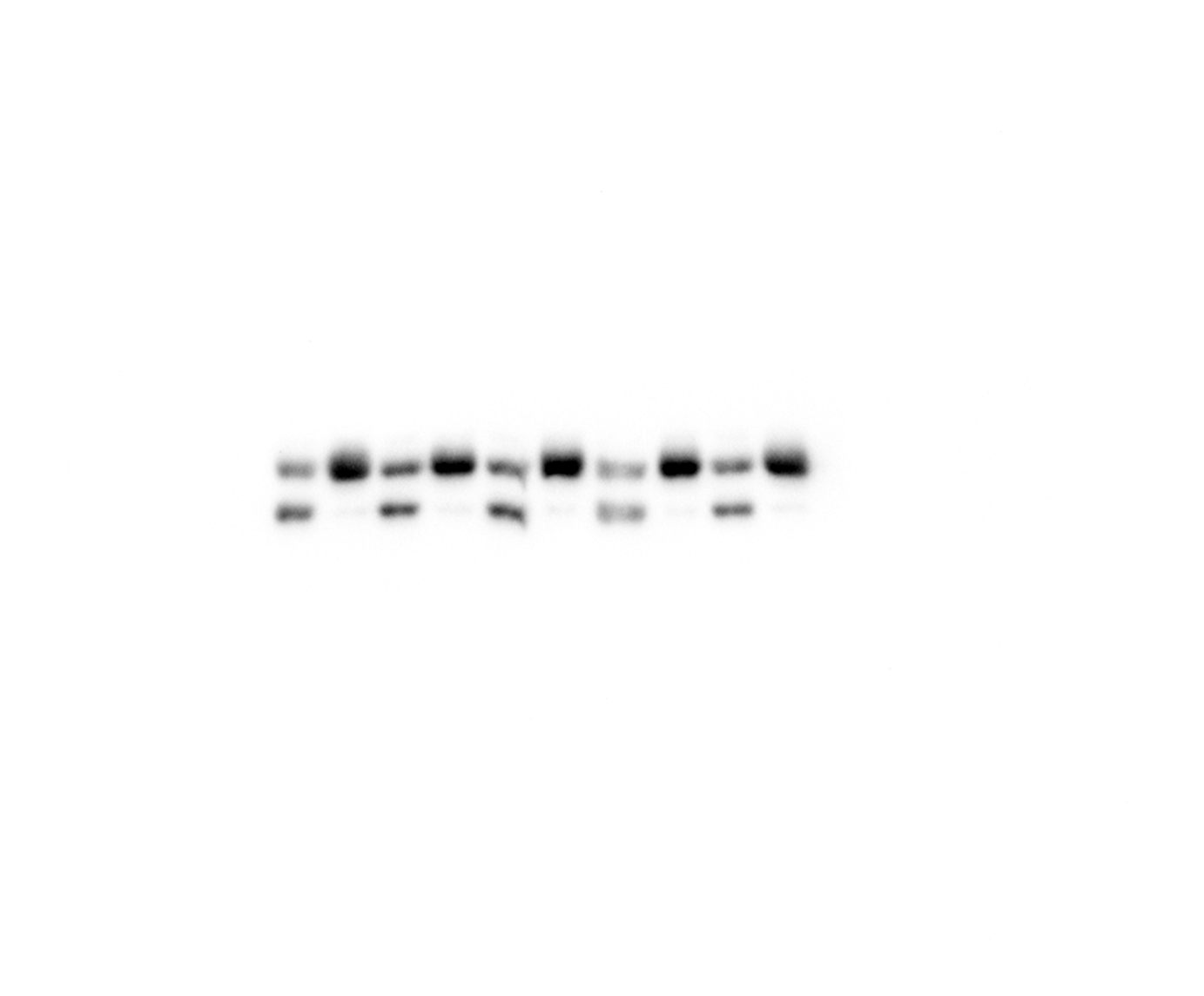

Supplement: Figure 3—figure supplement 4—source data 1. [file elife-97196-fig3-figsupp4-data1.zip › Figure3-S4-SourceData1/Figure3-S4B/Figure 3-figure supplement 4B a-strep.tif]

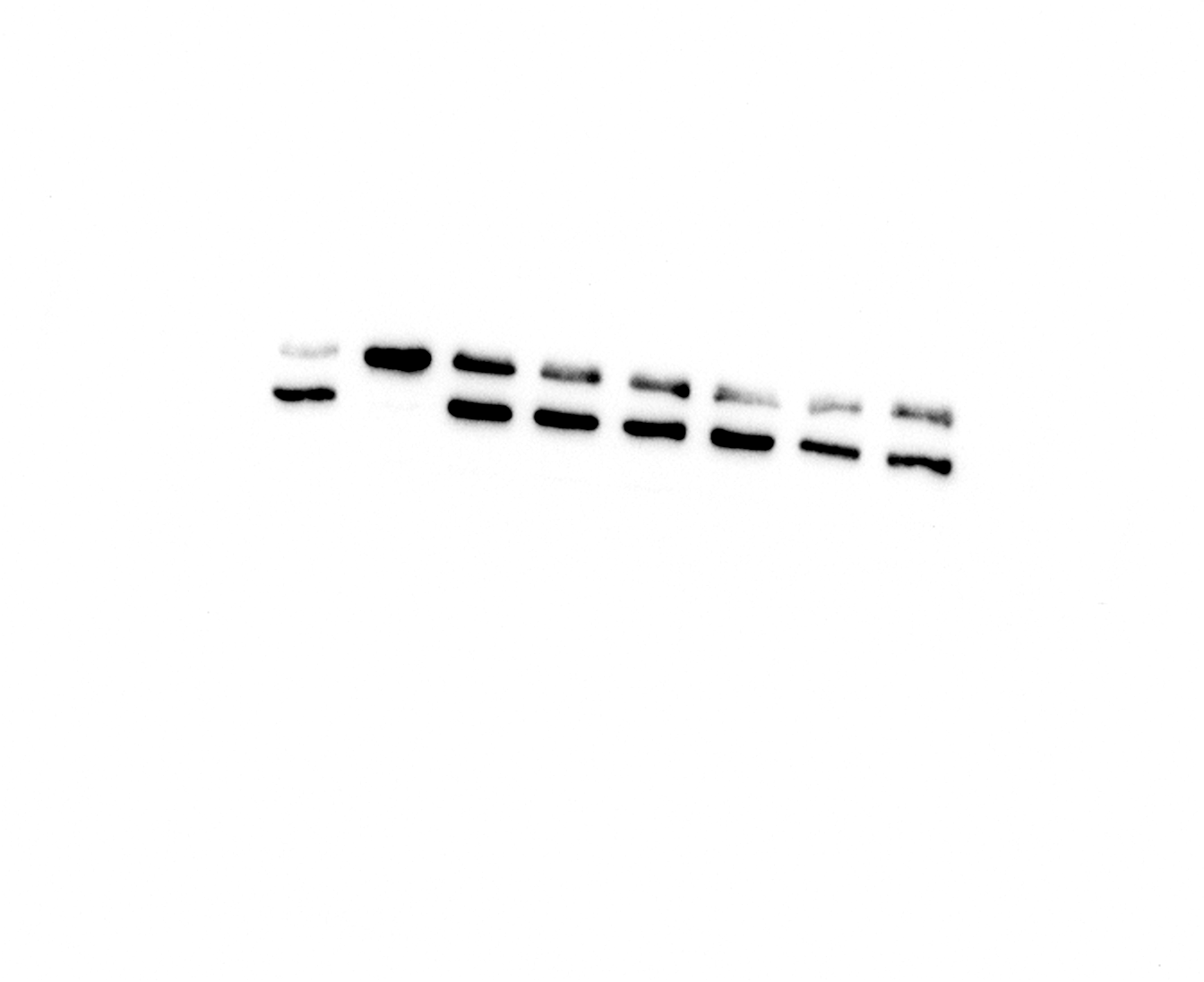

Supplement: Figure 3—figure supplement 4—source data 1. [file elife-97196-fig3-figsupp4-data1.zip › Figure3-S4-SourceData1/Figure3-S4C/Figure 3-figure supplement 4C a- flag.tif]

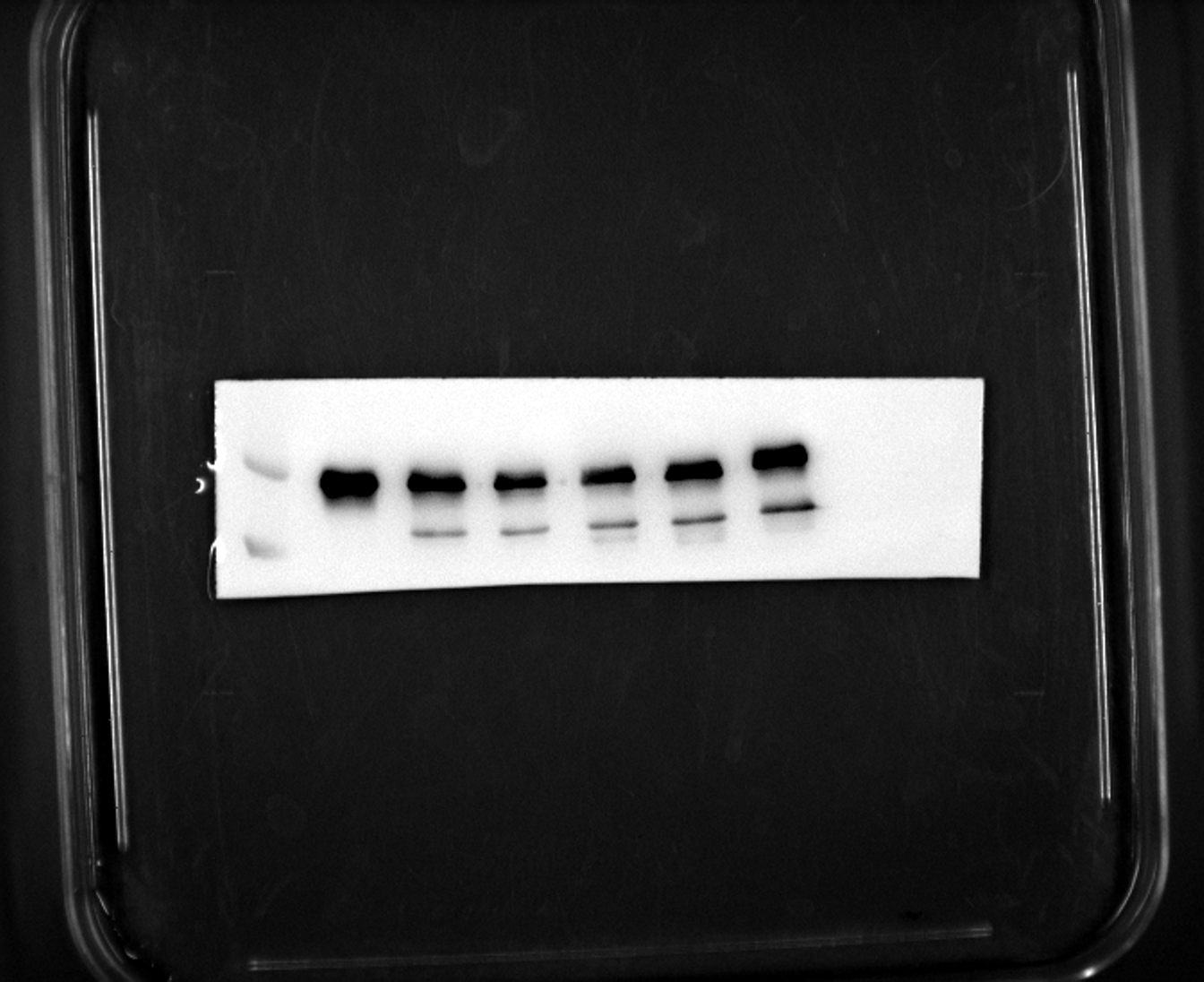

Supplement: Figure 3—figure supplement 4—source data 1. [file elife-97196-fig3-figsupp4-data1.zip › Figure3-S4-SourceData1/Figure3-S4C/Figure 3-figure supplement 4C a-ha.tif]

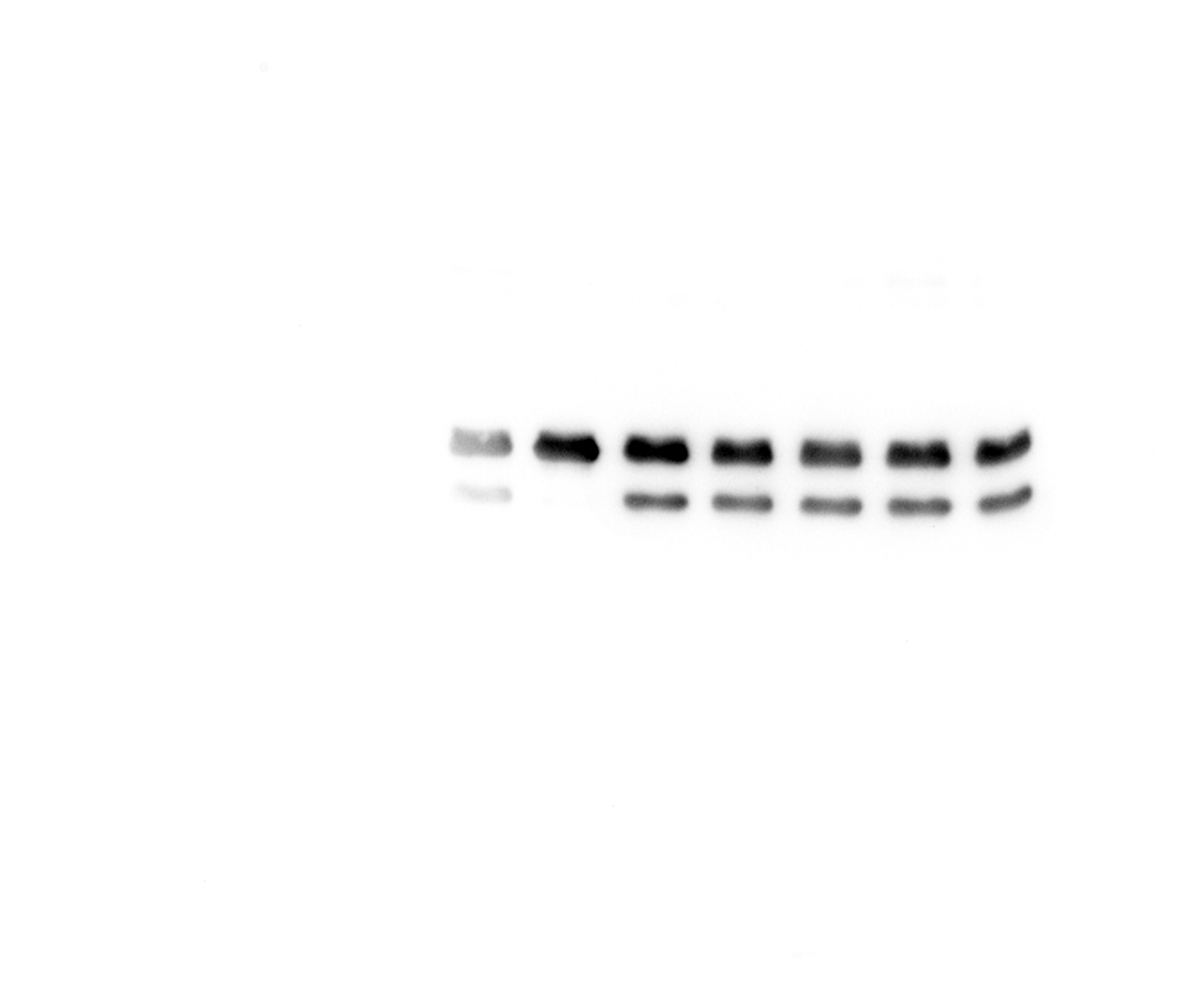

Supplement: Figure 3—figure supplement 4—source data 1. [file elife-97196-fig3-figsupp4-data1.zip › Figure3-S4-SourceData1/Figure3-S4D/Figure 3-figure supplement 4D a-flag .tif]

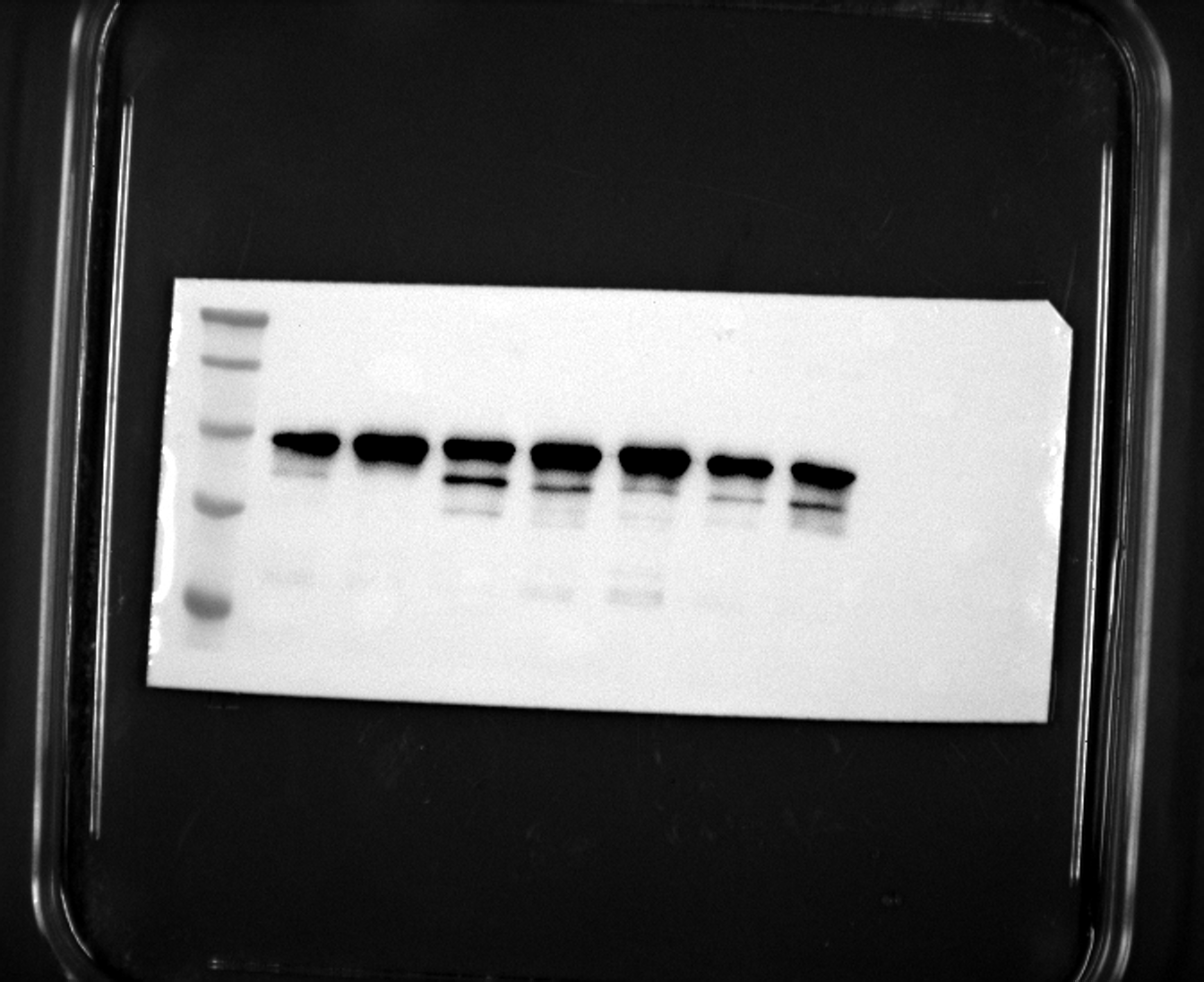

Supplement: Figure 3—figure supplement 4—source data 1. [file elife-97196-fig3-figsupp4-data1.zip › Figure3-S4-SourceData1/Figure3-S4D/Figure 3-figure supplement 4D a-ha .tif]

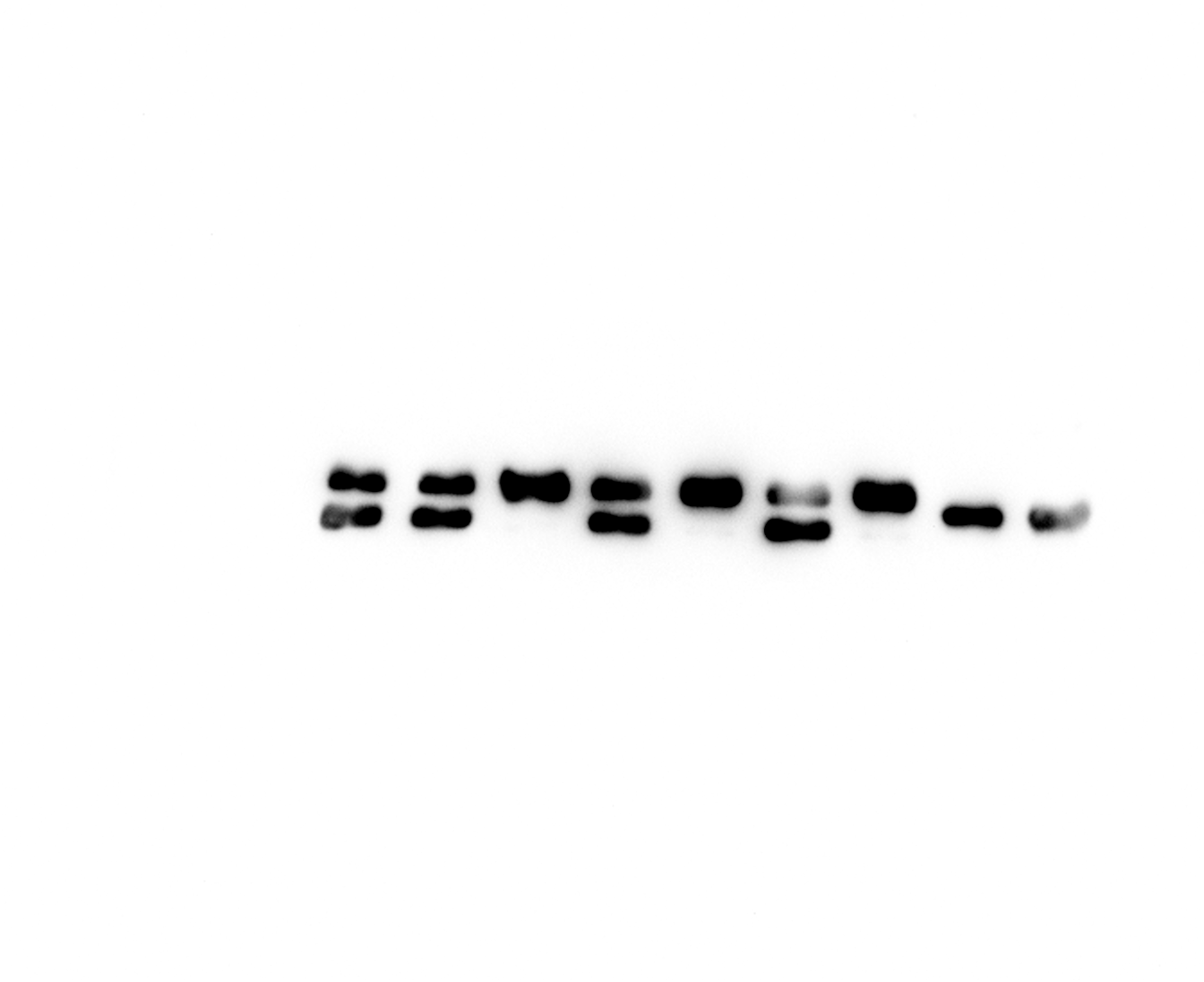

Supplement: Figure 3—figure supplement 4—source data 1. [file elife-97196-fig3-figsupp4-data1.zip › Figure3-S4-SourceData1/Figure3-S4A/Figure 3-figure supplement 4A a-flag.tif]

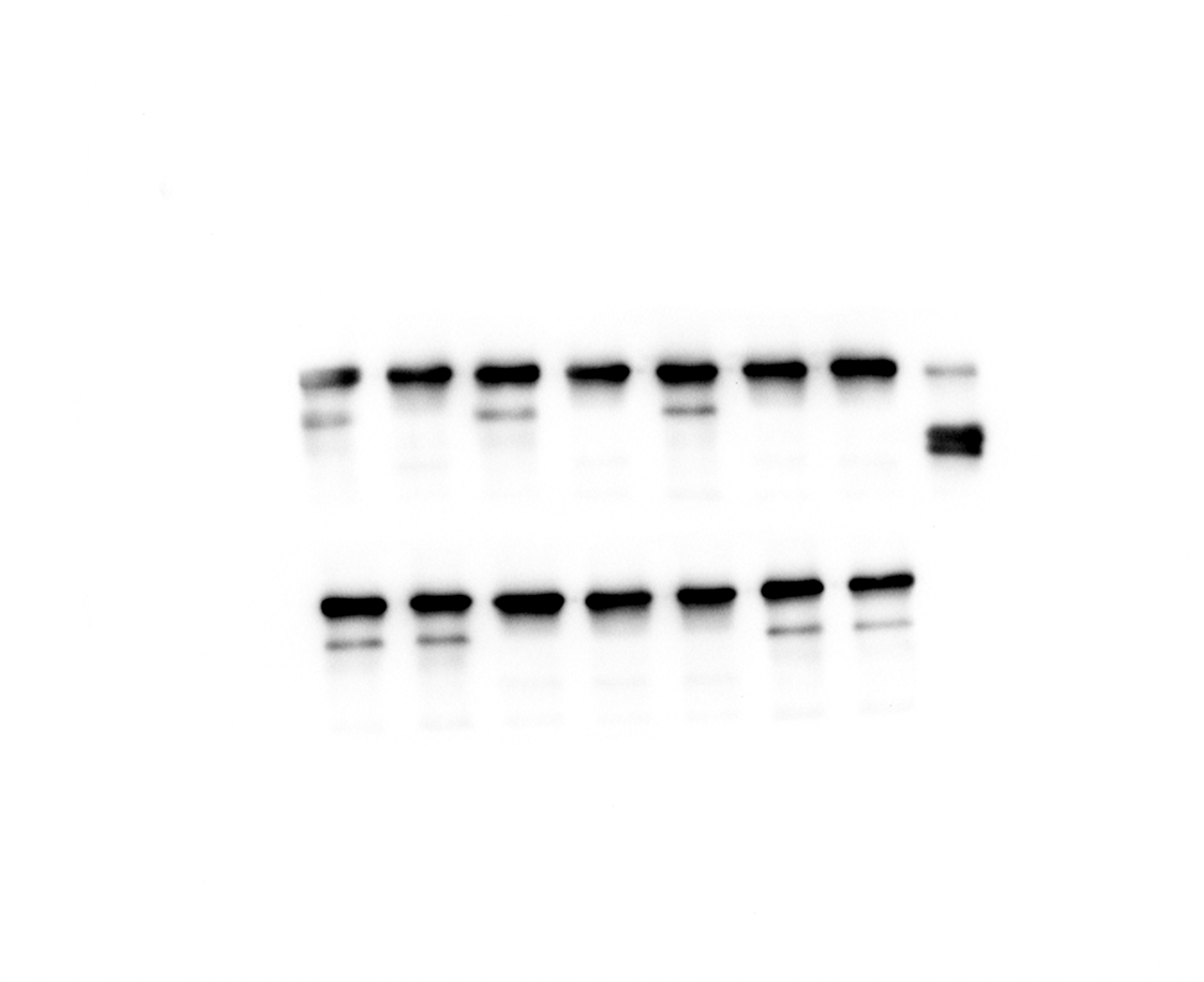

Supplement: Figure 3—figure supplement 4—source data 1. [file elife-97196-fig3-figsupp4-data1.zip › Figure3-S4-SourceData1/Figure3-S4A/Figure 3-figure supplement 4A A-HA.tif]

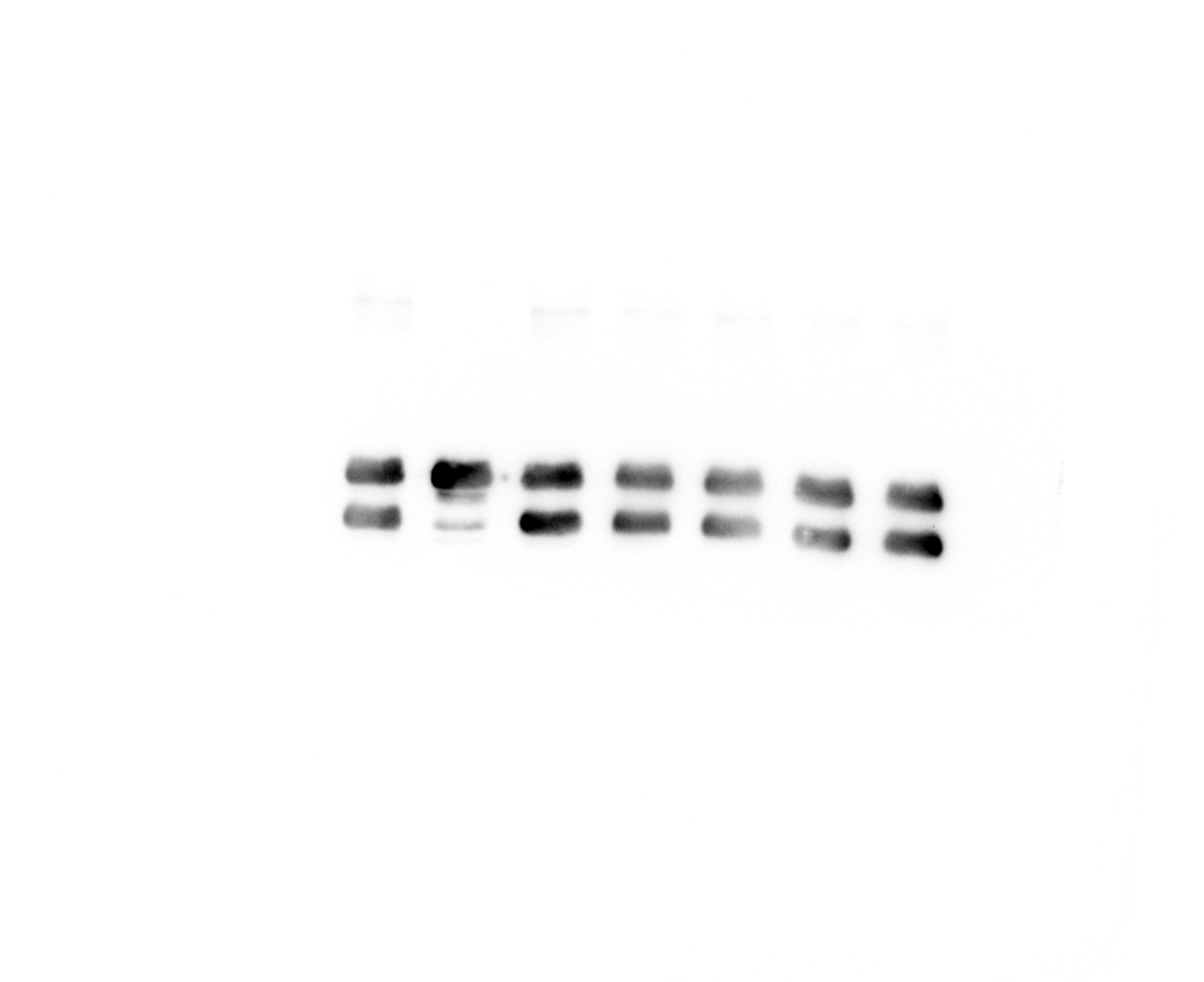

Supplement: Figure 3—figure supplement 4—source data 1. [file elife-97196-fig3-figsupp4-data1.zip › Figure3-S4-SourceData1/Figure3-S4F/Figure 3-figure supplement 4F a-flag.tif]

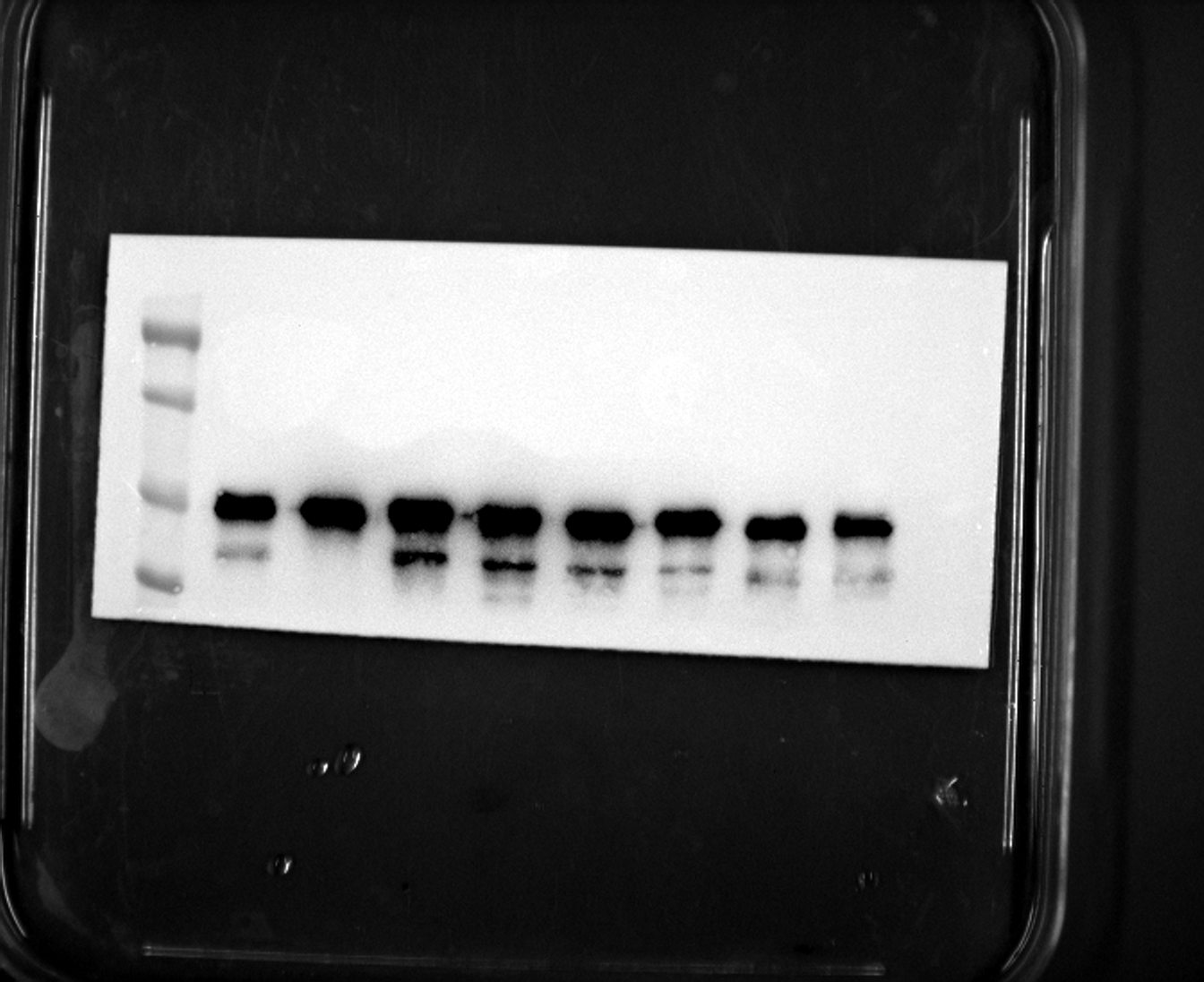

Supplement: Figure 3—figure supplement 4—source data 1. [file elife-97196-fig3-figsupp4-data1.zip › Figure3-S4-SourceData1/Figure3-S4F/Figure 3-figure supplement 4F a-ha .tif]

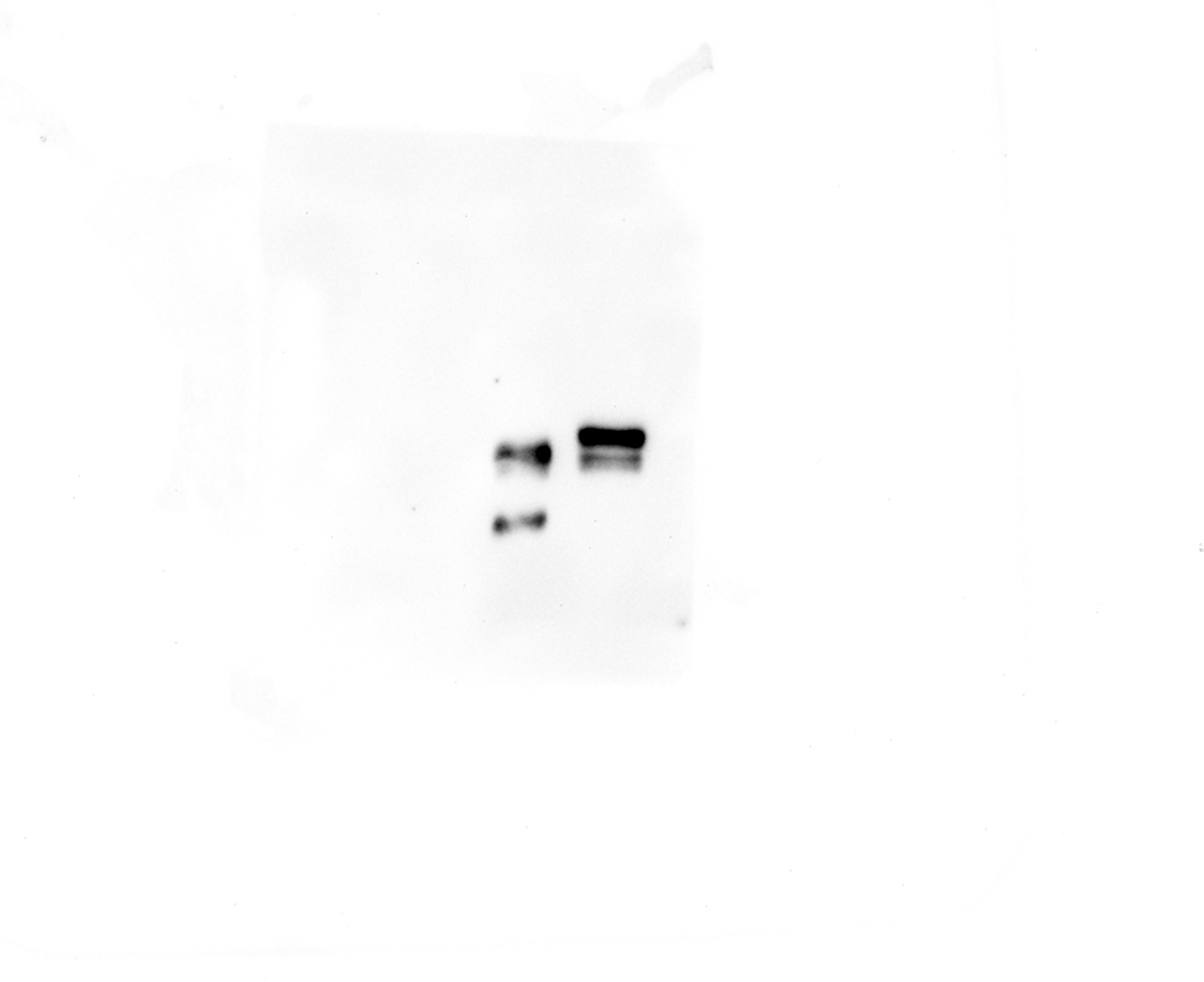

Supplement: Figure 3—figure supplement 4—source data 1. [file elife-97196-fig3-figsupp4-data1.zip › Figure3-S4-SourceData1/Figure3-S4G/Figure 3-figure supplement 4G a-flag268-277de.tif]

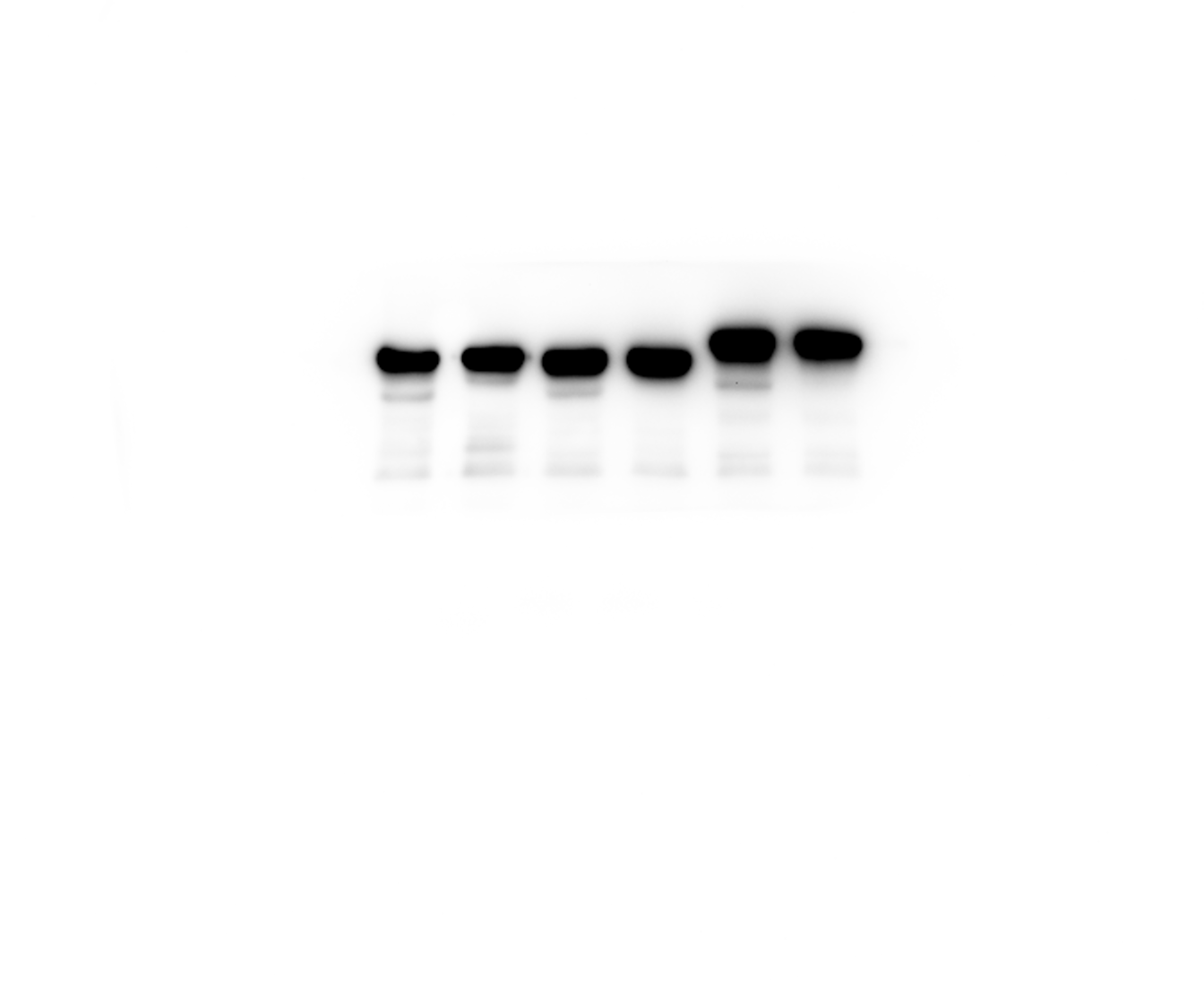

Supplement: Figure 3—figure supplement 4—source data 1. [file elife-97196-fig3-figsupp4-data1.zip › Figure3-S4-SourceData1/Figure3-S4G/Figure 3-figure supplement 4G a-HA 308-337de.tif]

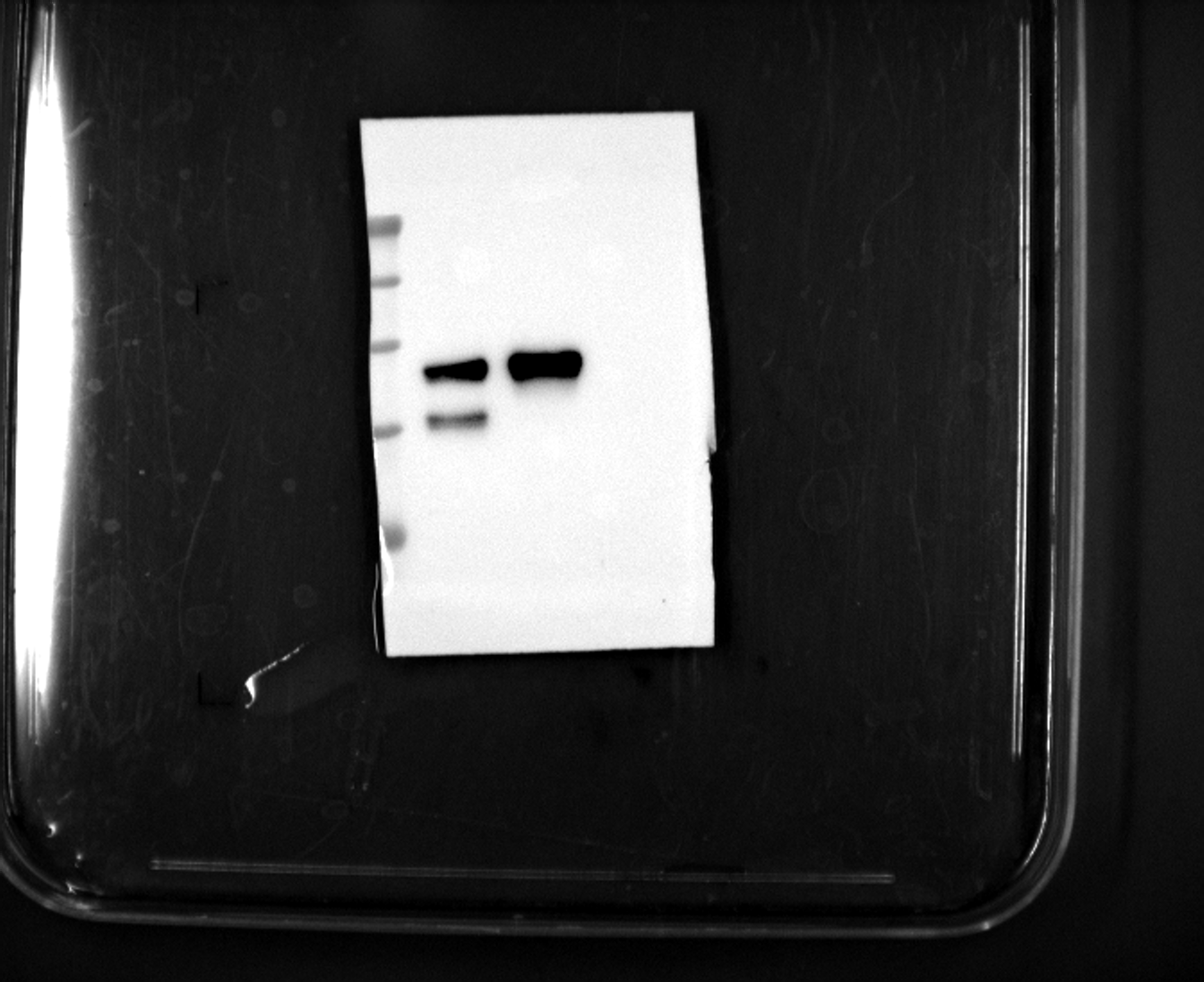

Supplement: Figure 3—figure supplement 4—source data 1. [file elife-97196-fig3-figsupp4-data1.zip › Figure3-S4-SourceData1/Figure3-S4G/Figure 3-figure supplement 4G a-ha 268-277de.tif]

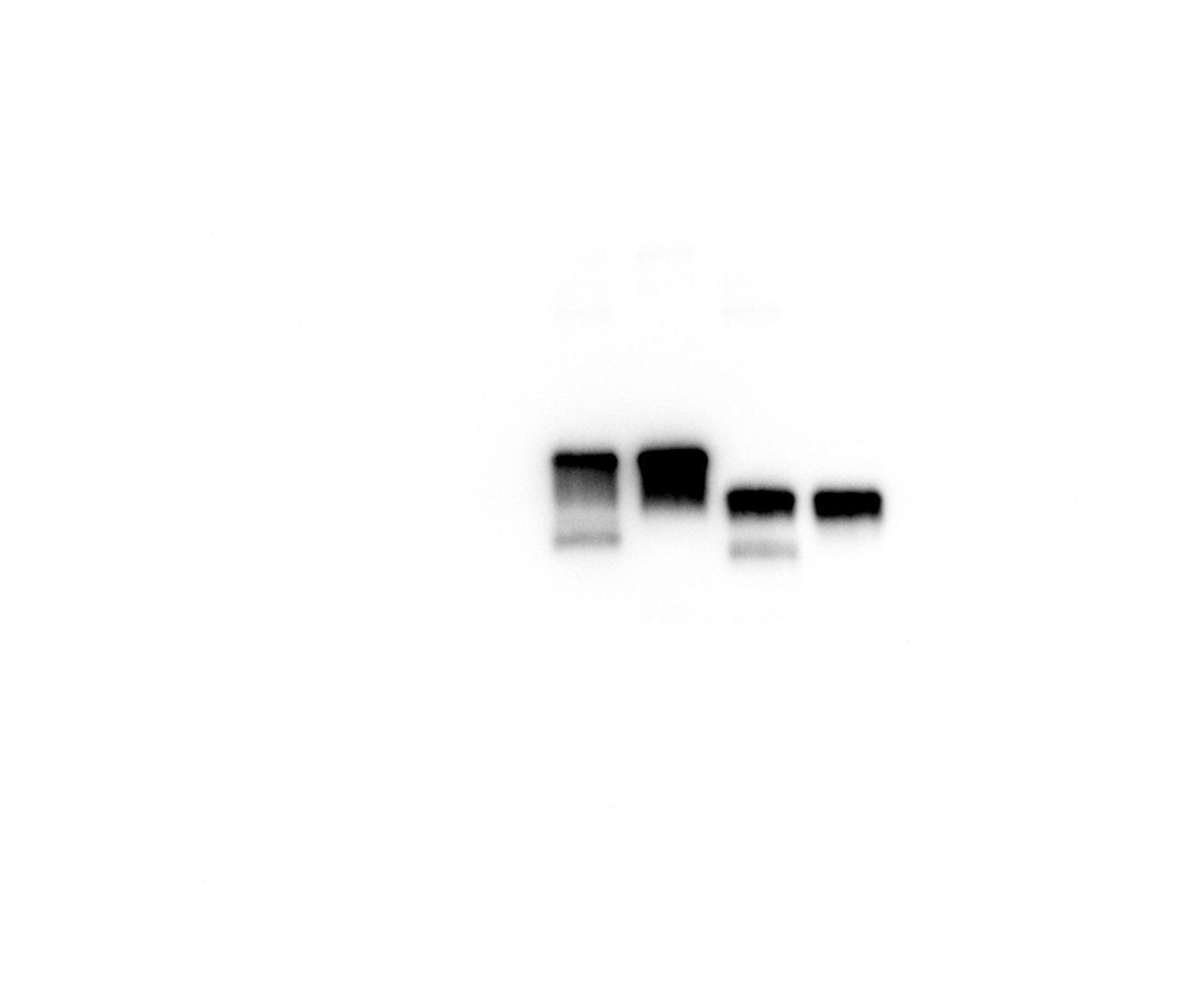

Supplement: Figure 3—figure supplement 4—source data 1. [file elife-97196-fig3-figsupp4-data1.zip › Figure3-S4-SourceData1/Figure3-S4G/Figure 3-figure supplement 4G a-ha 288-307 de.tif]

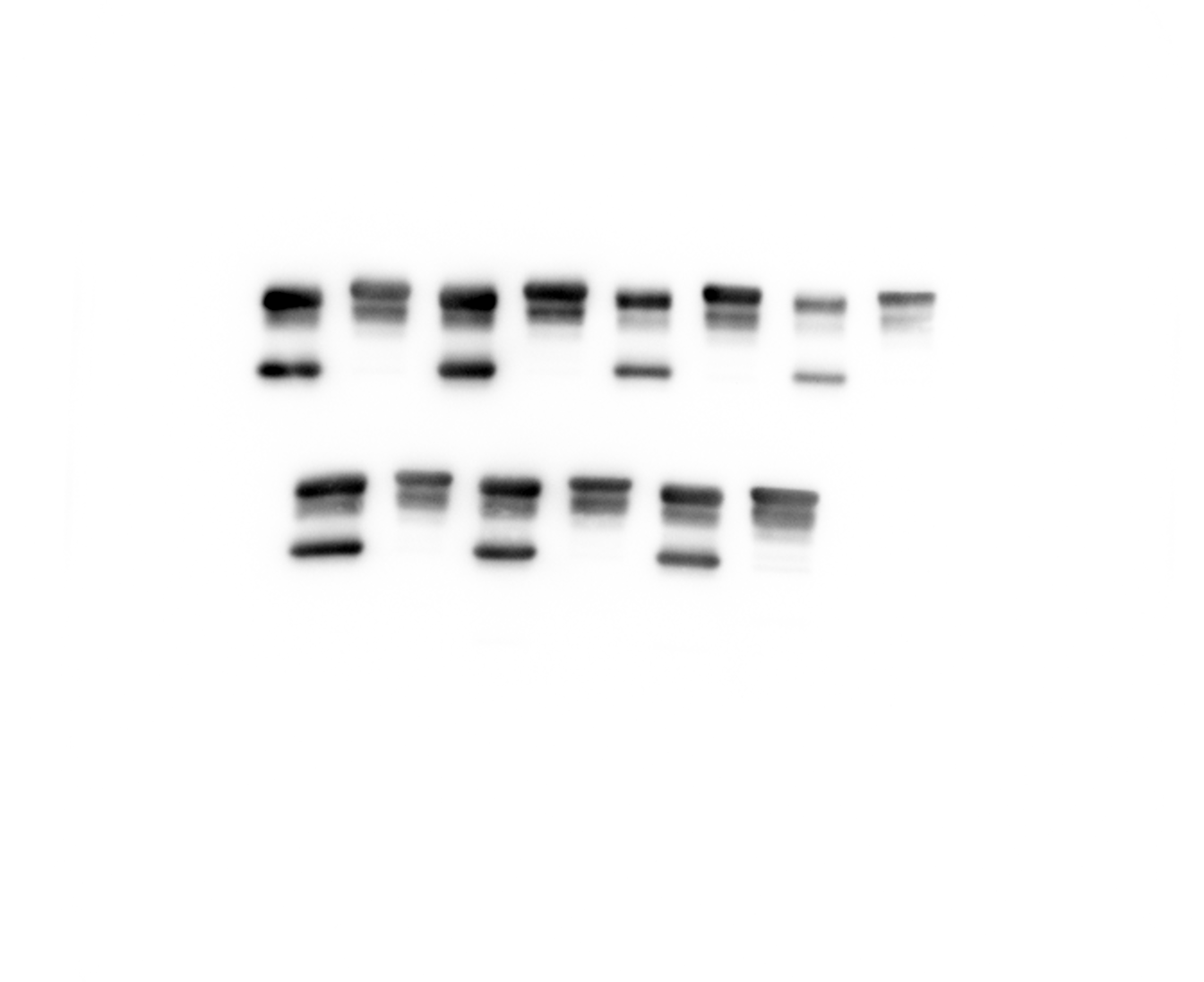

Supplement: Figure 3—figure supplement 4—source data 1. [file elife-97196-fig3-figsupp4-data1.zip › Figure3-S4-SourceData1/Figure3-S4G/Figure 3-figure supplement 4G a-flag 288-307 308-337de .tif]

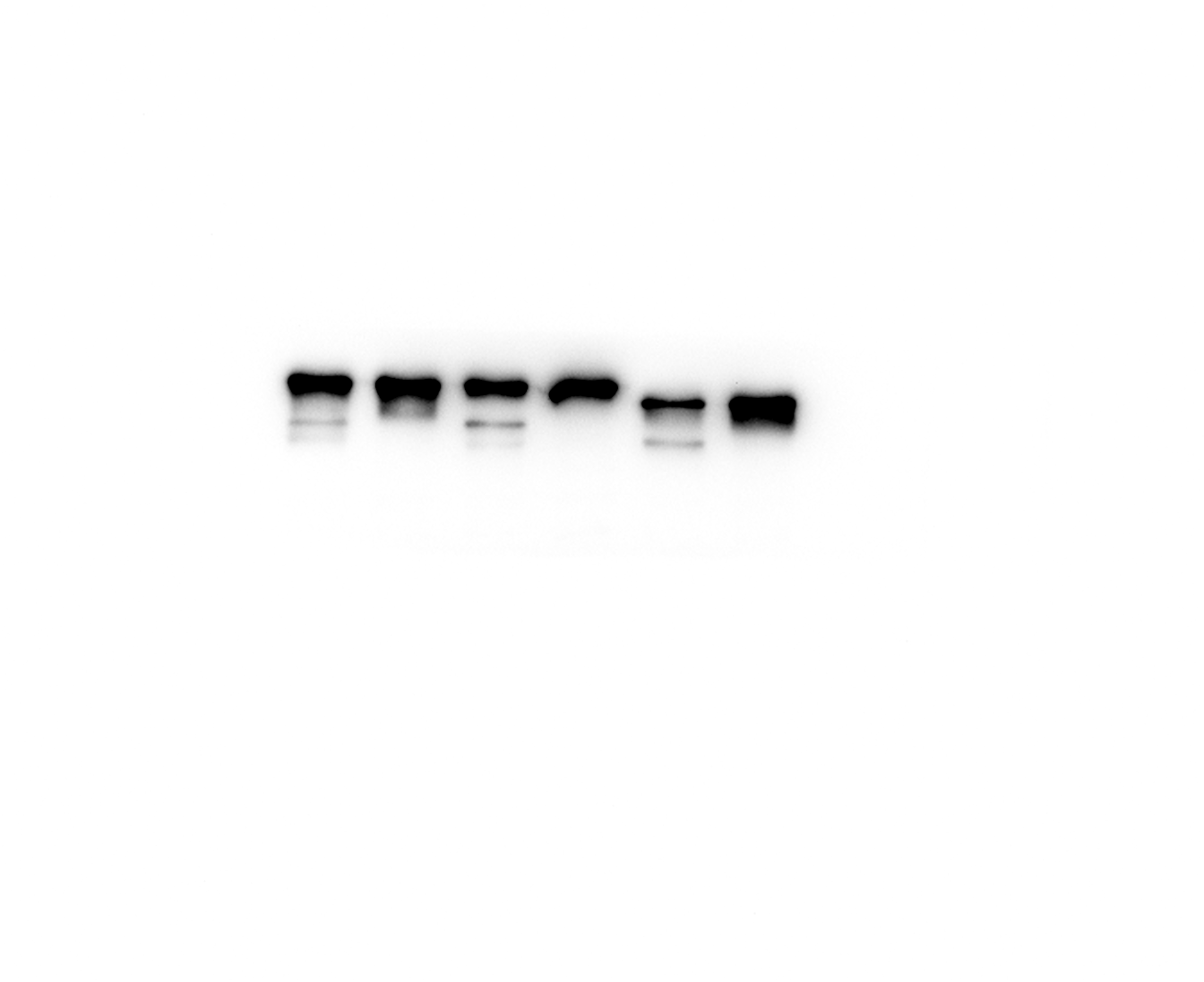

Supplement: Figure 3—figure supplement 4—source data 1. [file elife-97196-fig3-figsupp4-data1.zip › Figure3-S4-SourceData1/Figure3-S4G/Figure 3-figure supplement 4G A-HA 278-288de.tif]

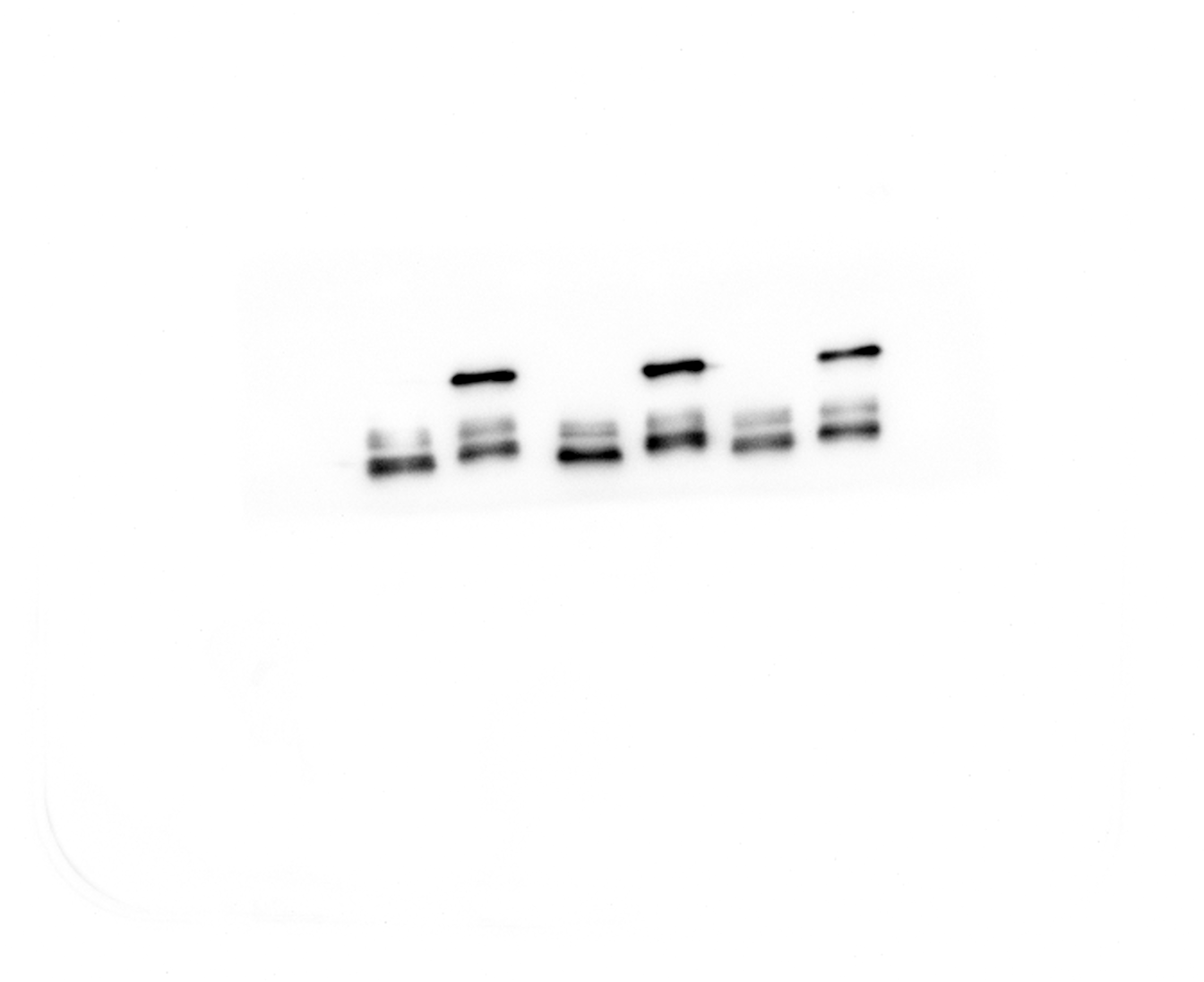

Supplement: Figure 3—figure supplement 4—source data 1. [file elife-97196-fig3-figsupp4-data1.zip › Figure3-S4-SourceData1/Figure3-S4G/Figure 3-figure supplement 4G A-FLAG 278-288de.tif]

Figure 3-figure supplement 4D

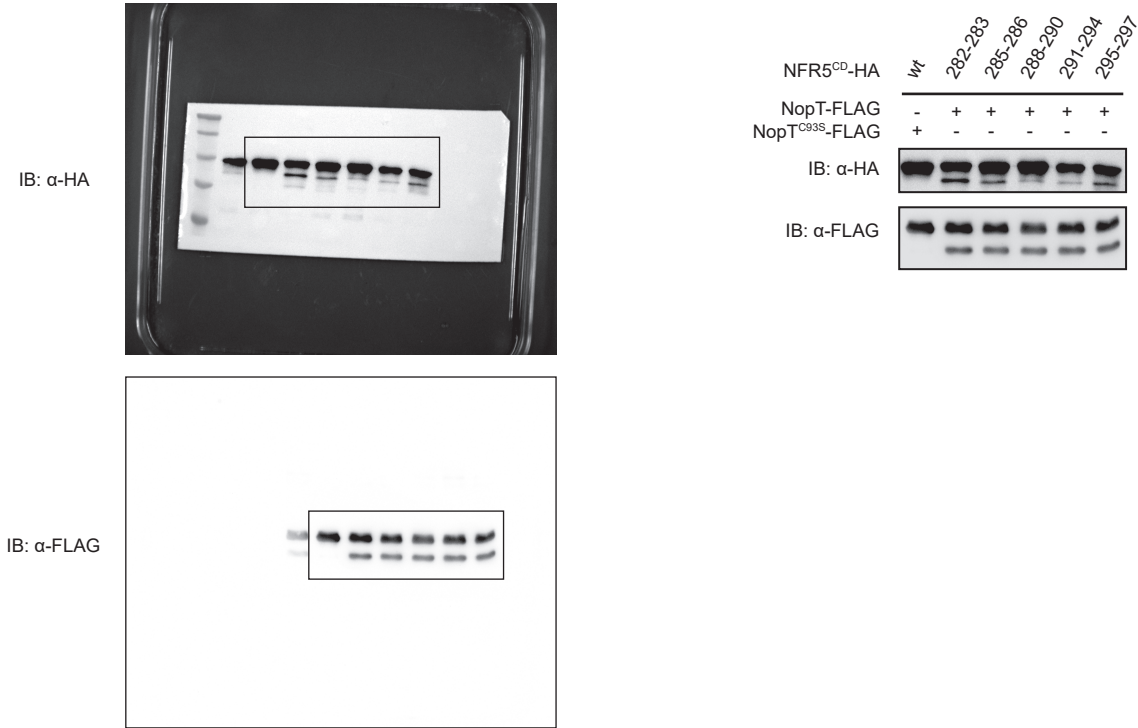

Supplement: Figure 3—figure supplement 4—source data 2. [file elife-97196-fig3-figsupp4-data2.zip › Figure3-S4-SourceData2/Figure3-S4D.pdf]

Figure 3-figure supplement 4E

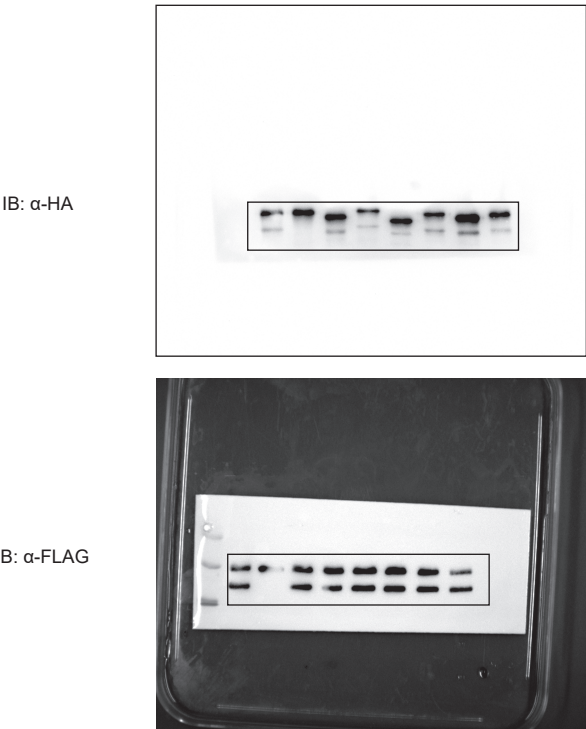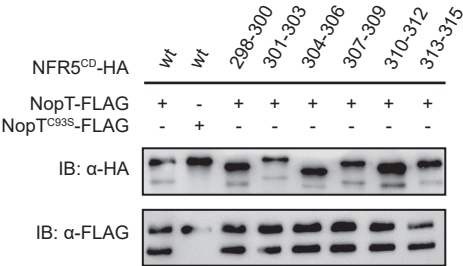

Supplement: Figure 3—figure supplement 4—source data 2. [file elife-97196-fig3-figsupp4-data2.zip › Figure3-S4-SourceData2/Figure3-S4E.pdf]

Figure 3-figure supplement 4G

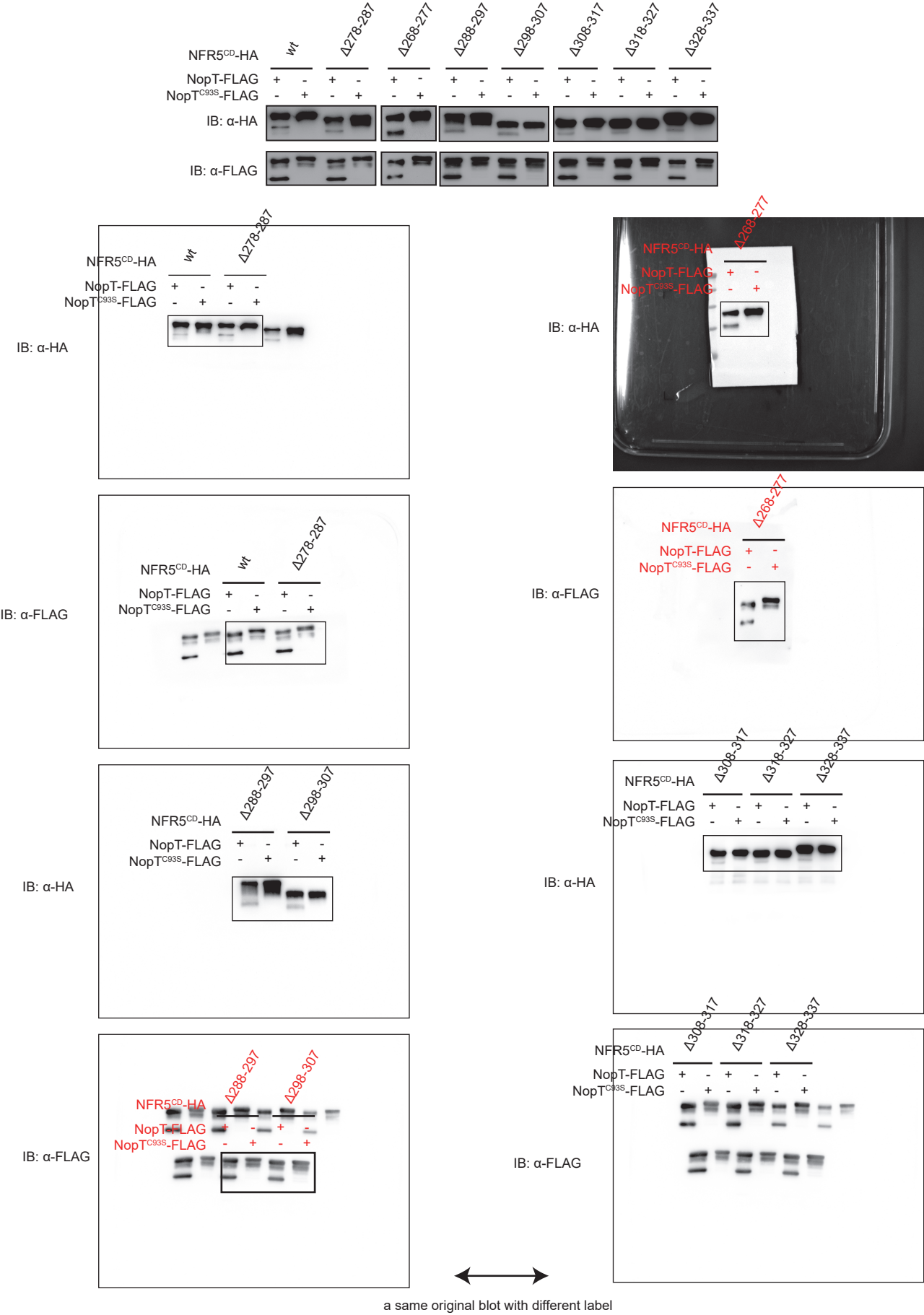

Supplement: Figure 3—figure supplement 4—source data 2. [file elife-97196-fig3-figsupp4-data2.zip › Figure3-S4-SourceData2/Figure3-S4G.pdf]

Figure 3-figure supplement 4F

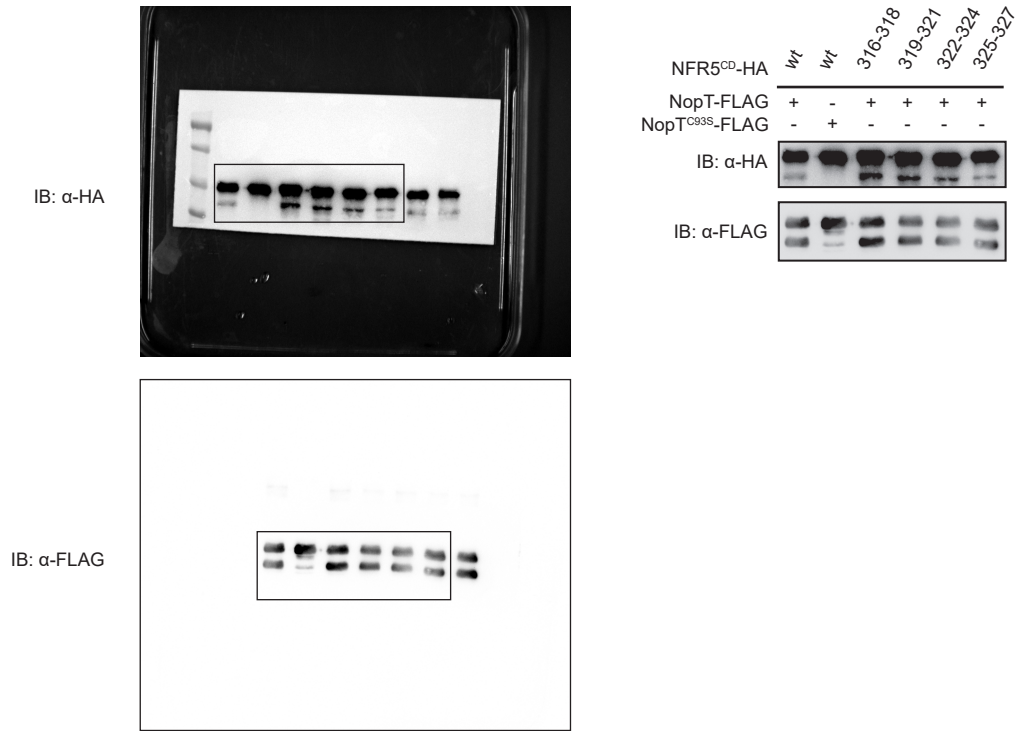

Supplement: Figure 3—figure supplement 4—source data 2. [file elife-97196-fig3-figsupp4-data2.zip › Figure3-S4-SourceData2/Figure3-S4F.pdf]

Figure 3-figure supplement 4B

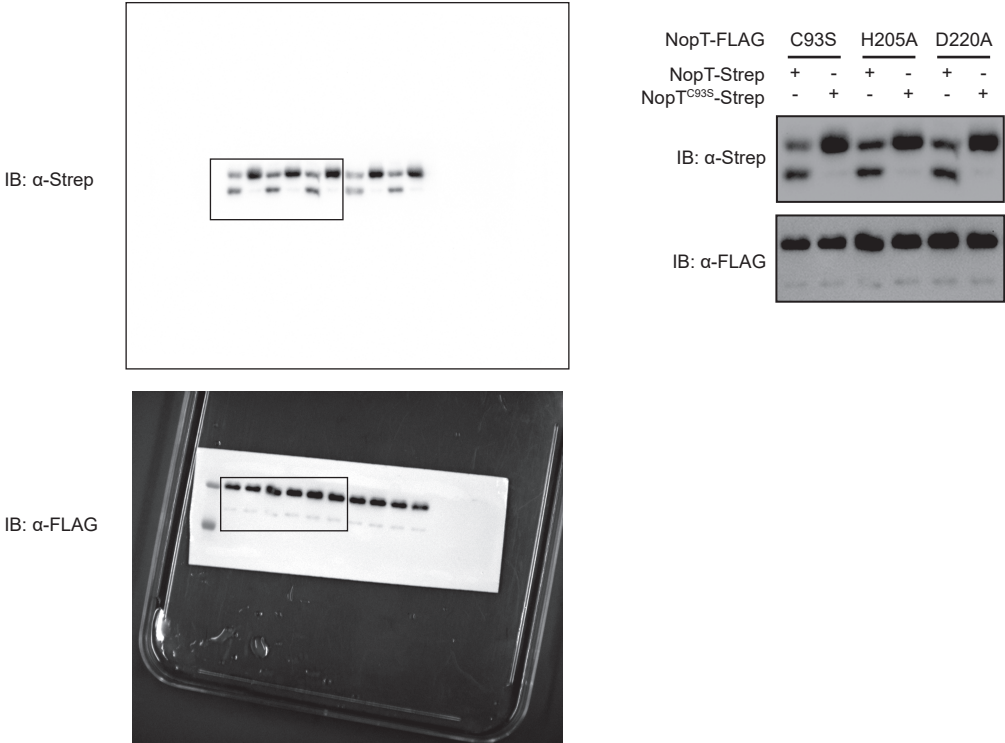

Supplement: Figure 3—figure supplement 4—source data 2. [file elife-97196-fig3-figsupp4-data2.zip › Figure3-S4-SourceData2/Figure3-S4B.pdf]

Figure 3-figure supplement 4C

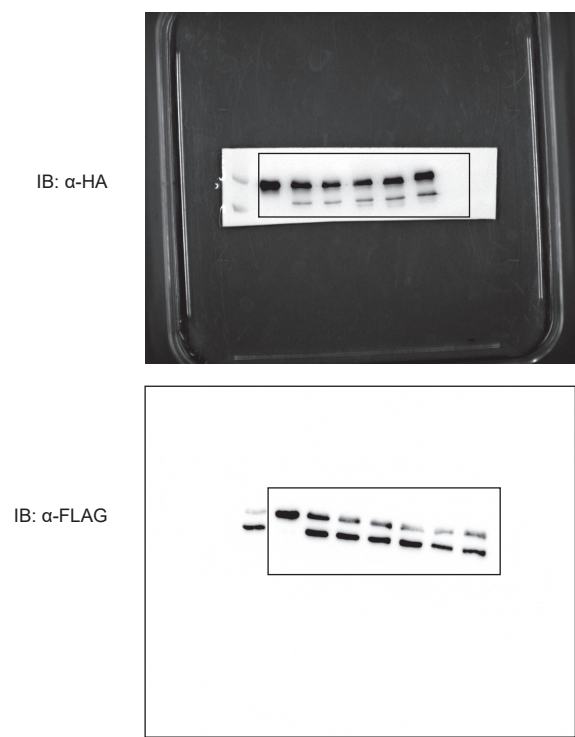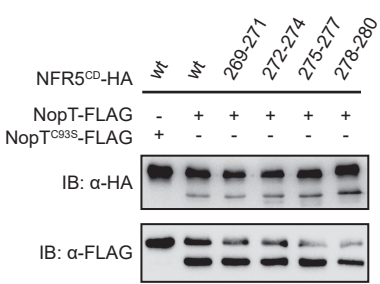

Supplement: Figure 3—figure supplement 4—source data 2. [file elife-97196-fig3-figsupp4-data2.zip › Figure3-S4-SourceData2/Figure3-S4C.pdf]

Figure 3-figure supplement 4A

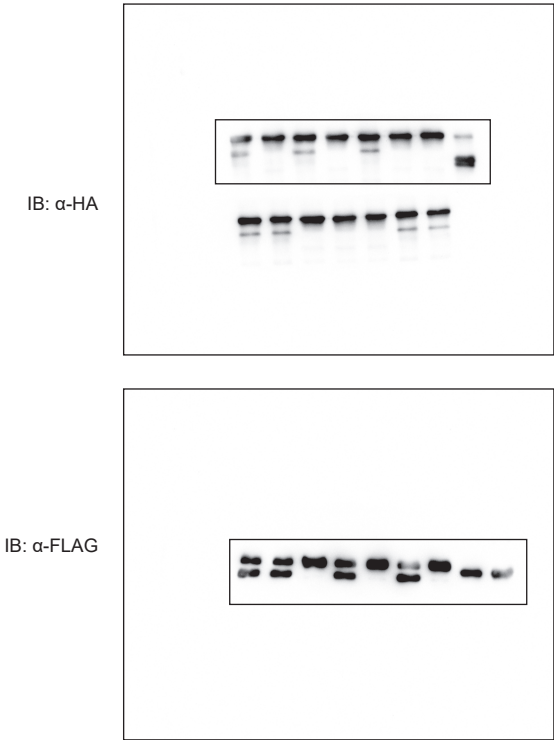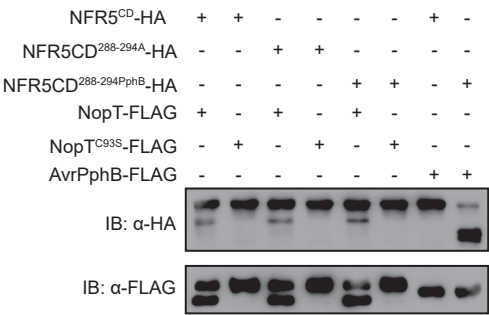

Supplement: Figure 3—figure supplement 4—source data 2. [file elife-97196-fig3-figsupp4-data2.zip › Figure3-S4-SourceData2/Figure3-S4A.pdf]

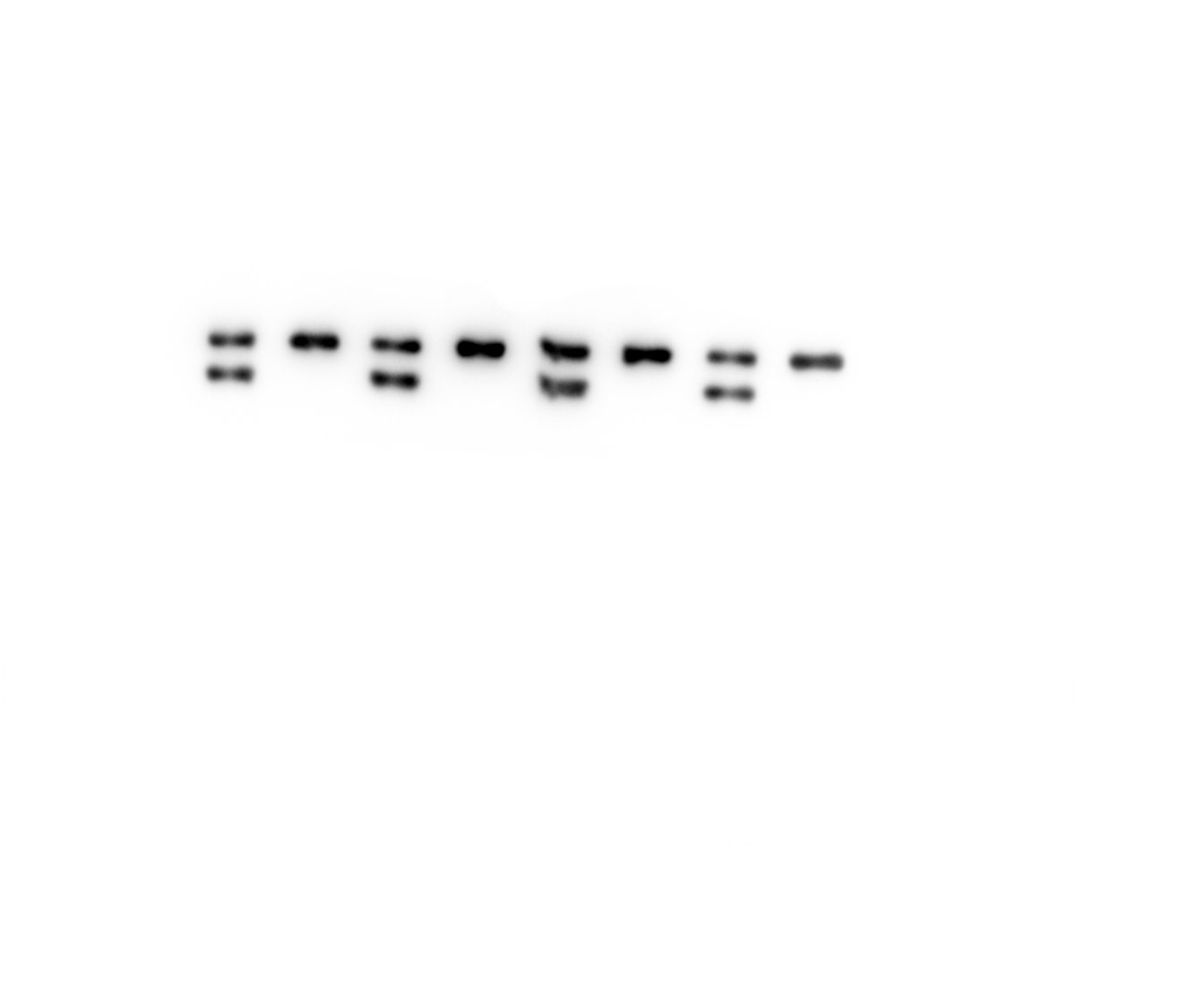

Supplement: Figure 3—figure supplement 6—source data 1. [file elife-97196-fig3-figsupp6-data1.zip › Figure 3-figure supplement 6 a-flag LYK5 LYS11 .tif]

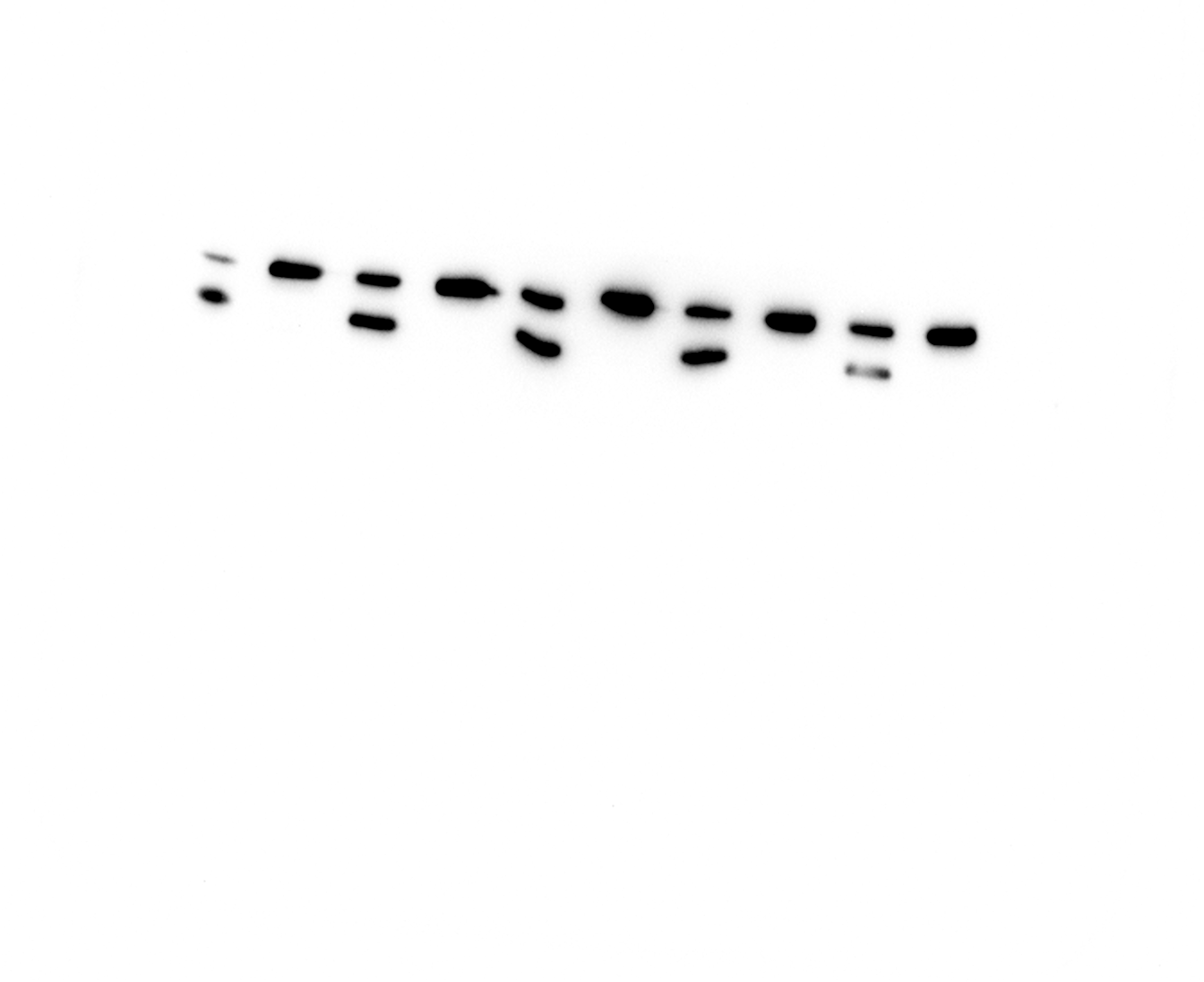

Supplement: Figure 3—figure supplement 6—source data 1. [file elife-97196-fig3-figsupp6-data1.zip › Figure 3-figure supplement 6 a-FLAG NFR1JM.tif]

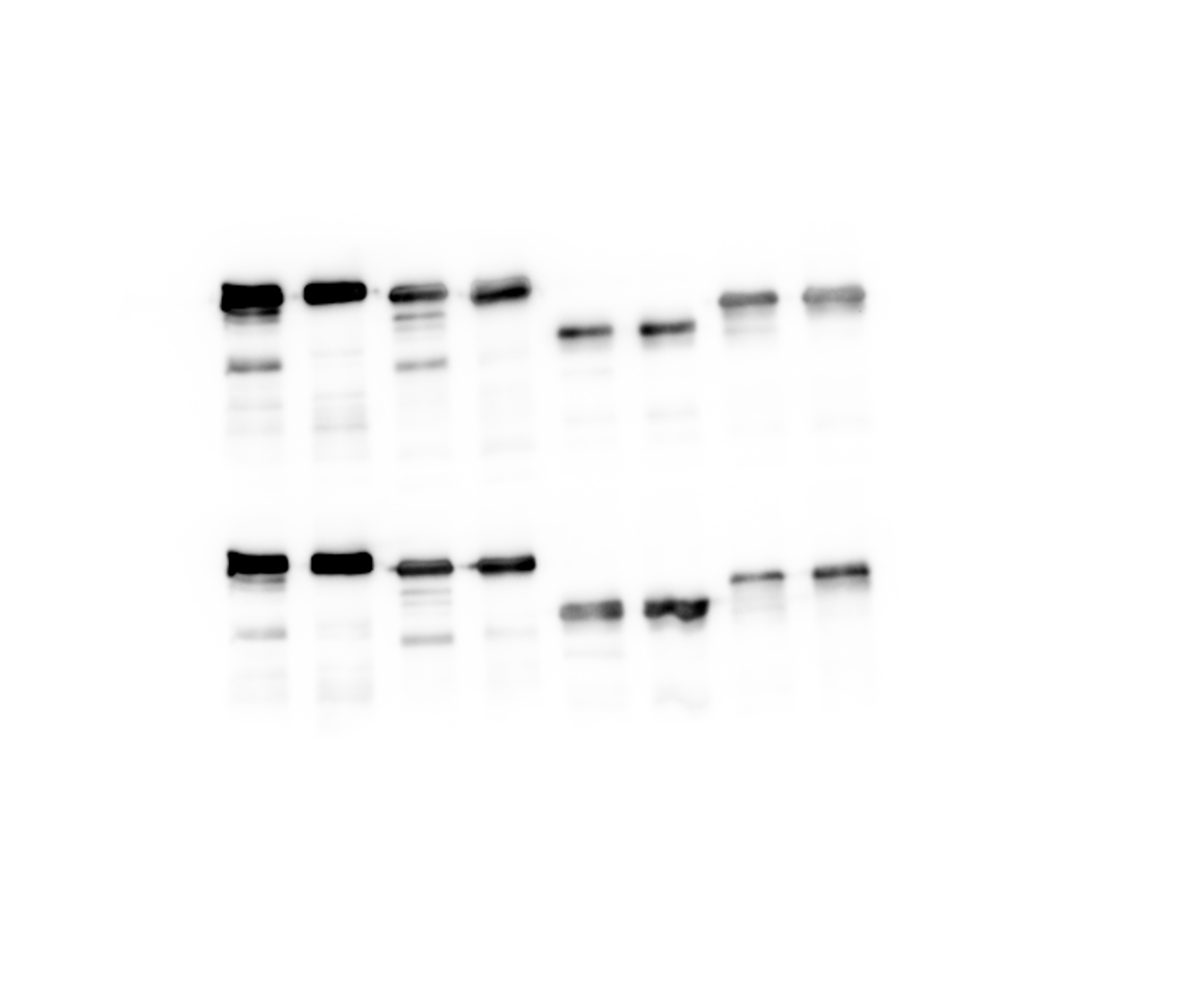

Supplement: Figure 3—figure supplement 6—source data 1. [file elife-97196-fig3-figsupp6-data1.zip › Figure 3-figure supplement 6 a-ha LYK5 LYK5JM.tif]

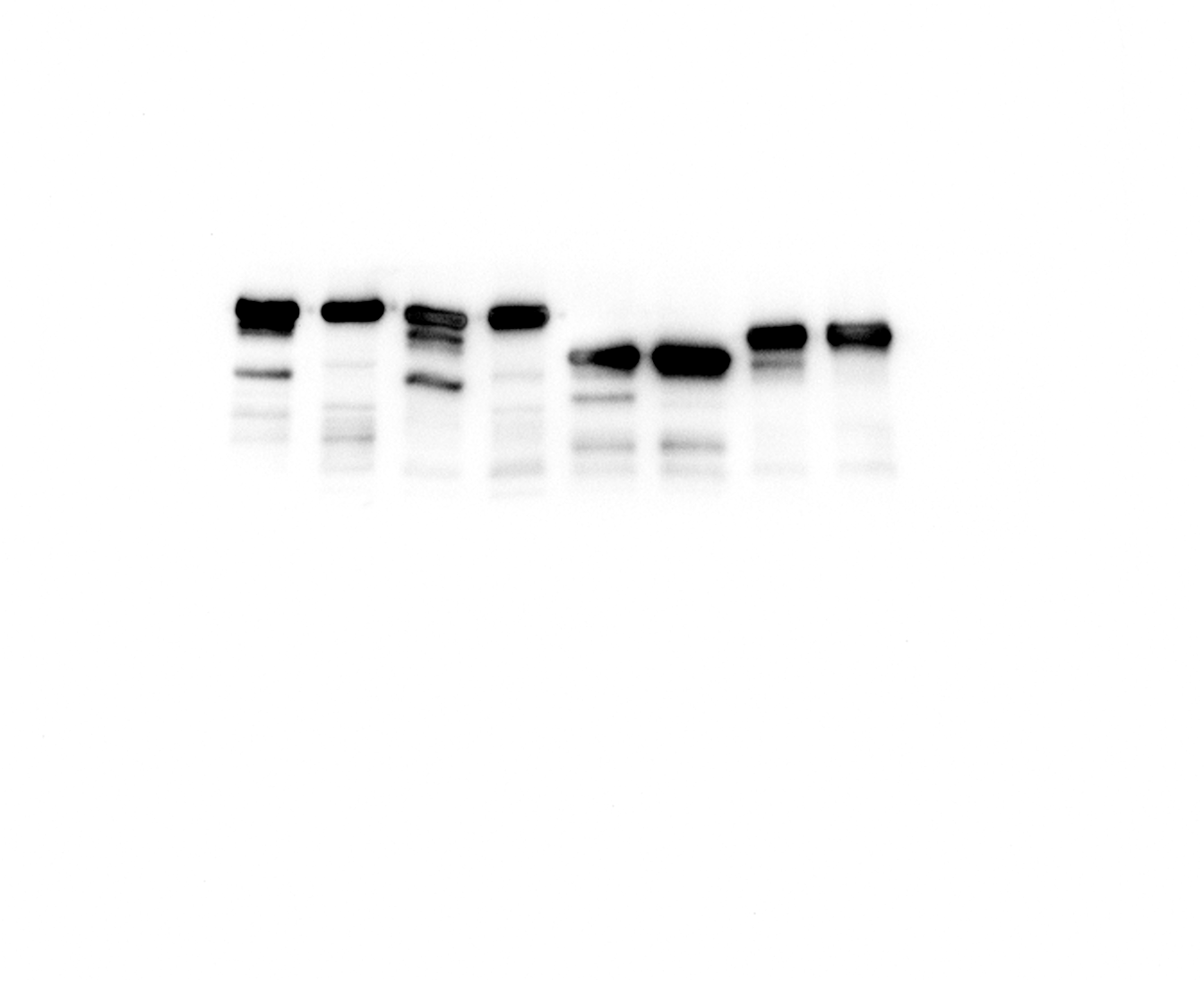

Supplement: Figure 3—figure supplement 6—source data 1. [file elife-97196-fig3-figsupp6-data1.zip › Figure 3-figure supplement 6 a-ha LYS11 LYS11JM.tif]

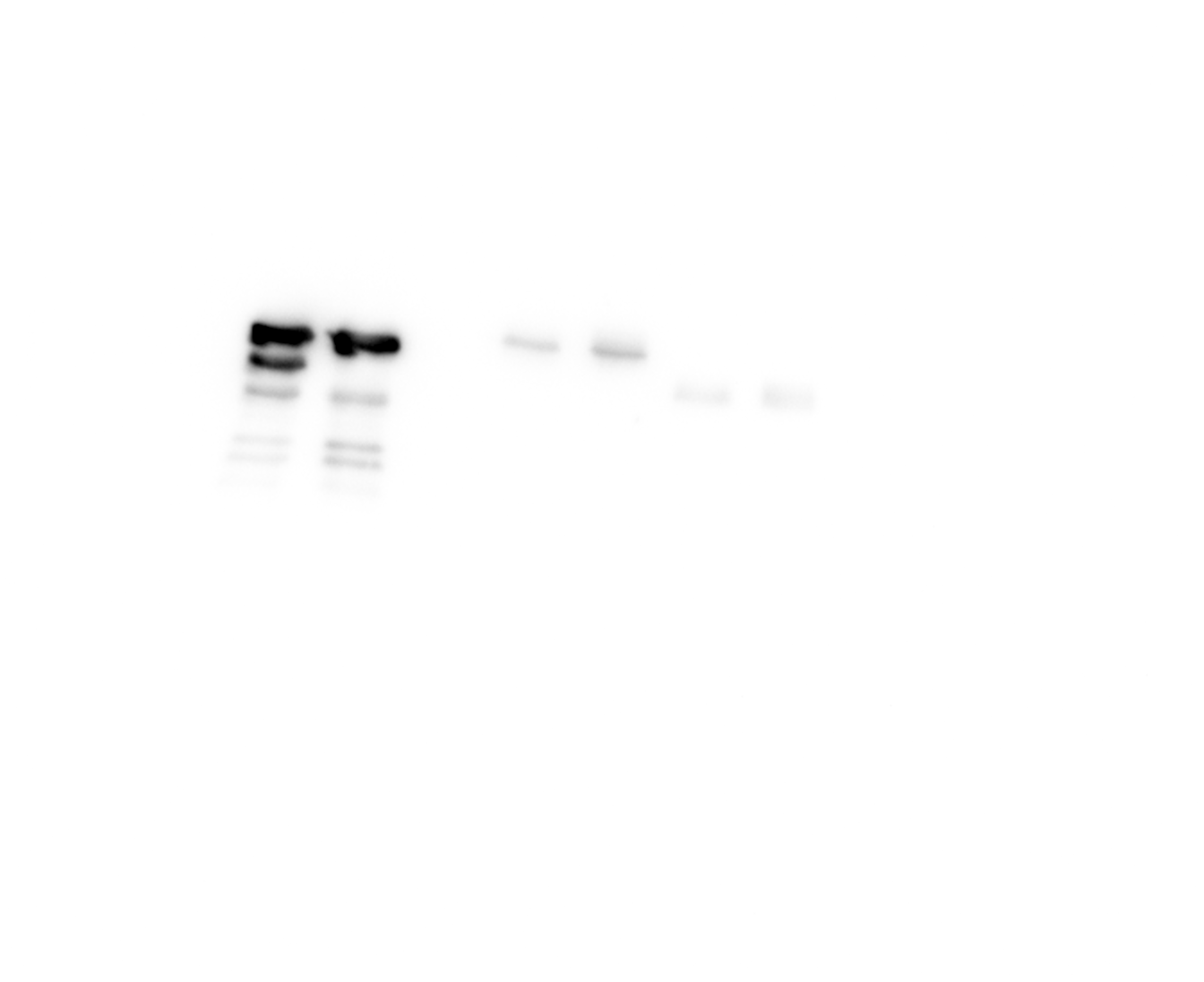

Supplement: Figure 3—figure supplement 6—source data 1. [file elife-97196-fig3-figsupp6-data1.zip › Figure 3-figure supplement 6 a-ha NFR1JM .tif]

Figure 3-figure supplement 6

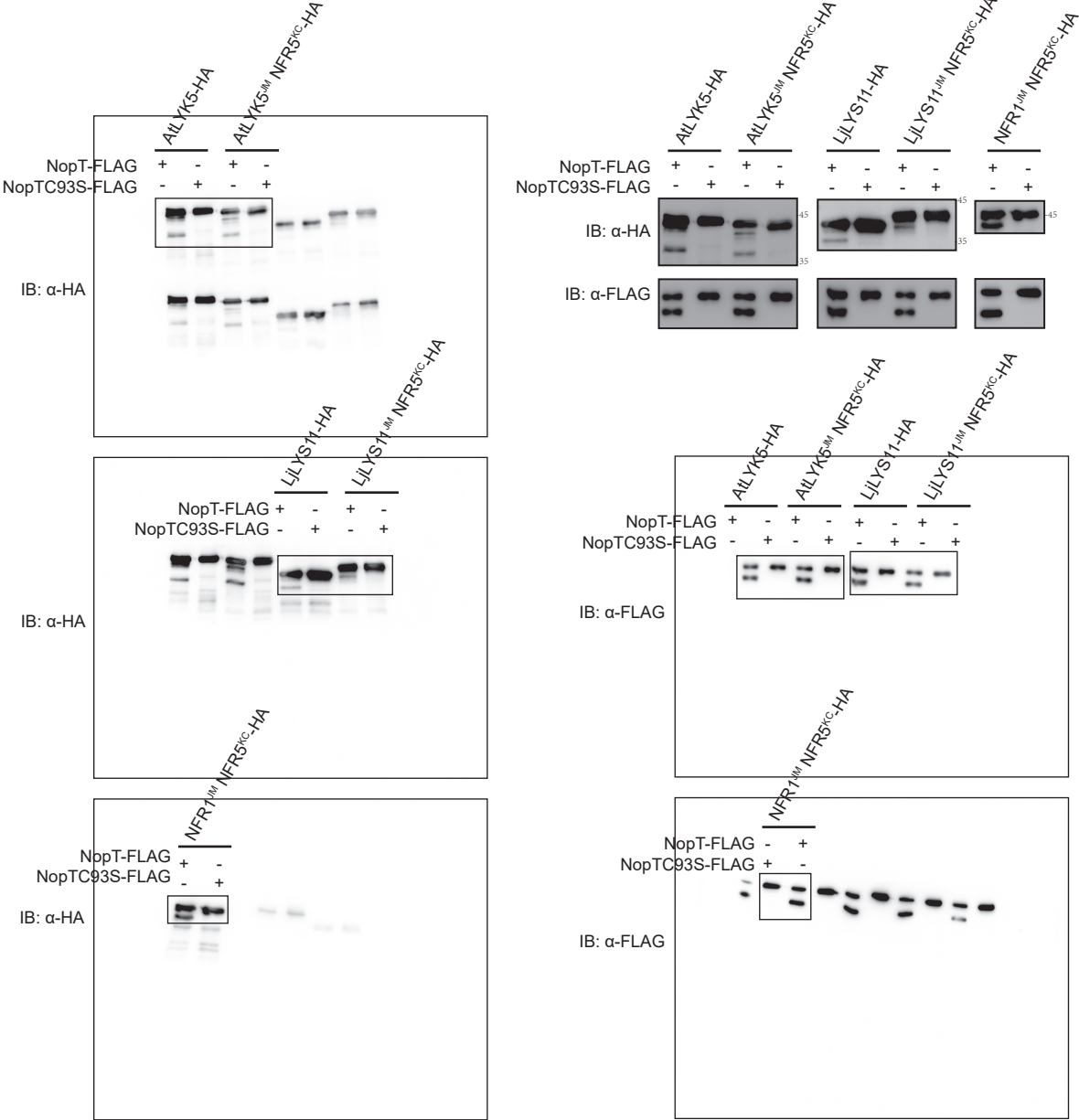

Supplement: Figure 3—figure supplement 6—source data 2. [file elife-97196-fig3-figsupp6-data2.pdf]

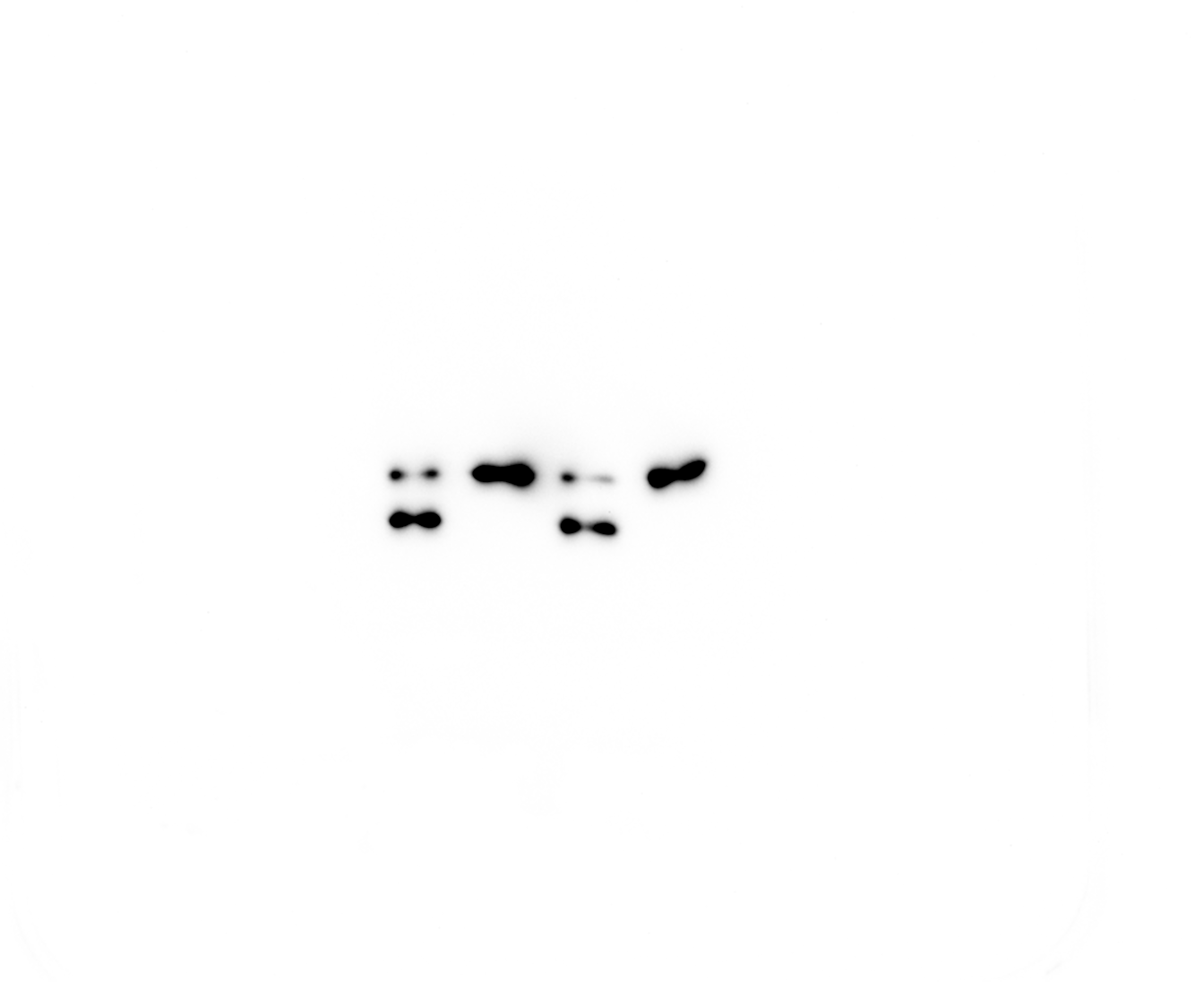

Supplement: Figure 3—figure supplement 7—source data 1. [file elife-97196-fig3-figsupp7-data1.zip › Figure 3-figure supplement 7 a-flag ft.tif]

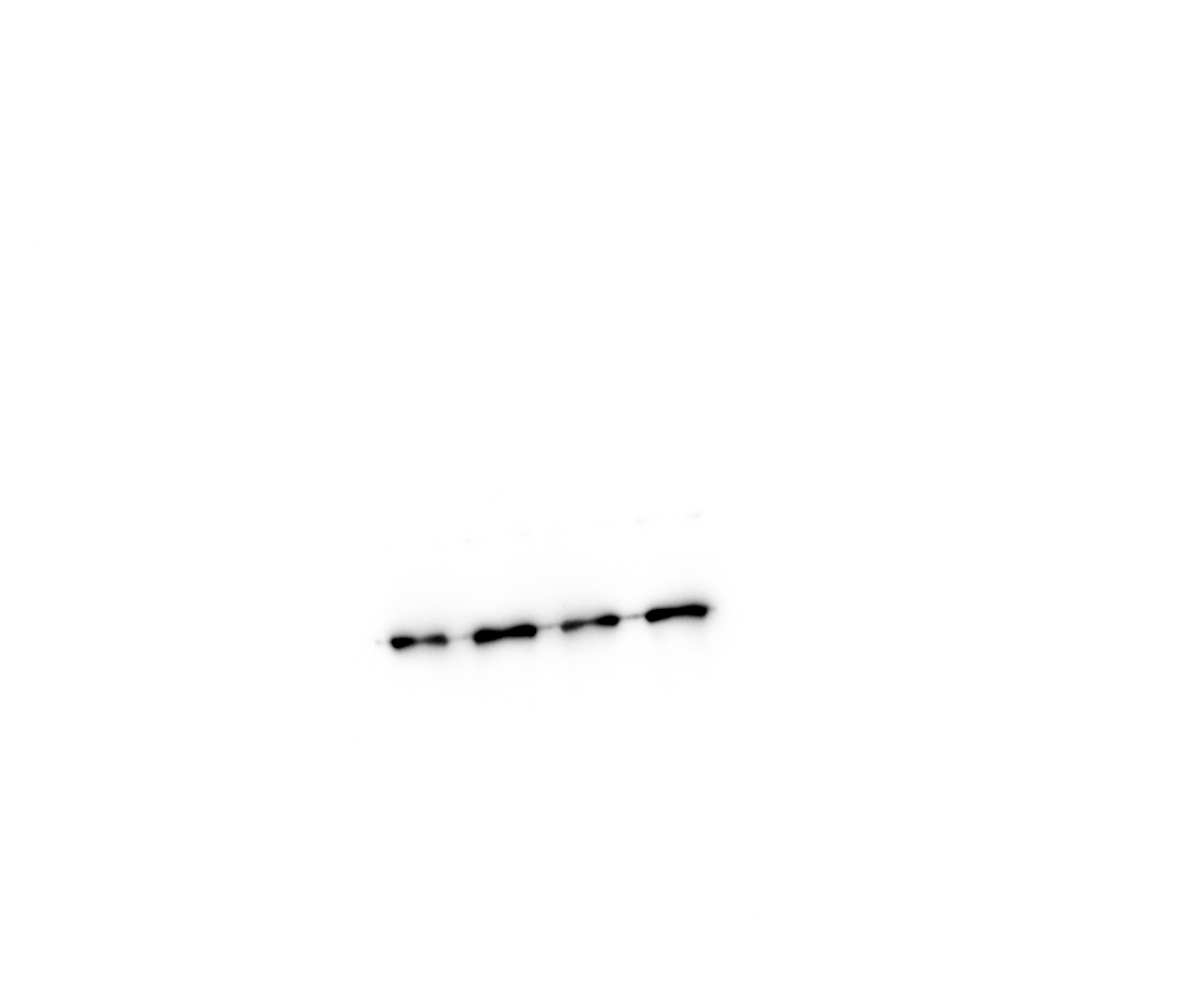

Supplement: Figure 3—figure supplement 7—source data 1. [file elife-97196-fig3-figsupp7-data1.zip › Figure 3-figure supplement 7 in a-gfp.tif]

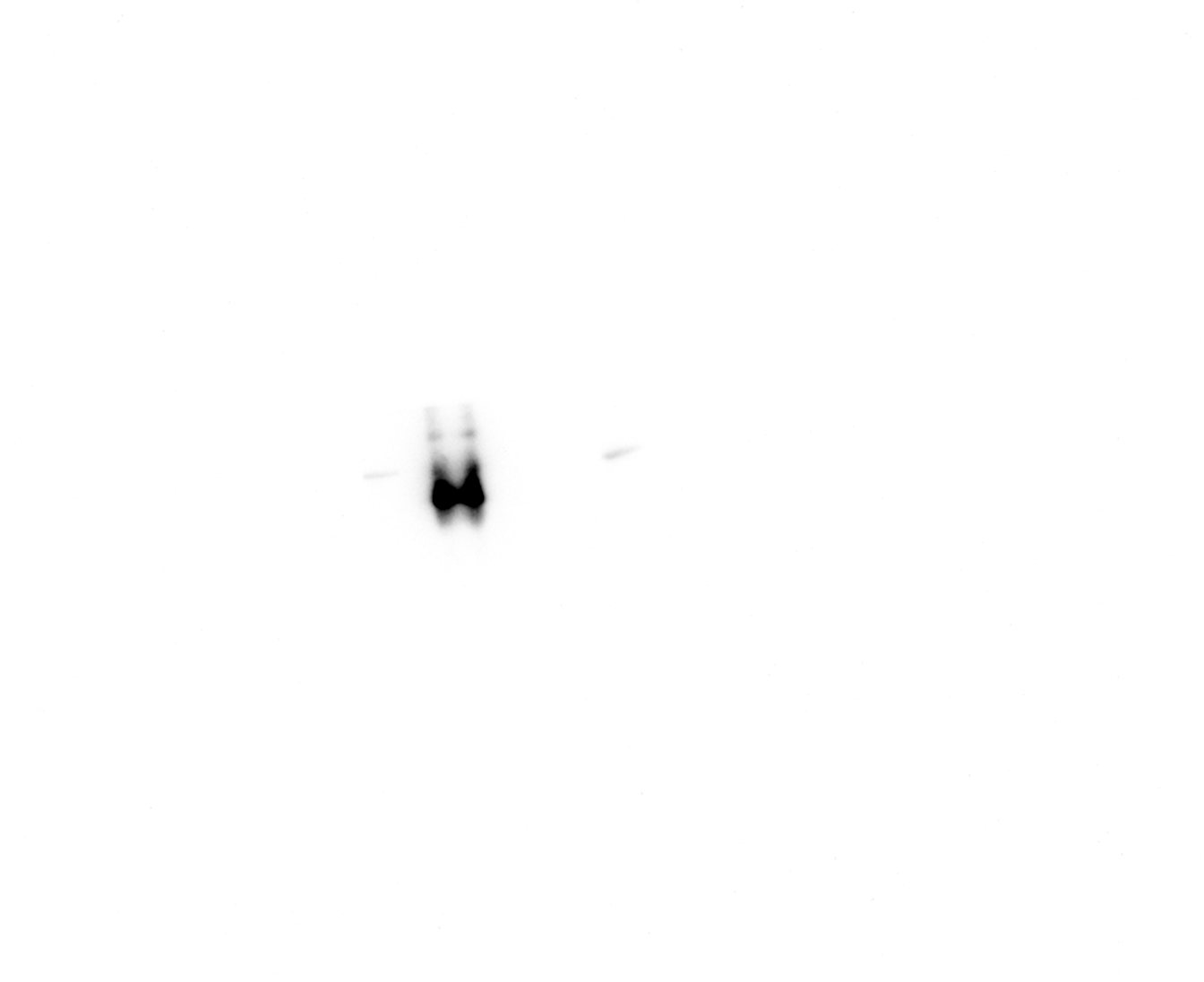

Supplement: Figure 3—figure supplement 7—source data 1. [file elife-97196-fig3-figsupp7-data1.zip › Figure 3-figure supplement 7 a-gfp ft.tif]

Figure 3-figure supplement 7

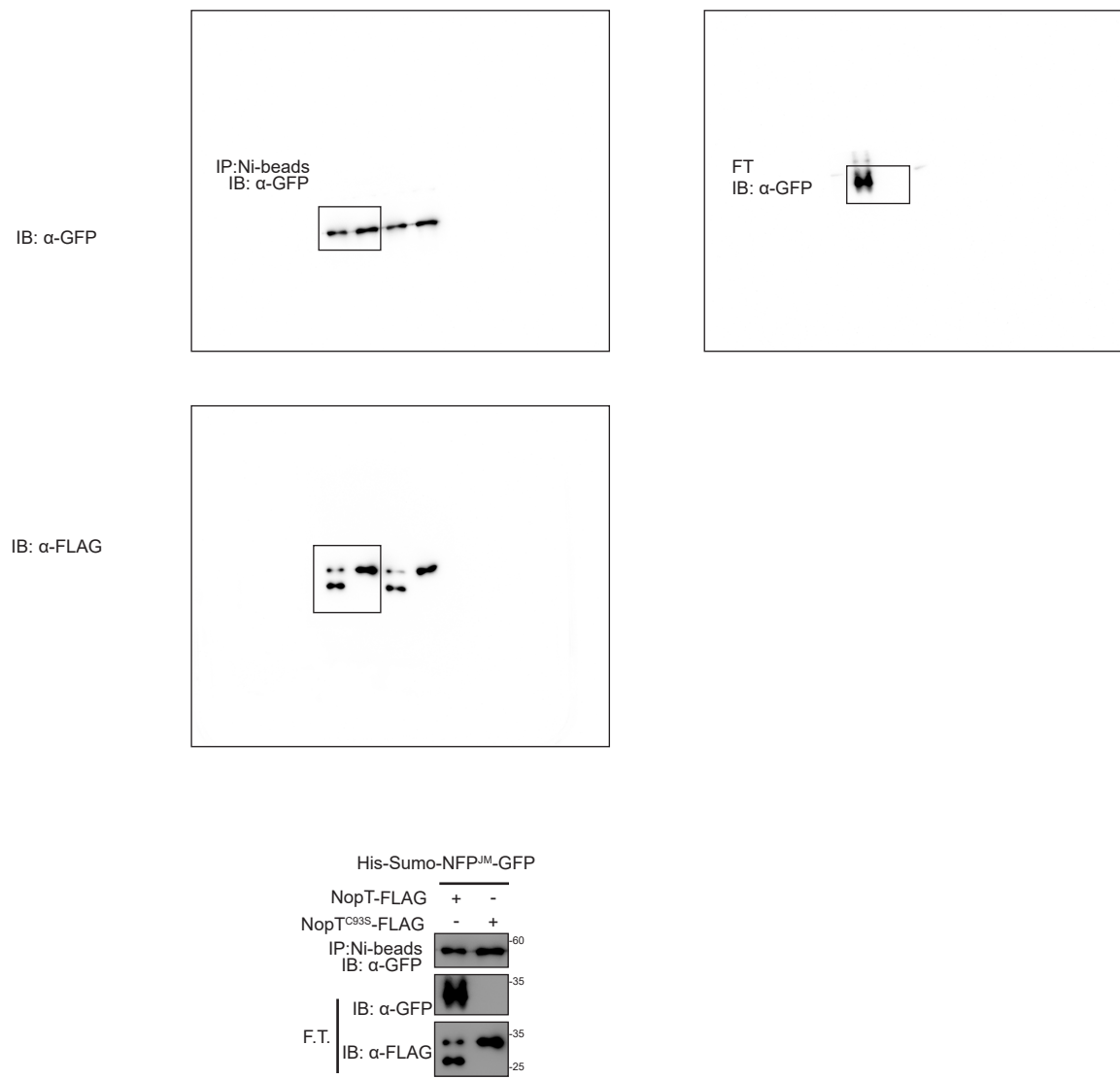

Supplement: Figure 3—figure supplement 7—source data 2. [file elife-97196-fig3-figsupp7-data2.pdf]

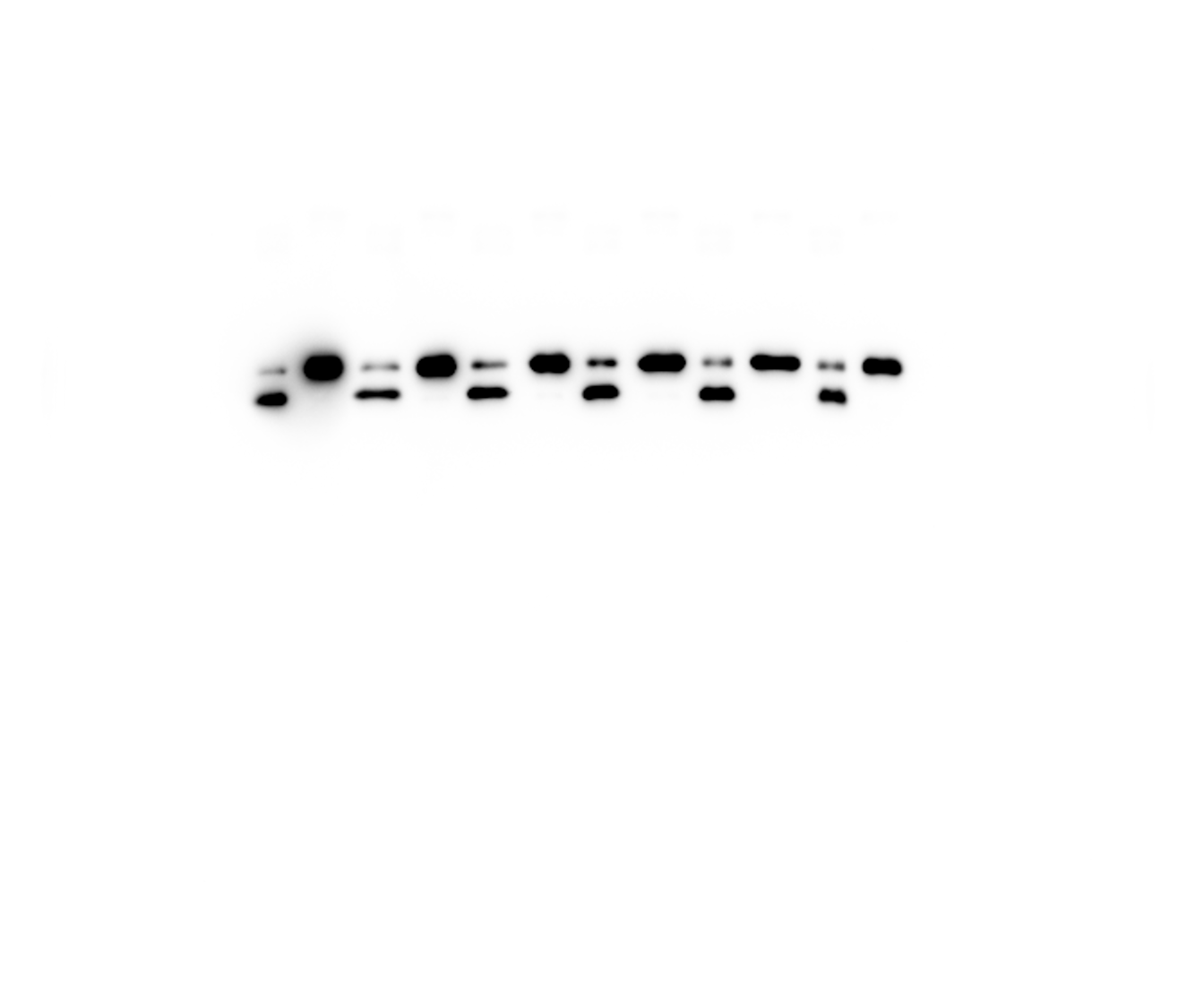

Supplement: Figure 3—figure supplement 8—source data 1. [file elife-97196-fig3-figsupp8-data1.zip › Figure 3-figure supplement 8 a-flag.tif]

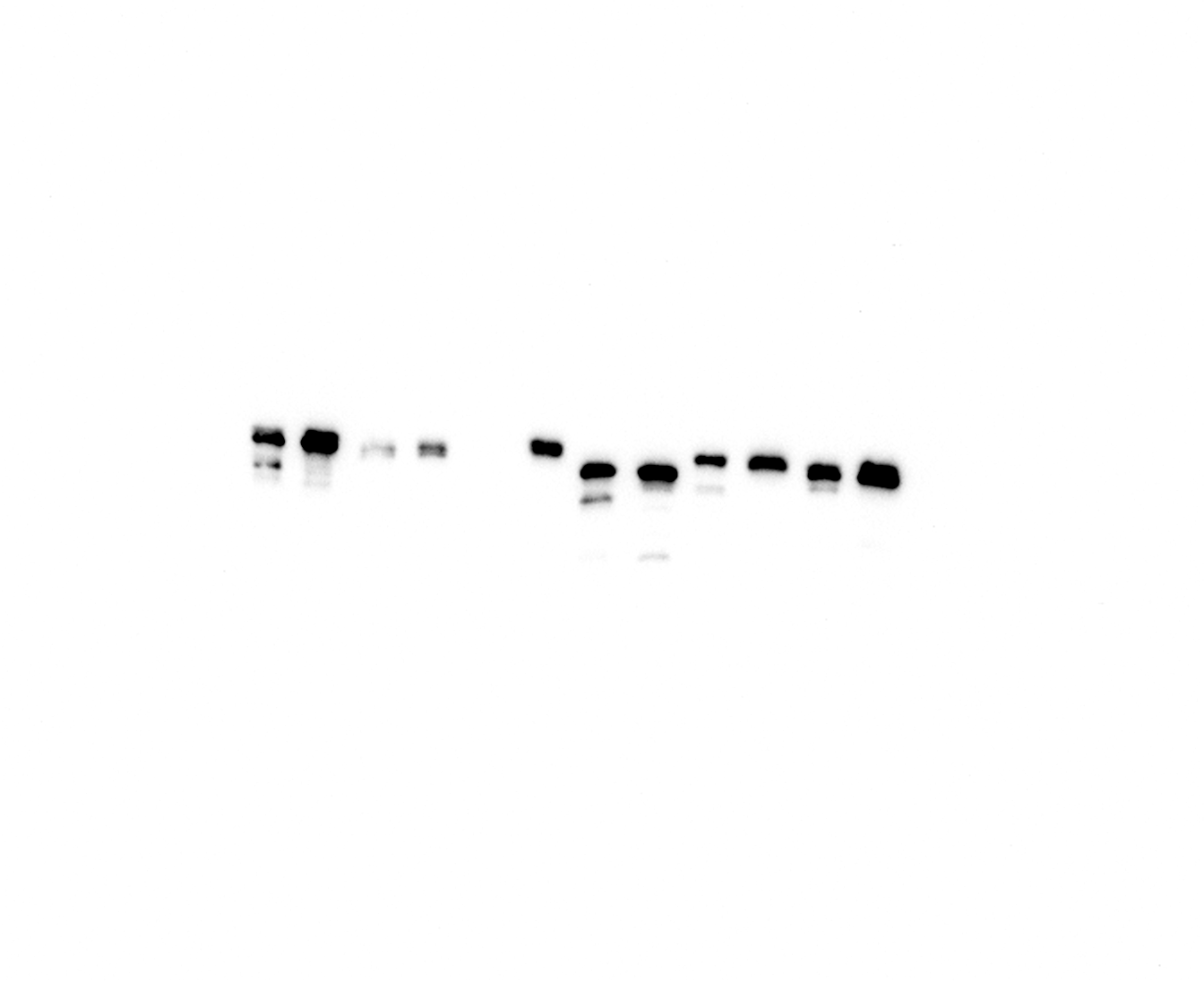

Supplement: Figure 3—figure supplement 8—source data 1. [file elife-97196-fig3-figsupp8-data1.zip › Figure 3-figure supplement 8 a-ha .tif]

Figure 3-figure supplement 8

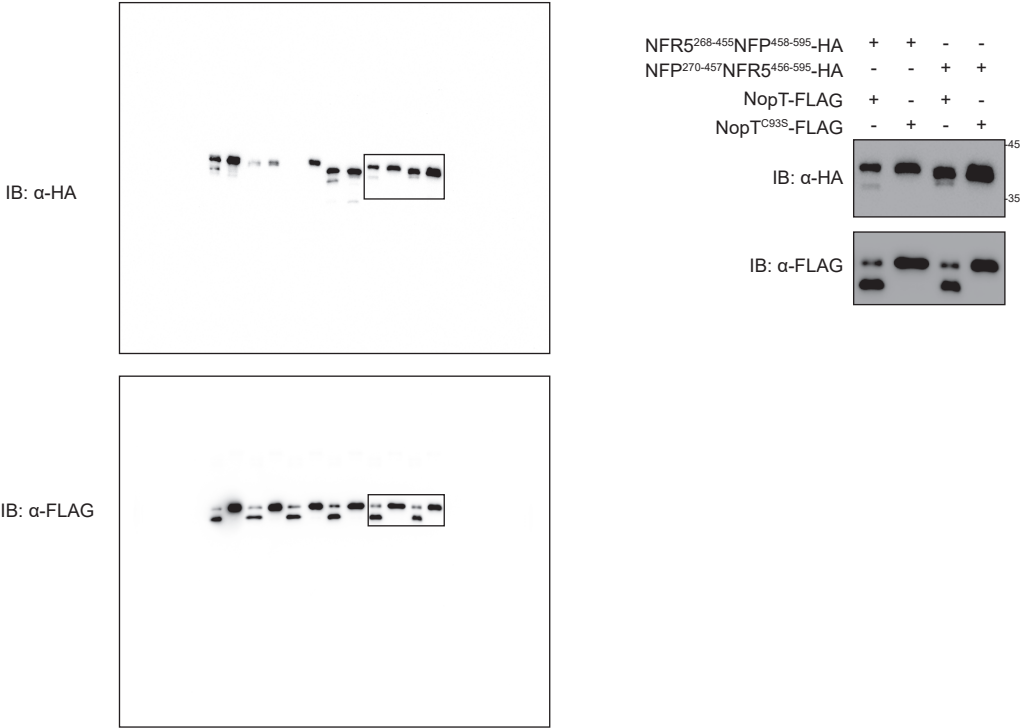

Supplement: Figure 3—figure supplement 8—source data 2. [file elife-97196-fig3-figsupp8-data2.pdf]

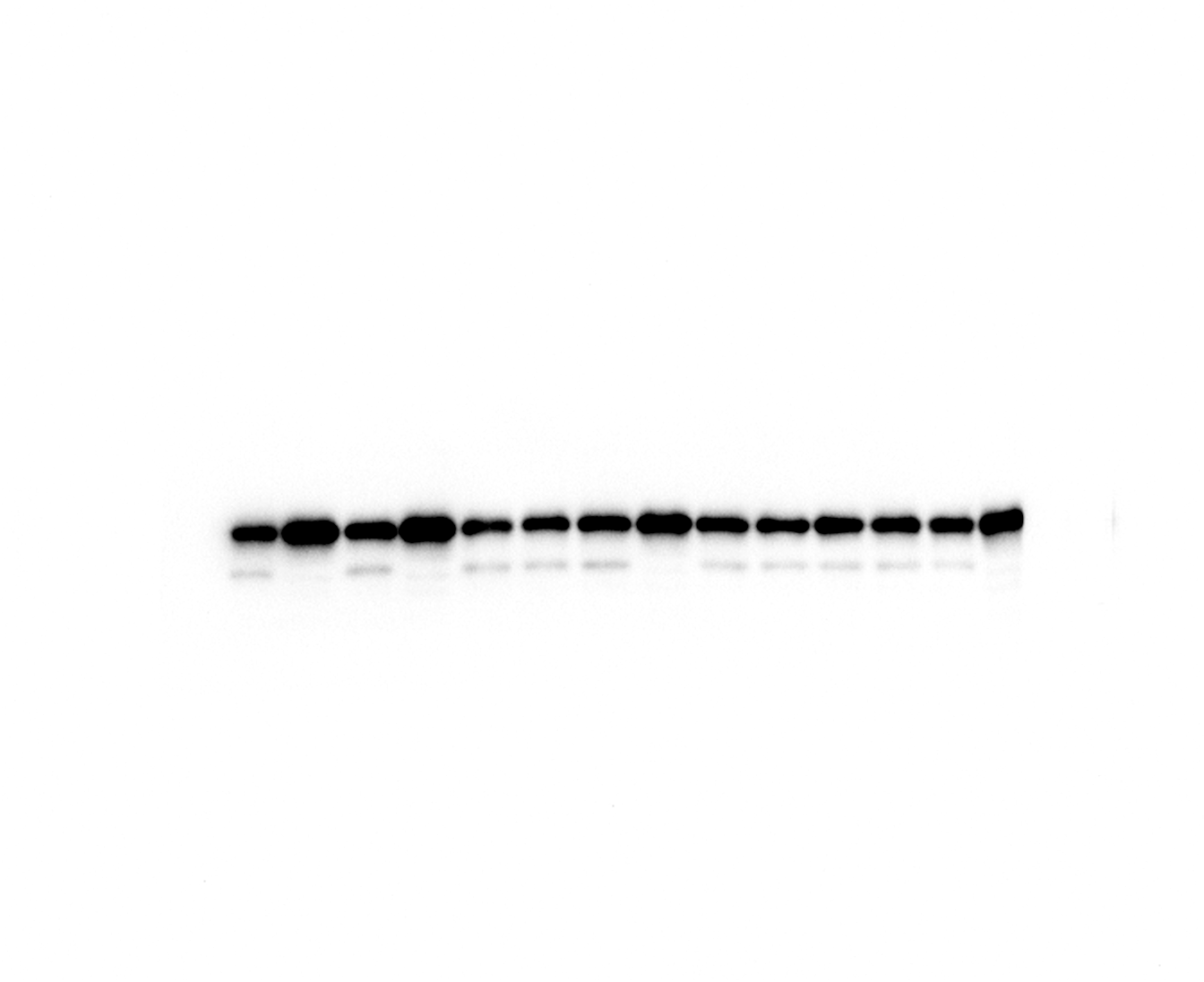

Supplement: Figure 4—source data 1. [file elife-97196-fig4-data1.zip › Figure4-SourceData1/Figure4D/Fig4D A-HA .tif]

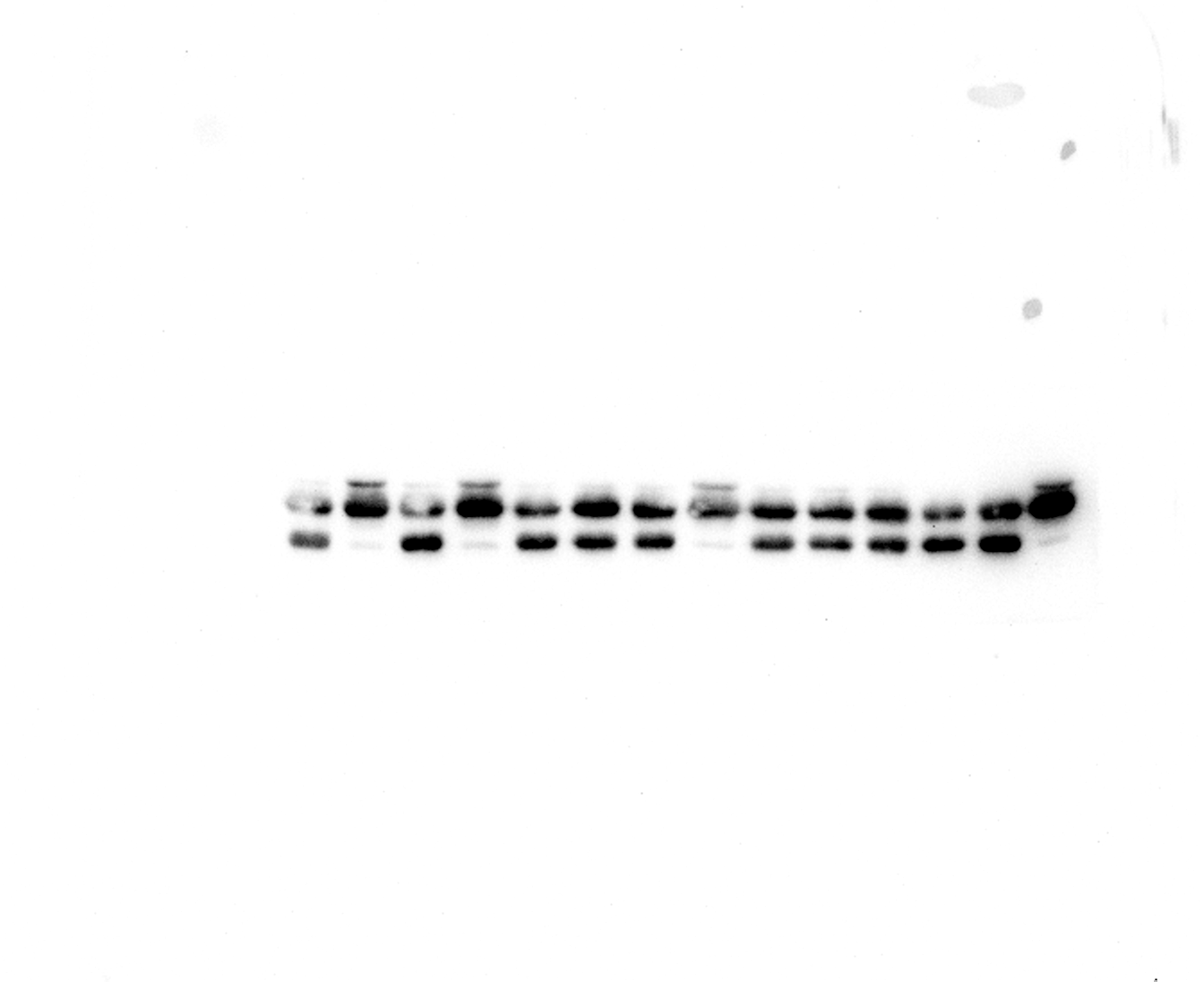

Supplement: Figure 4—source data 1. [file elife-97196-fig4-data1.zip › Figure4-SourceData1/Figure4D/Fig4D A-FLAg.tif]

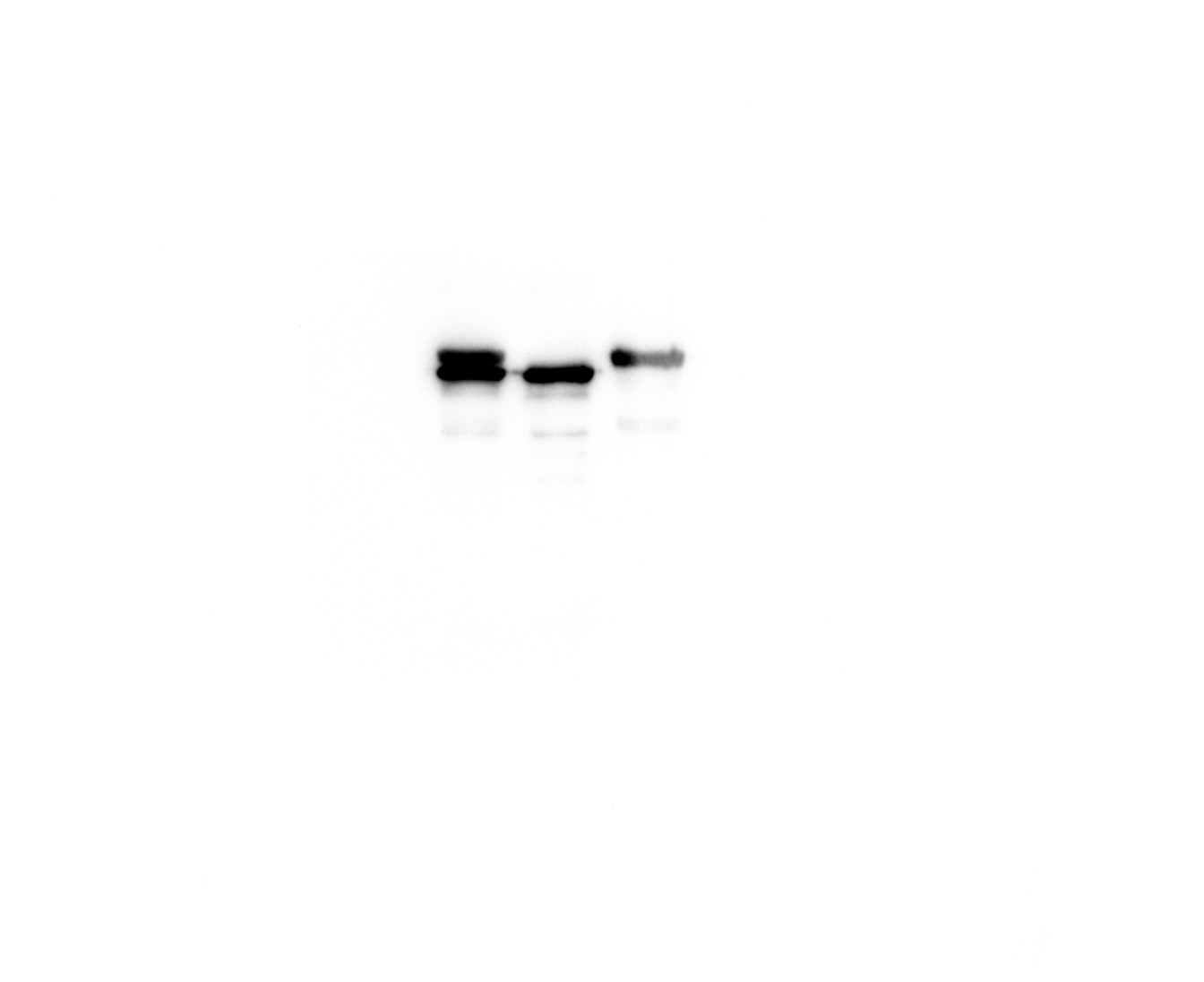

Supplement: Figure 4—source data 1. [file elife-97196-fig4-data1.zip › Figure4-SourceData1/Figure4C/fig4C a-myc.tif]

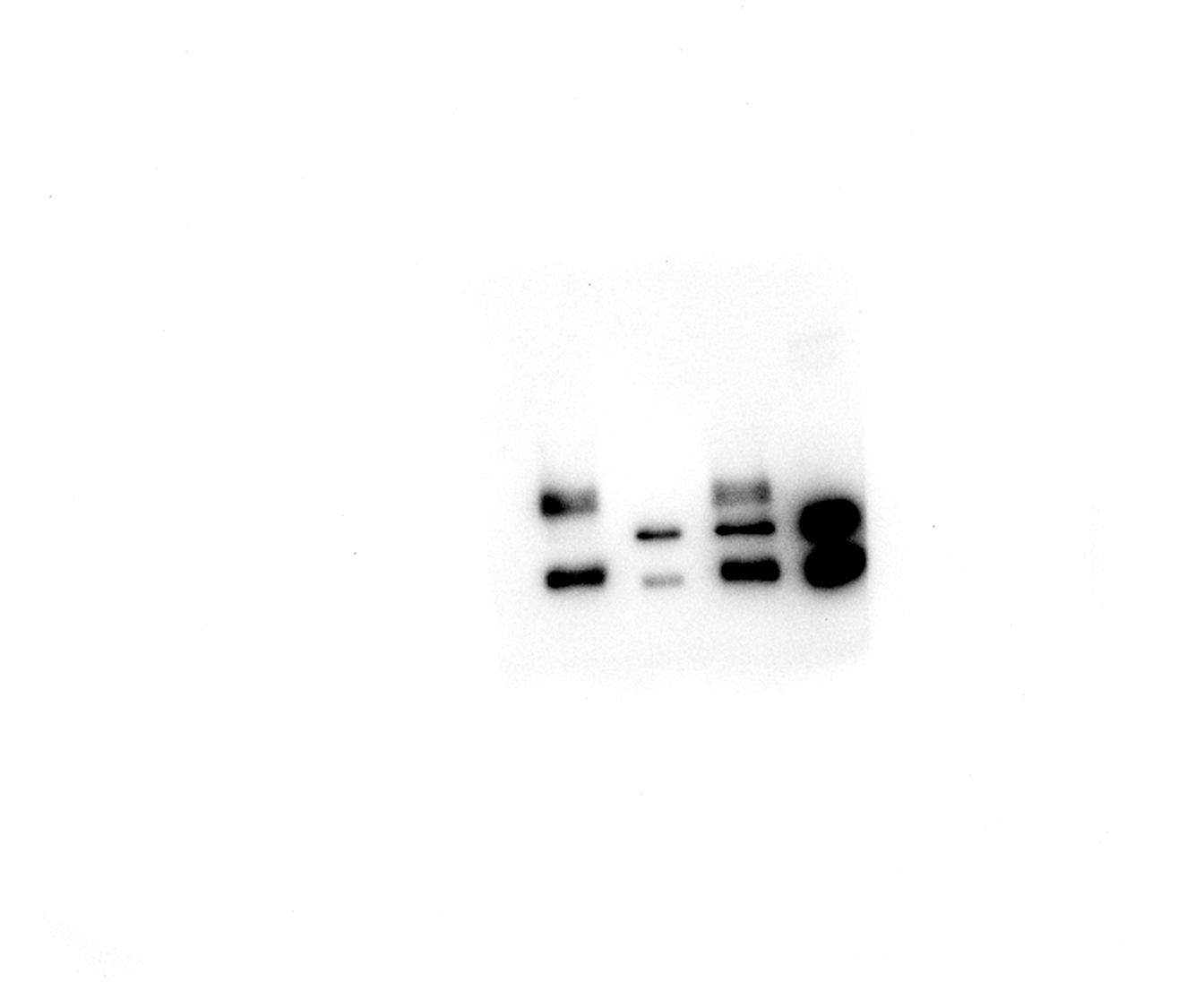

Supplement: Figure 4—source data 1. [file elife-97196-fig4-data1.zip › Figure4-SourceData1/Figure4C/fig4C a-FLAG .tif]

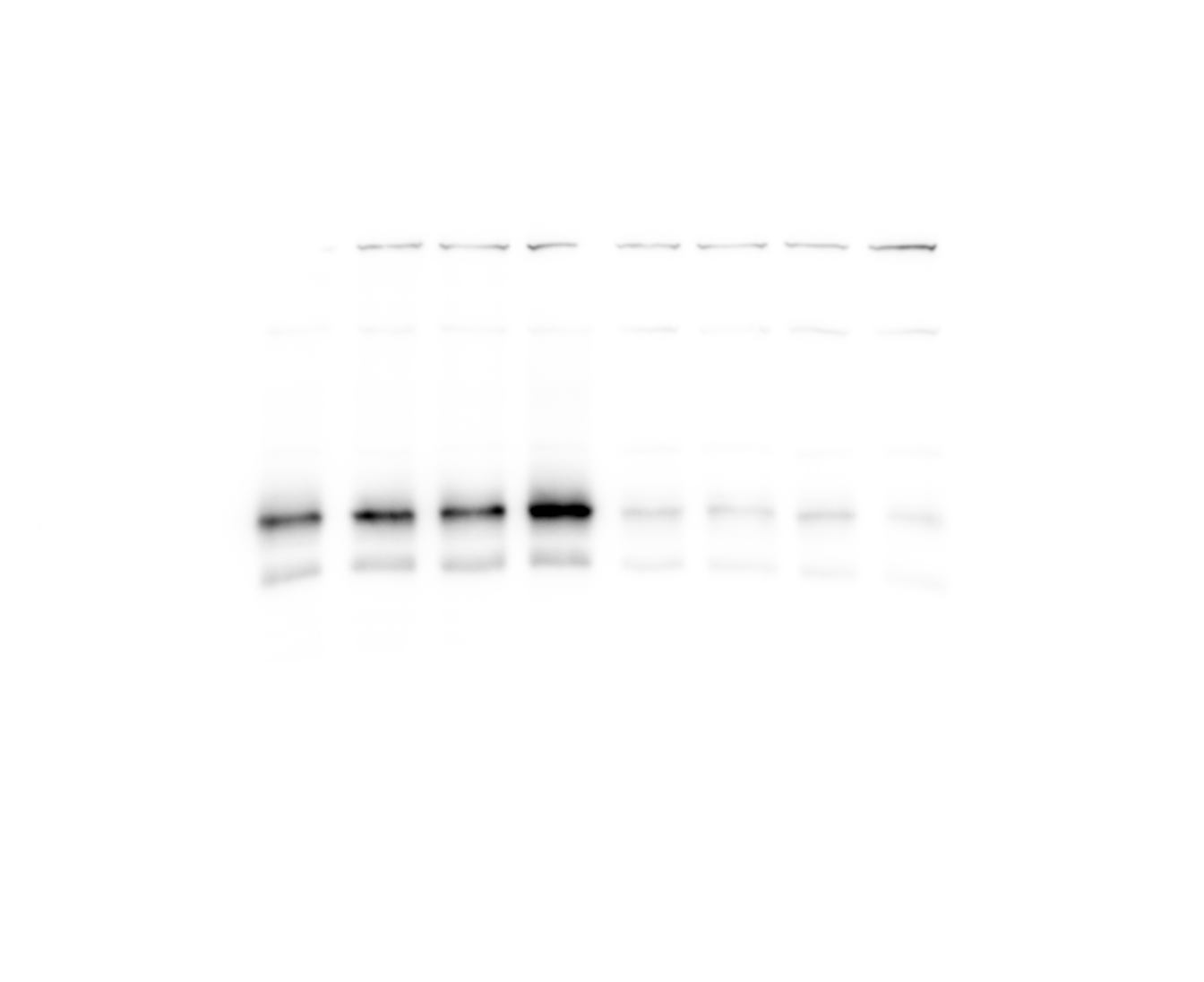

Supplement: Figure 4—source data 1. [file elife-97196-fig4-data1.zip › Figure4-SourceData1/Figure4A/Fig4A a-flag.tif]

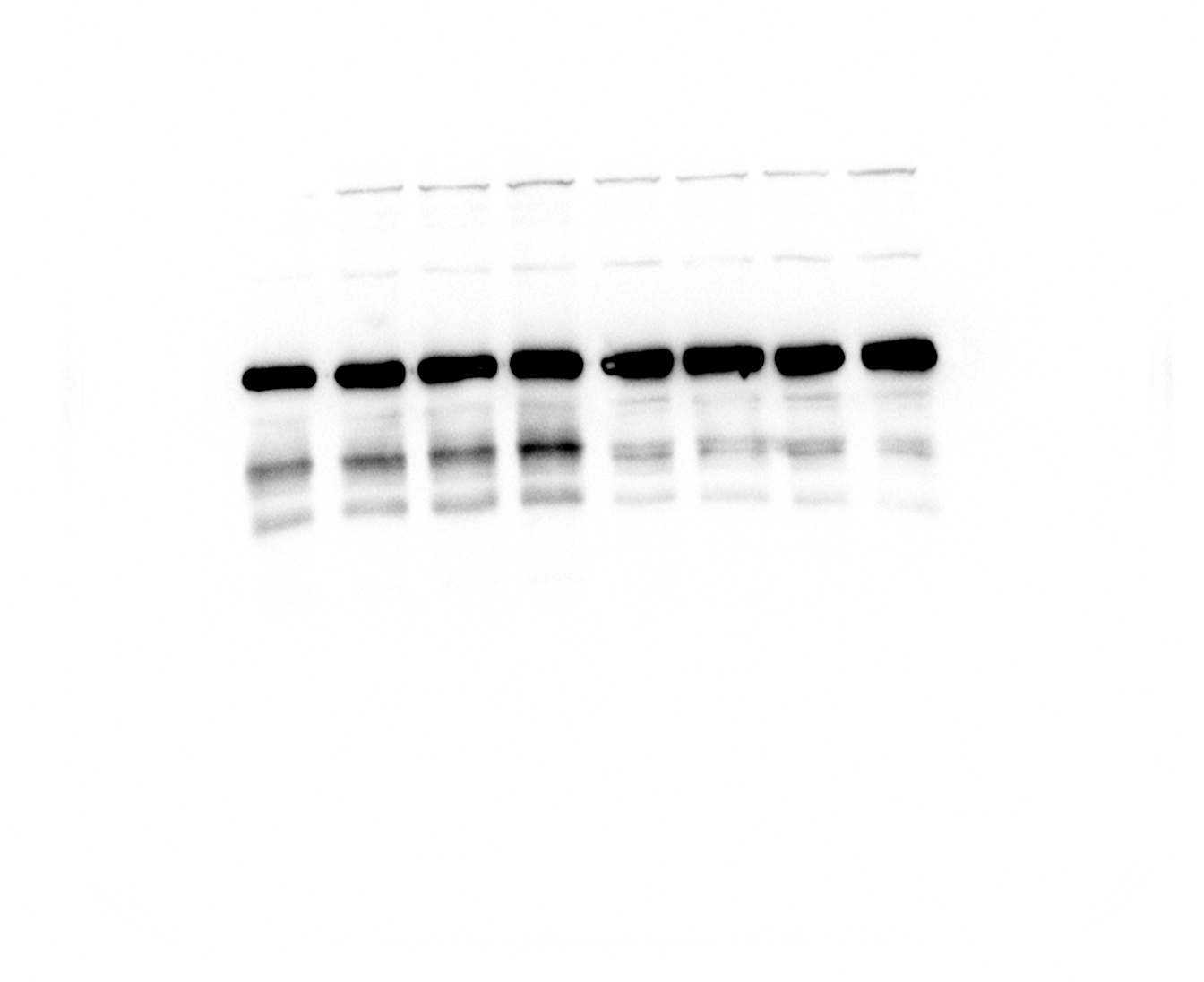

Supplement: Figure 4—source data 1. [file elife-97196-fig4-data1.zip › Figure4-SourceData1/Figure4A/Fig4A a-actin.tif]

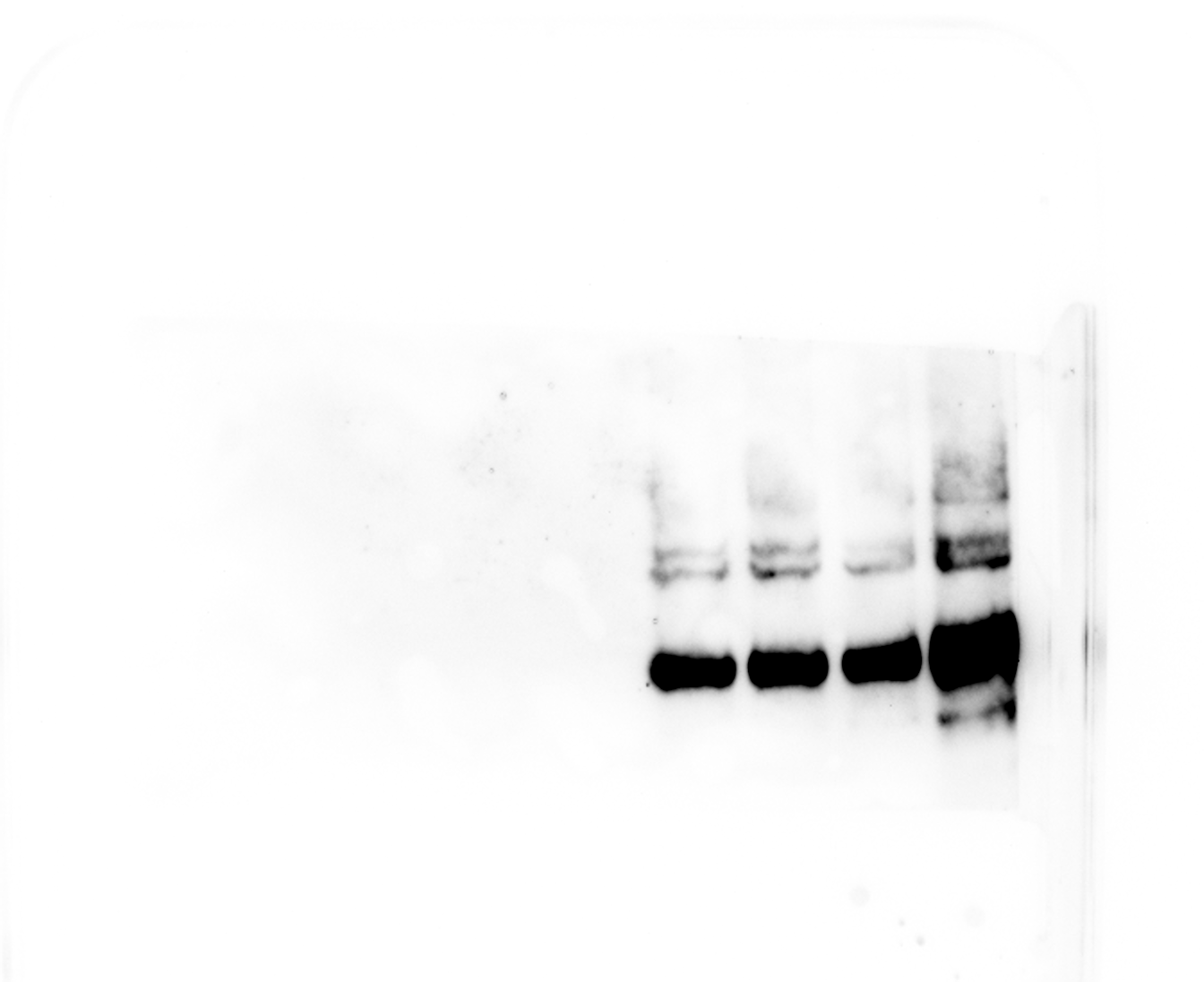

Supplement: Figure 4—source data 1. [file elife-97196-fig4-data1.zip › Figure4-SourceData1/Figure4A/Fig4A Zn phos-tag a-flag plant root.tif]

Fig. 4A

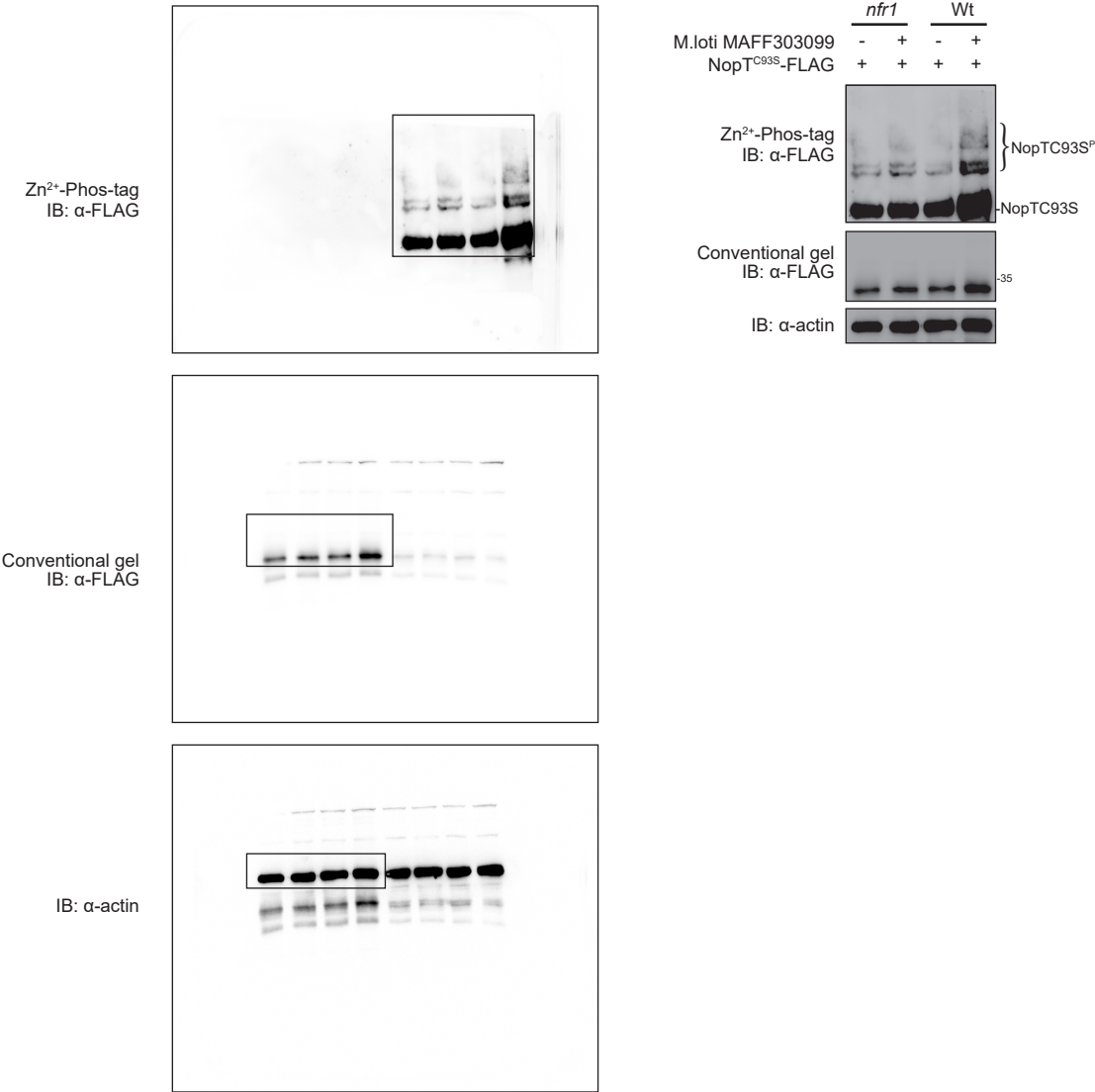

Supplement: Figure 4—source data 2. [file elife-97196-fig4-data2.zip › Figure4-SourceData2/Figure4A.pdf]

Fig. 4C

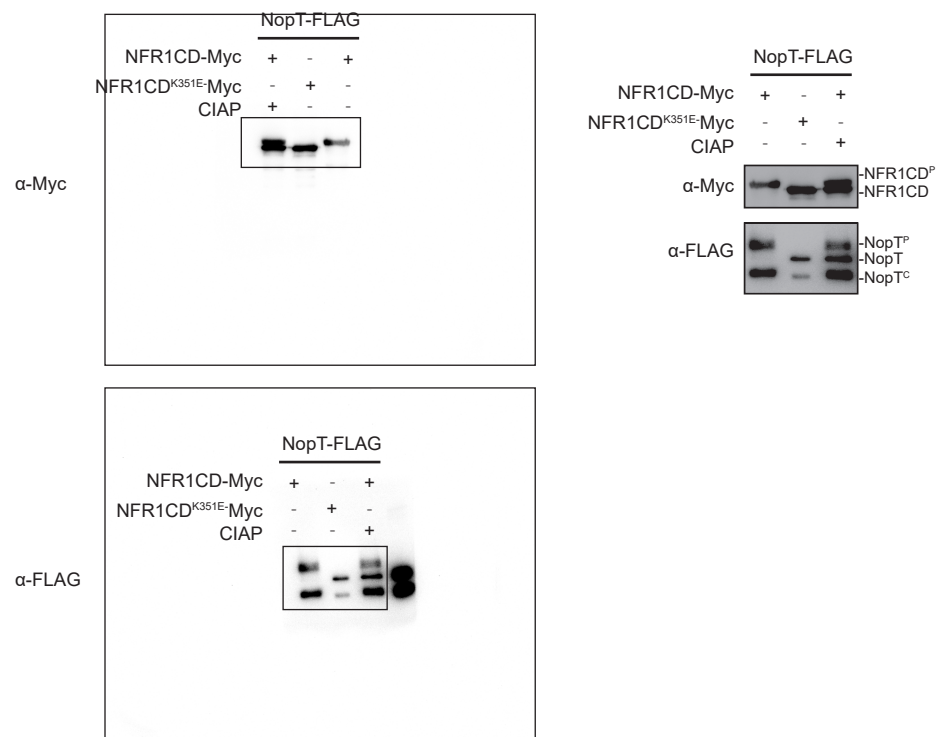

Supplement: Figure 4—source data 2. [file elife-97196-fig4-data2.zip › Figure4-SourceData2/Figure4C.pdf]

Fig. 4D

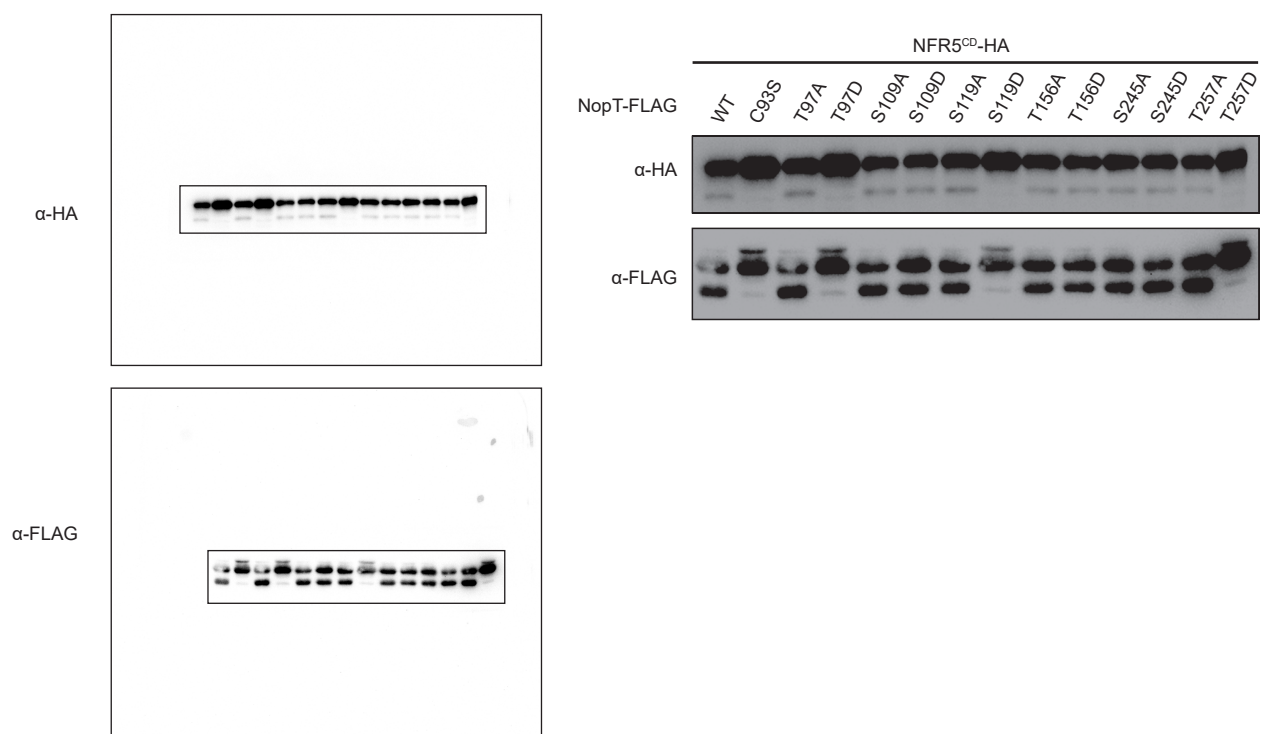

Supplement: Figure 4—source data 2. [file elife-97196-fig4-data2.zip › Figure4-SourceData2/Figure4D.pdf]

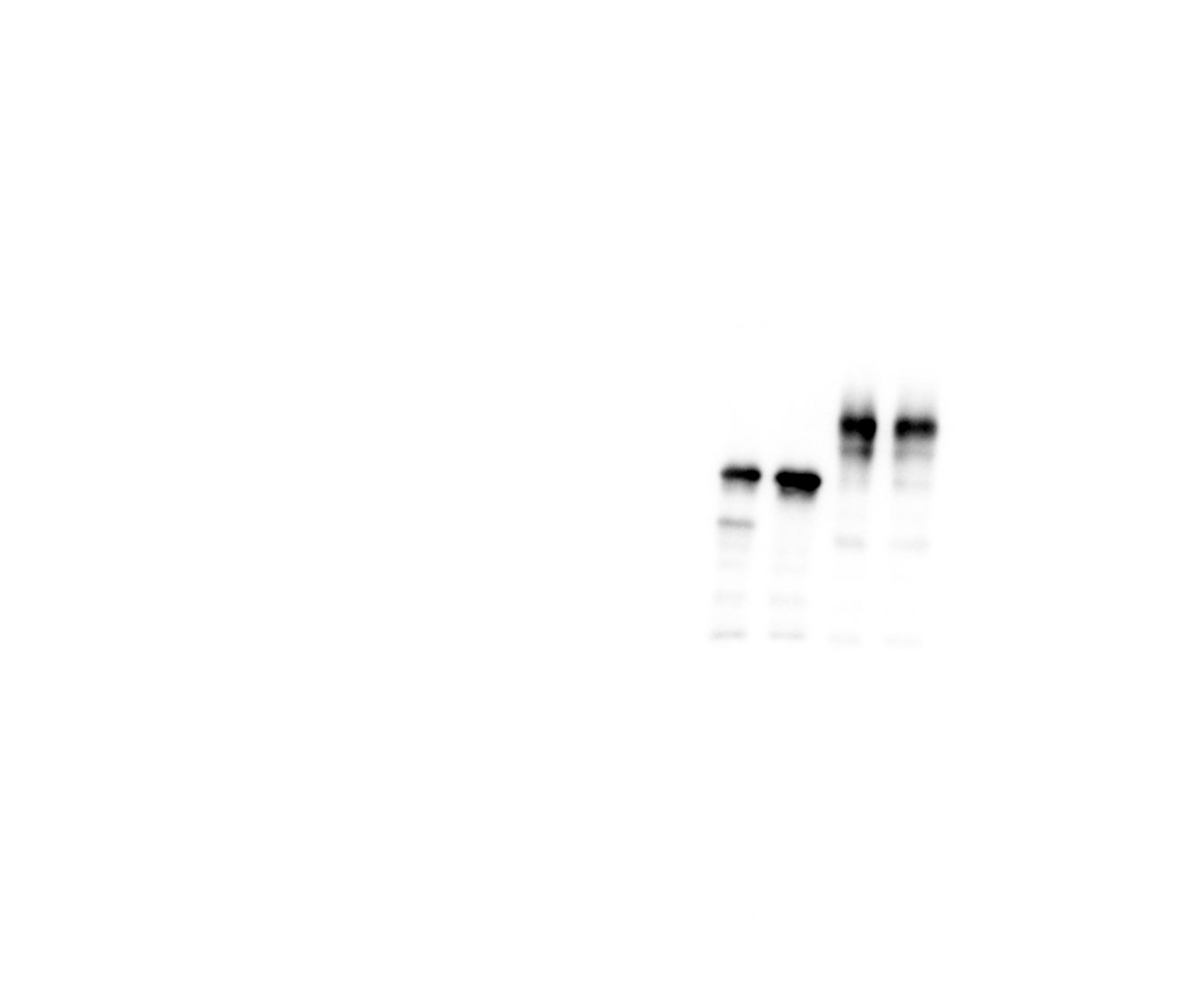

Supplement: Figure 4—figure supplement 1—source data 1. [file elife-97196-fig4-figsupp1-data1.zip › Figure 4-figure supplement 1 a-ha .tif]

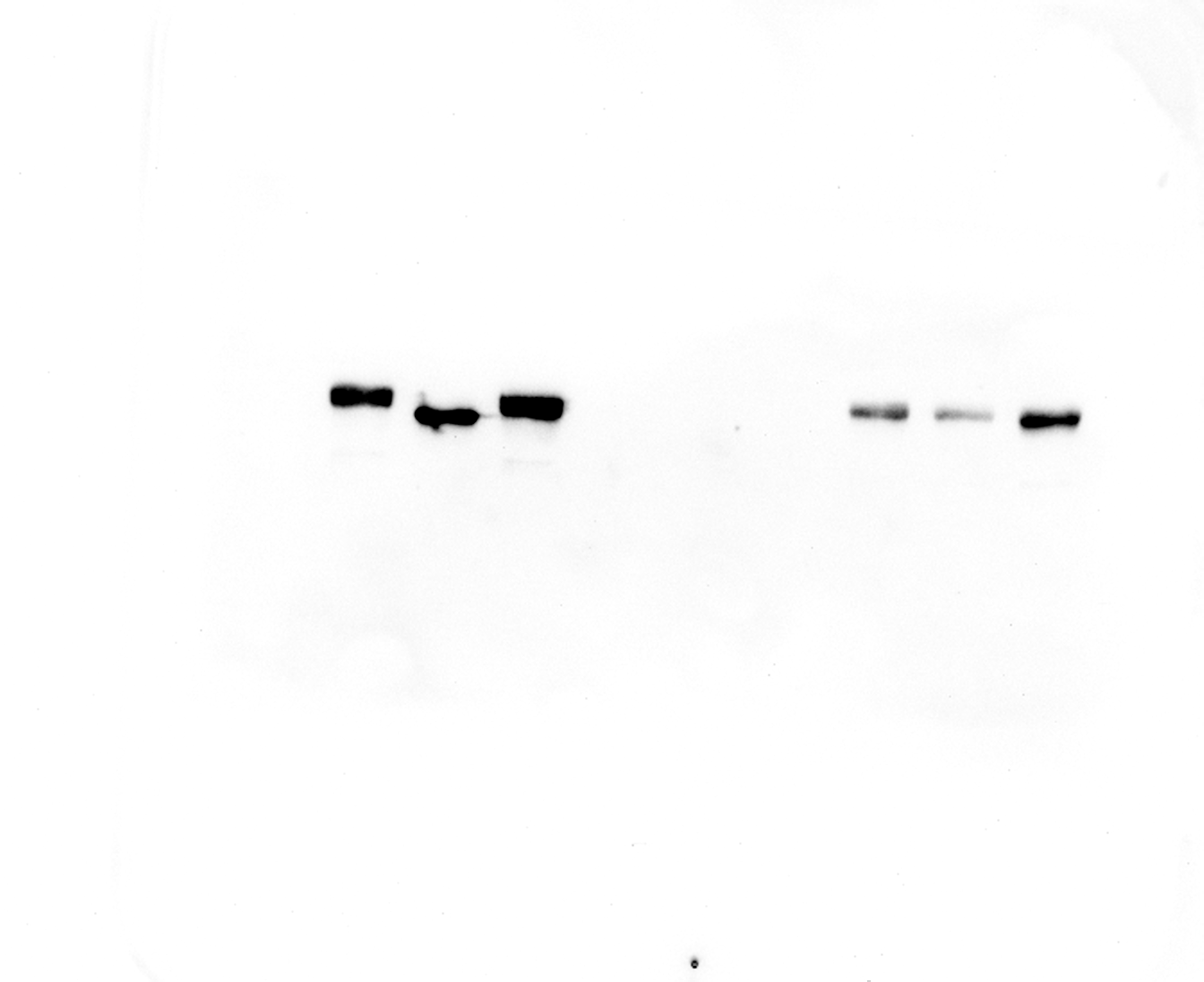

Supplement: Figure 4—figure supplement 1—source data 1. [file elife-97196-fig4-figsupp1-data1.zip › Figure 4-figure supplement 1 a-myc.tif]
