# Supplementary figures and images for: The rhizobial effector NopT targets Nod factor receptors to regulate symbiosis in Lotus japonicus (part 2 of 2)
Source: eLife. 2025 Apr 4;13:RP97196. doi: 10.7554/eLife.97196 (PMC11970910; doi:10.7554/eLife.97196)

Figure 4-figure supplement 1

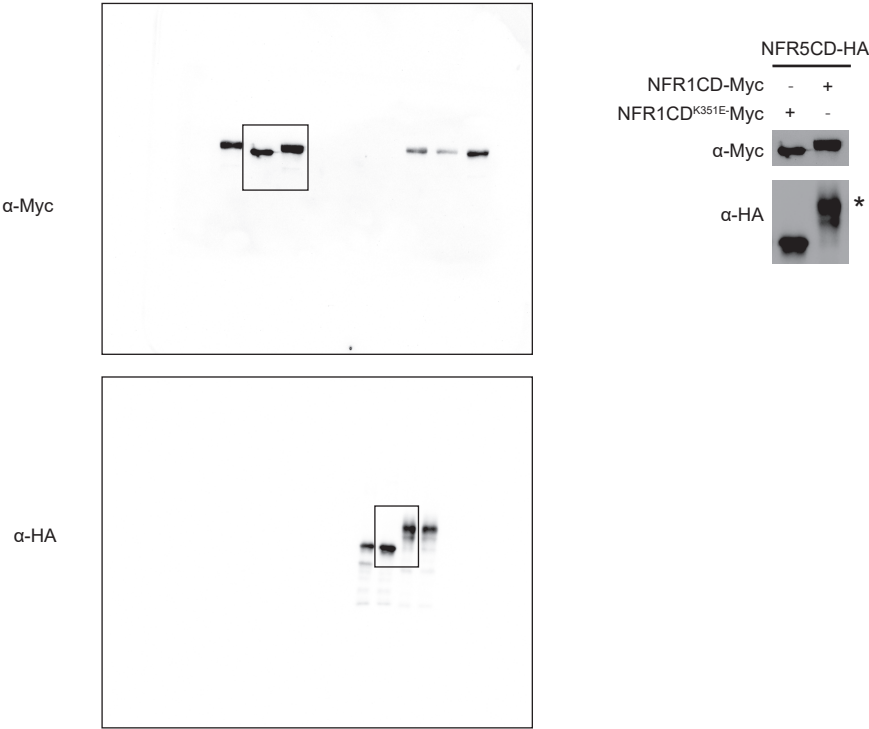

Supplement: Figure 4—figure supplement 1—source data 2. [file elife-97196-fig4-figsupp1-data2.pdf]

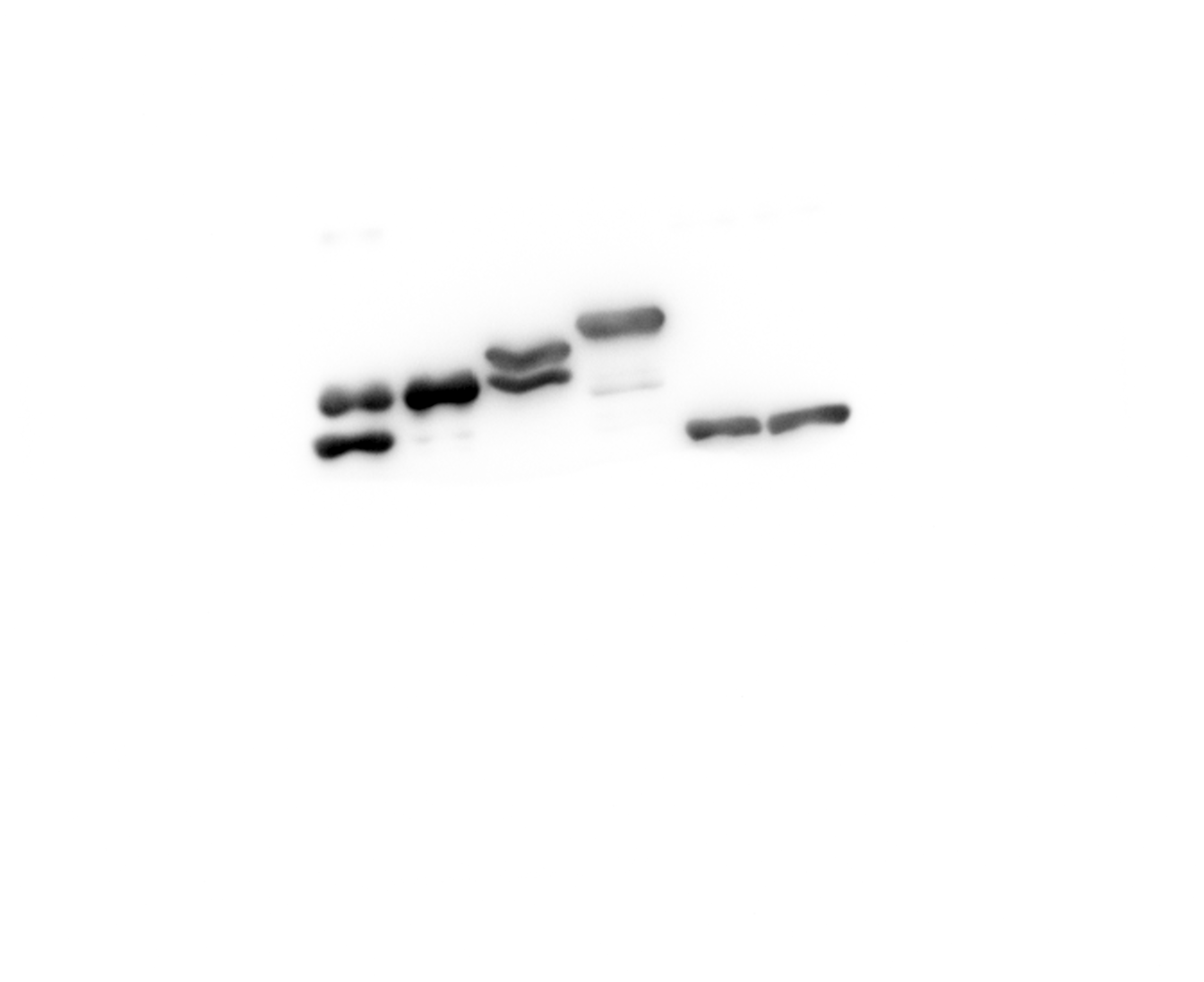

Supplement: Figure 6—source data 1. [file elife-97196-fig6-data1.zip › fig6A a-flag.tif]

Fig. 6A

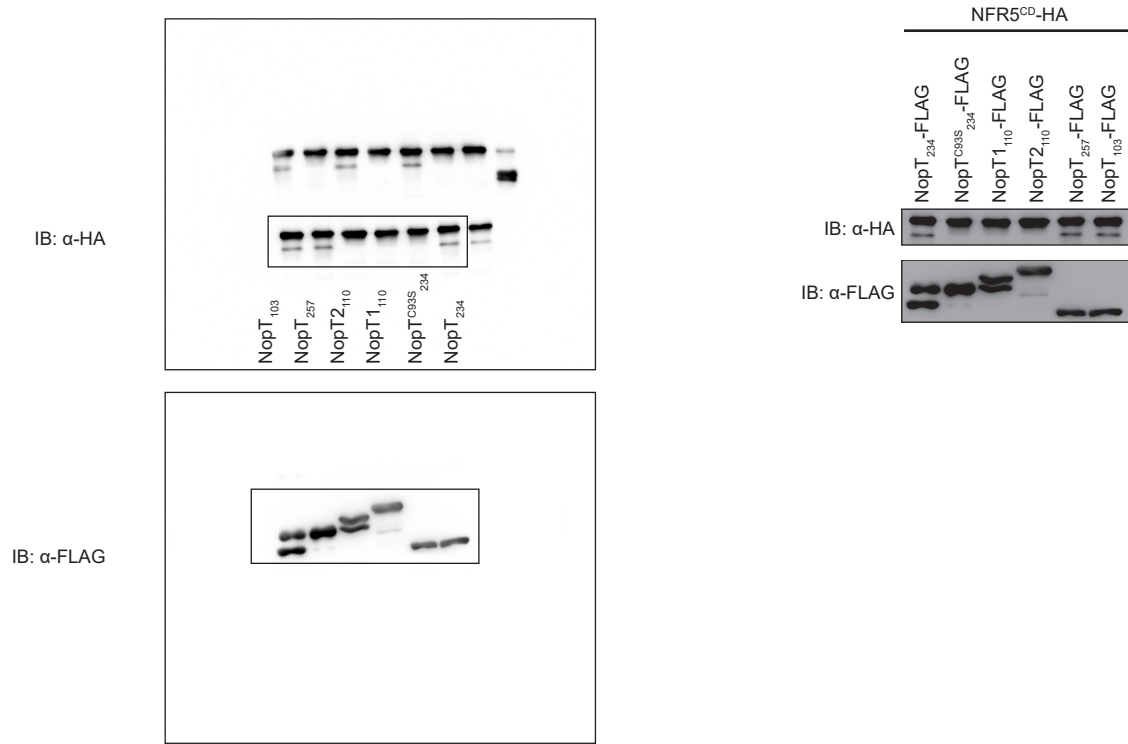

Supplement: Figure 6—source data 2. [file elife-97196-fig6-data2.pdf]
