# Supplementary material for: The rhizobial effector NopT targets Nod factor receptors to regulate symbiosis in Lotus japonicus
Source: eLife. 2025 Apr 4;13:RP97196. doi: 10.7554/eLife.97196 (PMC11970910; doi:10.7554/eLife.97196)
Supplement: Supplementary file 1. — (A) Phosphopeptides identified by liquid chromatography–mass spectrometry. (B) Oligonucleotides used in the study. [file elife-97196-supp1.docx]

**Supplementary Files**

**Supplemental File 1a.** **Phosphopeptides identified by liquid chromatography–mass spectrometry.** An *in vitro* kinase assay was performed using the CD of NFR1 and NopT. NopT was separated by SDS-PAGE, and the digested gel slices representing the phosphorylated NopT were used for analysis. The deduced phosphopeptides are listed.


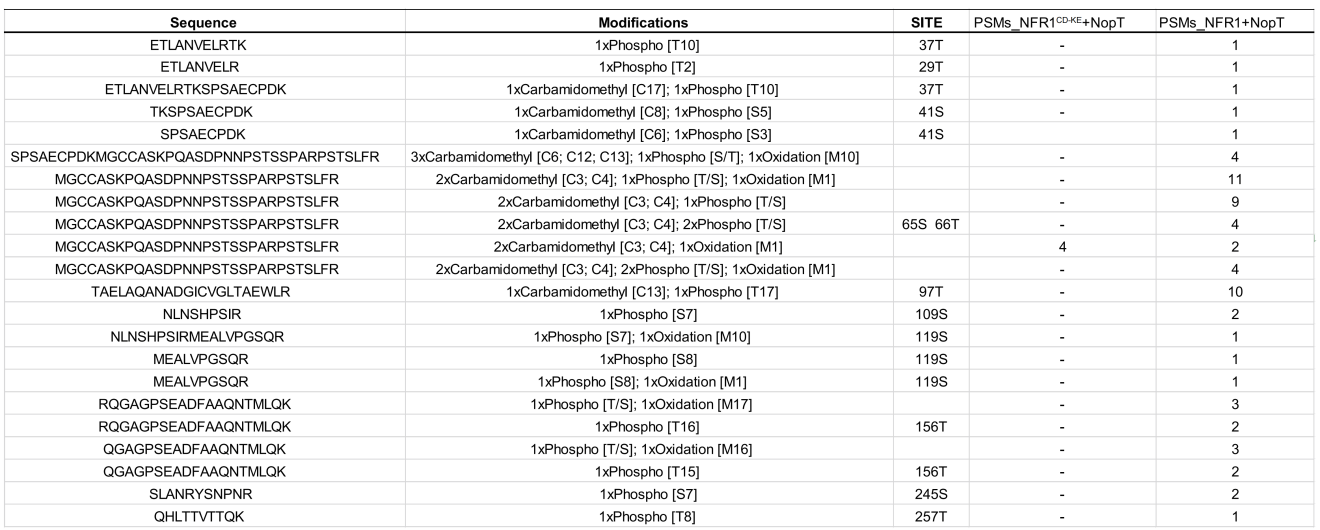


**Supplemental File 1b. Primers used in this study.**

| **Used for** | **Target genes** | **Names** | **Sequences (from 5’ to 3’)** |
| --- | --- | --- | --- |
| Overexpression in Nicotiana benthamiana | NopT or NopT^C93S^ | p5X-NopT-F | agaacacgggggactctagaatgcacagtcccatcag |
|  |  | p5X-NopT-R | ccgctgttatcggtacctgtcatcttttgggtggtcac |
|  | NopT^ΔN50^ | p5X-NopTdeN50-F | agaacacgggggactctagaatgtgctgcgccagcaag |
|  | NFR1 | p5X-NFR1-F | agaacacgggggactctagaatgaagctaaaaactggtctact |
|  |  | p5X-NFR1-R | ccgctgttatcggtacctcttctcacagac |
|  | NFR5 | p5X-NFR5-F | agaacacgggggactctagaatggctgtcttctttcttacc |
|  |  | p5X-NFR5-R | ccgctgttatcggtaccacgtgcagtaat |
|  |  | p5X-NFR5gfp-R | tcccgggagcggtaccacgtgcagtaatggaagt |
|  | AvrPphB | p5X-AvrPphB-F | agaacacgggggactctagaatgaaaataggtacgcaggcc |
|  |  | p5X-AvrPphB-R | ccgctgttatcggtacccgaaactctaaactcgtttacgc |
| templates for protein expression | NopT_USDA257_ | p5X-257T-F | agaacacgggggactctagatgctgcgtgatccgaacaat |
|  |  | p5X-257T-R | ccgctgttatcggtacctgtcaccttttgggtggtcaccg |
|  | NopT1_USDA110_ | p5X-110T1-F | agaacacgggggactctagaatgtatgatcgaatcggtgg |
|  |  | p5X-110T1-R | ccgctgttatcggtaccctgcatcctttgcgtcg |
|  | NopT2_USDA110_ | p5X-110T2-F | agaacacgggggactctagaatgtataatcgagtcgatggc |
|  |  | p5X-110T2-R | ccgctgttatcggtaccccgatgaggttccgc |
| Protein Expression in *E.coli* | NopT or NopT^C93S^ | 28a NopT-F | tgggtcgcggatccgaattcatgcacagtcccatcag |
|  |  | 28a FLAG-R | tcgagtgcggccgcaagcttcttgtcatcgtcatccttgtag |
|  |  | 28a Strep-R | tcgagtgcggccgcaagctttttttcaaattgaggatgagaccatcc |
|  | AvrPphB | ET28a-pphb-F | tgggtcgcggatccgaattcaaaataggtacgcaggccac |
|  | NopT_USDA257_ | ET28a-257T-F | tgggtcgcggatccgaattcctgcgtgatccgaacaa |
|  | NopT1_USDA110_ | ET28a-110T1-F | tgggtcgcggatccgaattctatgatcgaatcggtggctc |
|  | NopT2_USDA110_ | ET28a-110T2-F | tgggtcgcggatccgaattctataatcgagtcgatggcgaatac |
|  | NFR1^CD^ | duet NFR1-F | accacagccaggatccgagataccagaagaaggaagaagagaaagc |
|  |  | duet Sac1nost-R | ggcgcgccgagctcgcgatcggggaaattcgagct |
|  | NFR5^CD^ | ACSH-NFR5-F | agaagcgcggatccgaattctatgtatactgccgcagaaagaag |
|  |  | ACSH-NFR5-R | cgtatgggtagcttccaagcttacgtgcagtaatggaagtc |
|  | NFR5^KD^ | SUMO 5KD-F | ccgcgaacagattggtaaggttggggaatcagtgtac |
|  |  | SUMO HA-R | gctcgaattcggatcctttcacgcatagtcaggaacatcg |
|  | NFR5^JM^ | SUMO 5JM-F | ccgcgaacagattggttatgtatactgccgcagaaagaag |
|  |  | SUMO 5JM-R | gctcgaattcggatcctttcacgcatagtcaggaacatcg |
|  | sumo-NFR5^KD^-HA | duet sumo-F | catcaccacagccaggatgggtccctgcaggac |
|  |  | duet HA-R | cattatgcggccgcaagcttttacgcatagtcaggaacatcg |
|  | sumo-NFR5^JM^-GFP | duet sumo-F | catcaccacagccaggatgggtccctgcaggac |
|  |  | duet GFP-R | atgcggccgcaagcttttacttgtacaactcatccatacc |
|  | AtLYK5 | ACSH-LYK5-F | gcgcggatccgaattctacaaacgaaggtctaagaag |
|  |  | ACSH-LYK5-R | ggtagcttccaagcttgttgccaagagagccg |
|  | LjLYS11 | ACSH-LYS11-F | agaagcgcggatccgaattcacttgtctgaggaagagaaag |
|  |  | ACSH-LYS11-R | cgtatgggtagcttccaagcttacgagctgctatcaaagtt |
|  | LYK5^JM^ NFR5^KD^ | LYK5-NFR5-F | cgaaaacagaaaggttggggaatcagtg |
|  |  | NFR5 LYK5-R | ccccaacctttctgttttcgtcgctgaaatt |
|  | LYS11^JM^ NFR5KD | LYS11 NFR5-F | gagcagtgtaaggttggggaatcag |
|  |  | NFR5 LYS11R | ccccaaccttacactgctcattgagg |
|  | NFP^JM^ | ACSUG NFPJ-F | gtggaggcgctagaggatcctattgtctcaaaatgaagagattgaatagaag |
|  |  | ACSUG NFPJ-R | cttctcccttagagagctcacaattgtcactcagattcattg |
|  | NFR5^JM^-NFP^KD^ | 5JM-PKD-F | gatgagtgcaagattggtgaatcagtttacaaag |
|  |  | 5JM-PKD-R | accaatcttgcactcatcgct |
|  | NFP^JM^-NFR5^KD^ | PJM-5KD-F | gacaattgtaaggttggggaatcagtg |
|  |  | PJM-5KD-R | ccccaaccttacaattgtcactcagattcattg |
|  | NFR5^268-445^-NFP^458-595^ | 51/2KD-P1/2KD-F | ccatggccagaacttcaaccaactcaat |
|  |  | 51/2KD-P1/2KD-R | gaagttctggccatggcgaa |
|  | NFP^270-457^-NFR^5456-595^ | P1/2KD-51/2KD-F | ggatggctagaacttcgaccaac |
|  |  | P1/2KD-51/2KD-R | cgaagttctagccatcccgaa |
| site mutantions construction | NopT | T93S-F | gaatctccgtcggcttgactgc |
|  |  | T93S-F | gacggagattccatctgcgttc |
|  |  | 97D-F | ggcttggatgcggagtggctgcg |
|  |  | 97D-R | ctccgcatccaagccgacgcag |
|  |  | 97A-F | ggcttggctgcggagtggctgc |
|  |  | 97A-R | ctccgcagccaagccgacg |
|  |  | 109D-F | catccggatatccgaatggaggccctag |
|  |  | 109D-R | ttcggatatccggatgactgttgaggttacgc |
|  |  | 109A-F | catccggcaatccgaatggag |
|  |  | 109A-R | ttcggattgccggatgactgttg |
|  |  | 119D-R | cccggagatcaaaggcacgcctcagc |
|  |  | 119A-F | gcctttgatctccgggtactagggcc |
|  |  | 119A-F | cccggagcgcaaaggcac |
|  |  | 119A-R | ctttgcgctccgggtactagg |
|  |  | 156D-F | gcaaaacgatatgttgcagaaagcaggc |
|  |  | 156D-R | gcaacatatcgttttgcgccgcgaagtcggcc |
|  |  | 156A-F | gcaaaacgctatgttgcagaaagcag |
|  |  | 156A-R | ctgcaacatagcgttttgcgccgcgaagtc |
|  |  | H205A-F | ggcggcaacaccgttgcgacc |
|  |  | H205A-R | caacggtgttgccgccgccctcagc |
|  |  | D220A-F | ctcttcgctcctaatttcggcgaatttac |
|  |  | D220A-R | gccgaaattaggagcgaagagcgtggtgtttc |
|  |  | 245D-F | cgctacgacaatccaaaccggcag |
|  |  | 245D-R | ttggattgtcgtagcgattggctaggct |
|  |  | 257 mutants | changing the sequence of r primer at noptt257when cloned into vectors |
|  | NFR1 | K351E-F | gcaattgagaagatggatgtacaagcatc |
|  |  | K351E-R | catccatcttctcaattgctgttttcttgcc |
|  | NFR5 | 269-271A-F | ctatgcagctgcacgcagaaagaaggctctg |
|  |  | 269-271A-R | gcgtgcagctgcatagaattcggatccgcgc |
|  |  | 272-274A-F | ctgcgctgcagctaaggctctgaataggactgc |
|  |  | 272-274A-R | cttagctgcagcgcagtatacatagaattcggatcc |
|  |  | 275-277A-F | gaaaggcagctgcaaataggactgcttcatcagc |
|  |  | 275-277A-R | ctatttgcagctgcctttctgcggcagtatacatag |
|  |  | 278-280A-F | ctggctgcagctgcttcatcagctgagactg |
|  |  | 278-280A-R | agcagctgcagccagagccttctttctgcg |
|  |  | 282-283A-F | gcagcagctgagactgctgataaactac |
|  |  | 282-283A-R | gcagtctcagctgctgcagcagtcctattcagag |
|  |  | 285-286A-F | ggggctgctgataaactactttctggag |
|  |  | 285-286A-R | agtagtttatcagcagccccagctgatgaagcagtc |
|  |  | 288-290A-F | gctgcagcactttctggagtttcaggctatgtaag |
|  |  | 288-290A-R | aaactccagaaagtgctgcagcagcagtctcagctgatgaag |
|  |  | 291-294A-F | gagactgctgataaactagctgctgcagtttcaggctatgtaagcaagcc |
|  |  | 291-294A-R | atagcctgaaactgcagcagctagtttatcagcagtctcagctg |
|  |  | 295-298A-F | gagttgcagctgctgtaagcaagccaaacgtgtatg |
|  |  | 295-298A-R | cttacagcagctgcaactccagaaagtagtttatcagcag |
|  |  | 298-300A-F | ctatgcagctgcaccaaacgtgtatgaaatcgacg |
|  |  | 298-300A-R | gtttggtgcagctgcatagcctgaaactccagaaagtag |
|  |  | 301-303A-F | gcaaggcagctgcttatgaaatcgacgagataatggaagc |
|  |  | 301-303A-R | cataagcagctgccttgcttacatagcctgaaactcc |
|  |  | 304-306A-F | gtggctgcagctgacgagataatggaagctacgaag |
|  |  | 304-306A-R | cgtcagctgcagccacgtttggcttgcttacatagc |
|  |  | 307-309A-F | gaaatcgctgcagcaatggaagctacgaaggatttcag |
|  |  | 307-309A-R | ccattgctgcagcgatttcatacacgtttggcttgc |
|  |  | 310-312A-F | cgagatagcagctgctacgaaggatttcagcg |
|  |  | 310-312A-R | gtagcagctgctatctcgtcgatttcatacacgtttg |
|  |  | 313-315A-F | gctgcagctgcattcagcgatgagtgcaagg |
|  |  | 313-315A-R | ctgaatgcagctgcagcttccattatctcgtcgatttc |
|  |  | 316-318A-F | ggatgcagctgctgagtgcaaggttggggaatc |
|  |  | 316-318A-R | ctcagcagctgcatccttcgtagcttccattatctc |
|  |  | 319-321A-F | cgatgctgcagctgttggggaatcagtgtacaagg |
|  |  | 319-321A-R | caacagctgcagcatcgctgaaatccttcgtagc |
|  |  | 322-324A-F | gcaaggcagctgcatcagtgtacaaggccaacatag |
|  |  | 322-324A-R | gatgcagctgccttgcactcatcgctgaaatc |
|  |  | 325-327A-F | gaagcagctgcaaaggccaacatagaaggtcg |
|  |  | 325-327A-R | gcctttgcagctgcttccccaaccttgcactc |
|  |  | 268-277DE-F | agaagcgcggatccgaattcaataggactgct |
|  |  | 268-277DE-R | ctgatgaagcagtcctattgaattcggatccgcgcttc |
|  |  | 278-287DE-F | gcagaaagaaggctctggataaactactttctggagtttcagg |
|  |  | 278-287DE-R | aaactccagaaagtagtttatccagagccttctttctgcg |
|  |  | 298-307DE-F | caggctatgagataatggaagctacgaaggatttc |
|  |  | 298-307DE-R | cttccattatctcatagcctgaaactccagaaagtag |
|  |  | 308-317DE-F | gaaatcgacgatgagtgcaaggttgggg |
|  |  | 308-317DE-R | ttgcactcatcgtcgatttcatacacgtttggc |
|  |  | 318-327DE-F | gatttcagcaaggccaacatagaaggtcg |
|  |  | 318-327DE-R | gttggccttgctgaaatccttcgtagcttcc |
|  |  | 328-337DE-F | tcagtgtacgtaaagaaaatcaaggaaggtggtgcc |
|  |  | 328-337DE-R | attttctttacgtacactgattccccaaccttg |
|  |  | 288-294A-F | gcagctgccgcagcggctgcctcaggctatgtaagcaagcc |
|  |  | 288-294A-R | gcagccgctgcggcagctgcagcagtctcagctgatgaagc |
|  |  | 288-298PBS1-F | ggagacaaatctcatgtctcctcaggctatgtaagcaagcc |
|  |  | 288-298PBS1-R | ggagacatgagatttgtctccagcagtctcagctgatgaagc |
|  |  | N5-283S-R | tgaagcagtcctattcagagcc |
|  |  | 283Y-F | tctgaataggactgcttcatacgctgagactgc |
|  |  | N5-294G | agaaagtagtttatcagcagtctcag |
|  |  | 294Q-F | ctgctgataaactactttctcaggtttcaggctatgtaag |
|  |  | N5-303Y-R | cacgtttggcttgcttacatag |
|  |  | 304S-F | gcaagccaaacgtgtccgaaatcgacgag |
|  |  | N5-310AT-R | ttccattatctcgtcgatttcatacac |
|  |  | 311-2 IY-F | gaaatcgacgagataatggaaatctacaaggatttcagcgatgagtg |
| Complemental | NopT | HC-proT-F | gaattcgagctcggtacctcatggccttccttggagg |
|  |  | T-3-F | cacagtcccatcagtggttc |
|  |  | T-3-R | gaaccactgatgggactgtg |
|  |  | HC-T-R | cgcgggatcgagatctcgagtcatgtcatcttttgggtggtc |
|  | NFR5 | cherry-proNFR5-F | ccaagctgggctgcagggacatgagattgaagctcc |
|  |  | proNFR5-NFR5-F | ccccacttcacaaacatggctgtcttctttcttacctc |
|  |  | proNFR5-NFR5-R | gccatgtttgtgaagtgggg |
|  |  | NFR5-JM-KD-F | gatgagtgcaaggttgggg |
|  |  | NFR5-JM-KD-R | ccccaaccttgcactcatc |
|  |  | cherry kpn1 Sac1-R | ggcgcgcctaggtaccggggaaattcgagc |
